# Regioselective Hydrosilylation Catalysis with Supported Well-Defined Pt(0) Complexes: Effects of Surface Anions and Phosphenium Ligands

**DOI:** 10.1021/acs.inorgchem.5c05042

**Published:** 2026-02-16

**Authors:** Damien B. Culver, Conor Neill, Frédéric A. Perras, Mita Halder, Angela Chartouni

**Affiliations:** † Division of Chemical and Biological Sciences, Ames National Laboratory, Ames, Iowa 50011, United States; ‡ Department of Chemistry, Iowa State University, Ames, Iowa 50011, United States

## Abstract

Achieving stable, selective single-atom catalysts is challenging because localsurface-site structures are difficult to control. Surface organometallic chemistry and organic–inorganic hybrid materials offer partial solutions, but applications to supporting zero-valent metals are limited. We demonstrate that well-defined, ionically bound N-heterocyclic phosphenium ([NHP]^+^) ligands can be generated on silylium-functionalized sulfated zirconia ([^i^Pr_3_Si]­[SZO]). These surface-bound [NHP]­[SZO] ligands coordinate Pt(0) centers, forming [(NHP)­Pt(0)­L]­[SZO] precatalysts that are highly active for alkyne hydrosilylation. Systematic studies reveal that sterically bulky aromatic ligands enhance regio­selectivity, achieving performances comparable to molecular Pt catalysts. Further, more-coordinating anions support more-regio­selective precatalysts; therefore, SZO supports more-selective species than weaker-coordinating Al­(OC­(CF_3_)_3_)_3_-functionalized silica, a trend confirmed by molecular analogues. These results demonstrate that both the ligand and support control catalytic behavior and enable solid-state structure–activity relationships.

## Graphical abstract

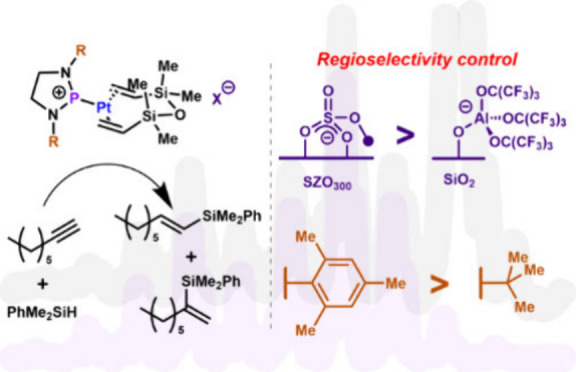

The formation of selective heterogeneous single-atom catalysts (SACs) remains a significant challenge, particularly for low-valent transition metals, which are generally unstable on support surfaces.^1–7^ A key example is Pt(0) SACs, which are difficult to stabilize for reactions such as regio­selective alkyne hydrosilylation.^8–12^ Conventional oxide-supported Pt particle or single-atom hydrosilylation catalysts typically require high metal loadings and fail to match the selectivity of molecular catalysts. This is partially attributable to the plurality of active sites in these systems, which prevents the structural control required to develop and leverage structure–activity relationships (SARs).

Surface organometallic chemistry (SOMC) combines molecular and surface strategies to create well-defined heterogeneous catalysts, primarily for non-zero-valent metals.^13–20^ While SOMC has enabled selective catalysis, including limited examples of alkyne and alkene hydrosilylation,^21–25^ SOMC typically relies on chemisorption of M-X species via protonolysis, halide abstraction,^26,27^ or more recently addition of zero-valent metals through oxidative grafting (Figure 1A).^28–32^ All of these result in metals in oxidation states greater than zero. Supporting zero-valent metals remains challenging, as reducing conditions often yield nanoparticles.^33–37^ Molecular ligands stabilize zero-valent metals; functionalizing supports with these ligands should enable anchoring such species and studying structure–activity relationships in SACs. Ligands tethered to polymers, graphene, or oxides form organic–inorganic hybrid materials (OIHMs), such as N-heterocyclic carbenes (NHCs) on silica, which coordinate metals for heterogeneous catalysis (Figure 1B).^38–40^ However, these syntheses are costly, labor-intensive, and limited to non-zero-valent metals.

**Figure 1:**
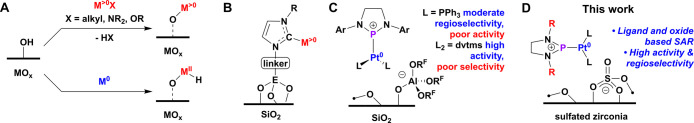
Available methods for chemisorption of metals to oxide surfaces: protonolysis and oxidative grafting (**A**),^28–32^ coordination to OIHMs (**B**),^38–40^ Pt(0) coordination with electrostatically bound [NHP]^+^ ligands on the surface of functionalized silica (ASO) (**C**),^41^ and SZO (**D**, this work).

We showed that N-heterocyclic phosphenium ([NHP]^+^) ligands^42–45^ can be generated on Lewis-acid-functionalized silica (ASO) without a tether, enabling the first well-defined Pt(0) complexes for alkyne hydrosilylation (Figure 1C).^41^ These precatalysts are highly active but poorly regio­selective at low loadings compared to molecular NHC-Pt analogues, likely due to limited stability and electronic differences.^43^ We hypothesized that alternative [NHP]^+^ ligands could improve the regio­selectivity and strengthen the P–Pt bond. Because functionalized silica decomposes at elevated temperatures,^46,47^ we replaced it with thermally stable sulfated zirconia (SZO), which also enabled the study of support effects (Figure 1D).^16,48,49^

[NHP]­[SZO] ligands were synthesized by chloride abstraction from NHPCl^50–53^ precursors using [^i^Pr_3_Si]­[SZO] ^54,55^ (Figure 2A), following the approach for [DippNHP]­[ASO].^41^ ICP-OES and ^i^Pr_3_SiCl quantification confirmed P loadings of 0.067–0.136 mmol/g, consistent with ∼1 [^i^Pr_3_Si] site reacting per P (Table S1). Compounds **1a**–**e** were characterized by ^1^H, ^13^C, and ^31^P solid-state (SS)­NMR spectroscopy in addition to 2D ^1^H homonuclear double-quantum/single-quantum (DQ/SQ) correlation. The ^31^P spectrum of **1d** (Figure 2B) and others in the Supporting Information (SI) contain signals at 252–277 ppm, comparable to those of [DippNHP]­[ASO] (^31^P δ = 272 ppm) and molecular analogues.^51,56^ ^1^H and ^13^C SSNMR spectra further support the proposed structures.

**Figure 2:**
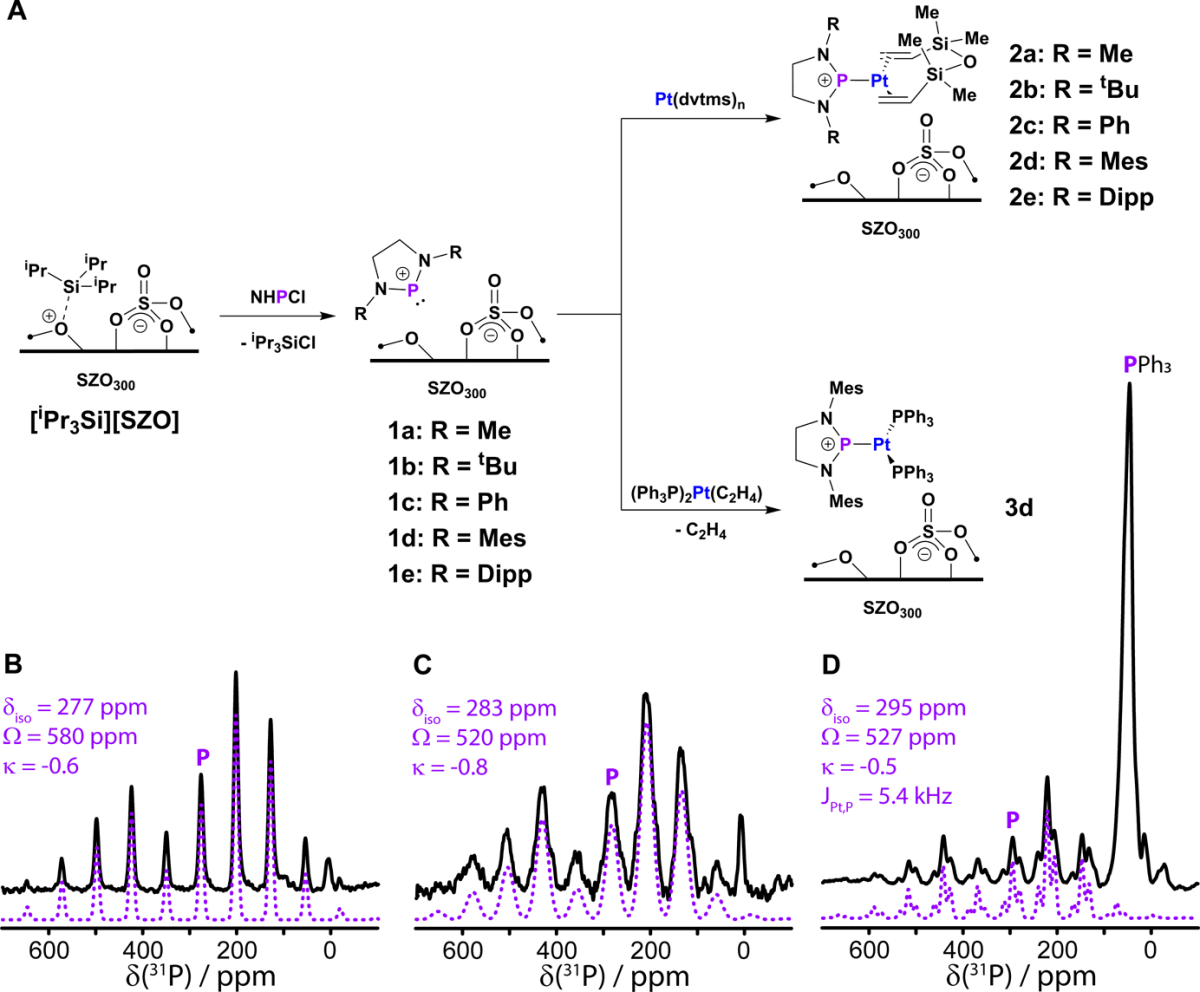
General synthetic strategy and proposed structures for **1a**–**e**, **2a**–**e**, and **3d** (**A**). ^31^P CPMAS SSNMR spectra of **1d** (**B**), **2d** (**C**), and **3d** (**D**). Purple dashed lines are best-fits of the experimental spectra. Isotropic chemical shifts are indicated by **P**. See SI for full details.

Treatment of **1a**–**e** with Karstedt’s catalyst^57^ (Figure 1A) generates well-defined [(NHP)­Pt­(dvtms)]­[SZO] sites analogous to previously reported ASO-supported species (see SI). ICP-OES analysis confirmed Pt coordination (0.038–0.081 mmol/g) and revealed a slight decrease in phosphorus content, likely due to the partial re-formation of NHPCl from reactions with physisorbed ^i^Pr_3_SiCl not fully removed during toluene washings. The P:Pt ratios are 0.9–1.9, with some samples containing unreacted [NHP]­[SZO] (see Table S1 and SSNMR in SI), and the dvtms:Pt ratios range from 0.6 to 1.3. Samples **2a** and **2b** are deficient in dvtms, which may result from bis-coordination of [NHP]^+^ ligands forming minor [(NHP)_2_Pt]­[SZO] sites, consistent with their high phosphorus loadings (>0.6 P/nm^2^). MesNHPOTf^51^ reacts with Karstedt’s catalyst to form [(NHP)­(NHP­(OTf))­Pt]­[OTf] in solution (see SI), supporting that bis-coordination is possible. In contrast, **2c**–**e** exhibit a dvtms:Pt ratio near 1.0, supporting the stoichiometry in Figure 2A.

**2a**–**e** were characterized by SSNMR spectroscopy, and the ^31^P SSNMR spectrum of **2d** is provided in Figure 2C as an example (see SI). In all cases, the ^31^P SSNMR spectra confirm the successful formation of the [(NHP)­Pt(0)­(dvtms)]^+^ species. The ^31^P SSNMR resonances are broadened by the interaction with Pt, and they all shift to higher ppm (277–292 ppm), making them comparable to those of other [(NHP)­Pt(0)­L_n_] cations.^41,51,56^ The broadening of the ^31^P NMR signal is likely due to a distribution in the strength of the Pt–P bonding or from the dynamics of the complexes. For instance, we see the appearance of millisecond-order dynamics in **2a** and **2c**, which prevents the uniform cross-polarization of the spinning sideband pattern, even at 100 K. As a result, our attempts at ^1^H-detecting the ^195^Pt NMR signals from these species using fast-MAS were unsuccessful.^58–60^ Indeed, even the ^1^H–^31^P dipolar interactions are undetectable at room temperature. The ^1^H SSNMR spectra acquired for **2a**–**e** (see SI) displayed resonances unique to the [NHP]^+^ and dvtms ligands, suggesting the structure is similar to (NHC)­Pt­(dvtms) complexes, as proposed (Figure 2A),^61^ although most clear double-quantum correlations were intraligand. We also synthesized [(MesNHP)­Pt(0)­(PPh_3_)_2_]­[SZO] (**3d**), which contains 0.045 mmol/g of Pt and 3.2 equiv of P (Figure 2A). The ^31^P SSNMR of **3d** (Figure 2D) is similar to that of [(DippNHP)­Pt(0)­(PPh_3_)_2_]­[ASO] and exhibits a resolvable *J*_Pt–P_ coupling of 5.4 kHz, comparable to spectra of molecular Pt(0) phosphenium complexes, confirming coordination.^41,56^

A major challenge in heterogeneous catalysis is achieving control over SARs. Precatalysts **2a**–**e** and **3d** exhibit tunable steric and electronic properties, offering a valuable platform for studying SARs. Precatalysts **2a**–**e** demonstrate high activity in the hydrosilylation of 1-octyne, achieving full conversion at loadings of 0.005–0.05 mol% Pt within 8 h (Table 1). Notably, activity and regio­selectivity vary systematically with precatalyst structure. Complexes bearing bulky ligands, **2b**, **2d**, and **2e**, exhibit high turnover frequencies (TOFs > 400 min^–1^ at 15 min), whereas less-bulky **2a** and **2c** are less than half as active. This trend suggests that reductive elimination is rate-limiting, paralleling molecular systems.^62^

**Table 1:**
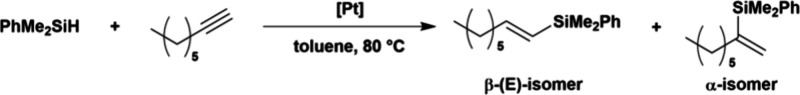
Summary of 1-Octyne Hydrosilylation Catalysis Results with Solid Precatalysts^a^

**Entry**	**Precatalyst**	**Pt loading** **(mol%)**	**Time (h)** ^b^	**Combined Yield (%)**	**Turnovers (×10** ^**3**^ **)**	**TOF @ ≤40% conv. (min** ^**–1**^ **)**	**Final** β-(E)/α
1	**2a**	0.005	8	95 (1)	19.0 (0.2)	127 (11)	6.5 (0.1)
2	**2b**	0.01	2	87.8 (0.2)	8.8 (0.1)	>73.2 (0.2)^c^	5.1 (0.1)
3	**2b**	0.005	4	93.2 (0.4)	18.6 (0.1)	775 (80)^d^	5.9 (0.1)
4	**2c**	0.005	8	94.5 (0.3)	18.9 (0.1)	207 (25)	6.8 (0.2)
5	**2d**	0.05	0.5^e^	85.3 (0.5)	1.71 (0.01)	56.9 (0.3)^c^	17.5 (0.1)
6	**2d**	0.01	2	89.2 (0.3)	8.9 (0.1)	>74.3 (0.3)^c^	12.9 (0.5)
7	**2d**	0.005	6	97.4 (0.7)	19.5 (0.1)	433 (9)	9.8 (0.1)
8	**2e**	0.01	2	88.7 (0.7)	8.9 (0.1)	>73.9 (0.6)^c^	9.8 (0.2)
9	**2e**	0.005	2	92.4 (0.3)	18.5 (0.1)	1161 (6)^f^	8.9 (0.2)
10	**3d**	0.45	21^g^	88 (9)	0.20 (0.02)	0.3 (0.1)	22.5 (0.9)
11	**3d**	0.045	47^h^	92 (1)	2.0 (0.1)	2.9 (0.1)	7.2 (0.1)
12	**3d**	0.0045	72^i^	85 (7)	19 (2)	22 (7)	5.4 (0.1)
13	**1d** ^j^	0	0.5	0	0	0	–

a Conditions: Precatalyst (5.0 or 25.0 mg), 0.5 M 1-octyne/PhMe_2_SiH in toluene stirring at 500 rpm at 80 °C. Average of 2 runs. Errors provided in parentheses. Turnovers = mol_product_/mol_Pt_. TOF = turnovers/time.

b Time to >99% silane consumption. See SI for reaction profiles and details.

c TOF at full conversion.

d 62 (5)% conversion at 15 min.

e 96.2 (0.1)% conversion.

f 93.9 (0.7)% conversion at 15 min.

g 91 (9)% conversion.

h 98% conversion.

i 91 (7)% conversion.

j Same conditions as entry 5, no substrate was consumed.

Notably, regio­selectivity correlates with ligand electronics and steric bulk. Electron-withdrawing aryl R groups in **2c**–**e** yielded the highest selectivities, with **2c** being slightly more selective than **2a** and **2b**, and **2d** being the most selective. **2b** shows selectivity similar to that of **2a** and lower than that of **2c** despite containing sterically larger ^t^Bu groups, highlighting the importance of bulky and electron-withdrawing ligands. Interestingly, **2e** is more selective than the ASO analogue (6.2–7),^41^ suggesting there may be an effect from the anionic support.

We observed a positive correlation between the regio­selectivity and precatalyst loading for **2d** and **2e**. Doubling the loadings from 0.005 to 0.01 mol% increases the β-(E)/α from 9.8 and 8.9 to 12.9 and 9.8 for **2d** and **2e**, respectively. Further increasing the **2d** loading to 0.05 mol% raises the β-(E)/α to 17.5. The absence of similar increases for **2b** suggests the higher selectivity of **2d** and **2e** may be due to kinetic effects involving alkyne orientation by bulky aryl ligands; similar effects have been observed in molecular complexes.^63^ **2d** and **2e** are the most active and regio­selective solid-state Pt alkyne hydrosilylation precatalysts reported to date^21,41,64,65^ and are comparable to the best molecular catalysts.^62,63,66–69^

Similarly, **3d** produced a regio­selectivity of 22.5 at 0.45 mol% Pt, double the ratio of [(DippNHP)­Pt­(PPh_3_)_2_]­[ASO].^41^ **3d** is also more active at lower Pt loadings, completing the reaction in 2 days at 0.045 mol% Pt, whereas [(DippNHP)­Pt­(PPh_3_)_2_]­[ASO] achieved only 22% yield under comparable conditions, showcasing **3d**’s improved stability. The regio­selectivity, nevertheless, dropped over time from an initial selectivity of 9.9 to a final ratio of 7.2 (see SI), unlike **2a**–**e**. A similar observation was made at 0.0045 mol% Pt. This likely reflects PPh_3_-induced leaching that forms poorly selective Pt particles.^8,9^

A significant challenge in the stabilization of single-atom Pt(0) and other low-valent metals is leach prevention.^8,9,12,70^ Low-coordinate Pt with small ligands, including the precursor Karstedt’s catalyst, produces β-(E)/α ratios of ∼5–6,^62^ so Pt released from [NHP]^+^ in **2a**–**e** would lower selectivity. Hot-filtration tests (0.05 mol% Pt) after partial silane consumption (<54%) at 80 °C showed continued and comparable product formation in filtrates, indicating the formation of soluble species (see SI). ICP-OES of the **2d** filtrate detected small amounts of Pt and Zr, and SEM images of SZO^55^ revealed <1 μm agglomerates, supporting that [NHP-Pt]^+^-bearing SZO particles leach into solution.

Further tests with **2d** (0.005 mol% Pt) confirmed that most particles are <0.1 μm, as Celite and fine frit filtration only modestly reduced filtrate activity. Heating **2d** with 1-octyne or PhMe_2_SiH before filtration and then adding the other substrate cut filtrate-catalyzed product yields to <50%, with the 1-octyne-contacted precatalyst filtrate being least active. This suggests that heat and silane disrupt SZO particle-particle cohesion, and silane may stabilize particles by reacting with pyrosulfates^71^ or residual hydroxyls. Catalyst reuse (0.08 mol% Pt) after partial silane conversion (44%) and hot-filtration showed ∼75% yield loss after one cycle and a gradual decline over four cycles, while regio­selectivity dropped slightly (14→12). Despite leaching, residual activity and maintained regio­selectivity indicate that the local structure persists and Pt clusters do not form.

The difference in regio­selectivity between products formed by **2e** and [(DippNHP)­Pt(0)­(dvtms)]­[ASO] suggests that the support plays a significant role in catalytic performance, beyond acting as a passive counterion. Prior studies showed that SZO surfaces coordinate cations more strongly than ASO.^46^ Similar support effects have been observed for SOMC catalysts supported on sulfated oxides.^31,72–74^ To evaluate the influence of the anion on catalysis, we generated molecular precatalyst analogues by combining Karstedt’s catalyst with [MesNHP]^+^ with a non- ([B­(C_6_F_5_)_4_]^−^) or weakly (OTf^–^) coordinating anion (see SI)^51^ and compared their regio­selectivities (Table 2). The OTf^–^ supported a more regio­selective catalyst than [B­(C_6_F_5_)_4_]^−^, comparable to precatalyst **2d**, indicating that more-coordinating anions enhance regio­selectivity. We further established that the ligand structural trend observed in **2a–e** applies to the molecular analogues, with smaller MeNHPOTf and PhNHPOTf generating less regio­selective catalysts than MesNHPOTf. Combinations of the ligand and Karstedt’s catalyst confirm coordination to form [NHP-Pt]^+^ species by NMR (see SI). SSNMR of **1d** and **1e** showed no evidence of surface interaction with the phosphorus cation, but it is plausible that sulfates coordinate to Pt or P during catalysis.^16,75,76^ These findings suggest that the surface sulfates may contribute to regio­selectivity by acting as hemilabile ligands.

**Table 2:**
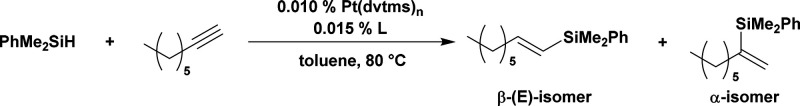
Summary of 1-Octyne Hydrosilylation Catalysis Results with *In Situ*-Generated Molecular Complexes^a^

**Ligand**	**^31^P δ (ppm)**	**Silane consumed (%)**	**Total yield (%)**	**Final** β-(E)/α
MeNHPOTf	264 ^77^	89 (1)	83.5 (0.6)	5.41 (0.05)
PhNHPOTf	173 ^78^	92 (2)	88 (2)	3.41 (0.04)
MesNHPOTf	203	>99	95.7 (0.6)	12.0 (0.4)
[MesNHP][B(C_6_F_5_)_4_]	276	>99	92.6 (0.2)	8.8 (0.3)

a Conditions: Ligand (0.015 mol%), Karstedt’s catalyst (0.010 mol%), 0.5 M 1-octyne/PhMe_2_SiH in toluene at 80 °C for 1 h. Average of 2 runs. Errors provided in parentheses.

In summary, sulfated zirconium oxide (SZO) supports well-defined [NHP]^+^ OIHM ligands that coordinate the Pt(0) centers for alkyne hydrosilylation. SAR studies reveal that sterically bulky ligands enhance catalytic activity, while aromatic ligands yield higher regio­selectivity than alkyl counterparts. Precatalysts **2d** and **2e** exhibit activities and selectivities comparable to those of molecular systems, positioning them as promising heterogeneous alternatives. Preliminary molecular studies suggest that the anion significantly influences regio­selectivity. These insights provide a foundation for designing next-generation OIHM catalysts. Ongoing work is focused on further exploring the role of the supporting anion and elucidating the mechanism of this emerging class of supported Pt(0) complexes.

## Safety Statements

***Caution!*** Extreme care should be taken in both the handling of the cryogenic liquid nitrogen and its use in the Schlenk line trap to avoid the condensation of oxygen from air.

***Caution!*** The GHS Category 1 corrosive and toxic sulfuric acid (also oxidizing), hydrofluoric acid, nitric acid (also oxidizing), hydrochloric acid, and phosphorus trichloride constitute significant safety hazards and must be handled with extreme care.

## Supplementary Material



## Data Availability

Raw data are available at https://zenodo.org (DOI: 10.5281/zenodo.18435632).

## Notes

Precatalyst synthesis and characterization and catalysis data (PDF)

## References

[1] Ji S., Chen Y., Wang X., Zhang Z., Wang D., Li Y. (2020). Chemical Synthesis of Single Atomic Site Catalysts. Chem. Rev..

[2] Kaiser S. K., Chen Z., Faust Akl D., Mitchell S., Pérez-Ramírez J. (2020). Single-Atom
Catalysts across the Periodic Table. Chem. Rev..

[3] Li J., Stephanopoulos M. F., Xia Y. (2020). Introduction: Heterogeneous Single-Atom
Catalysis. Chem. Rev..

[4] Mitchell S., Pérez-Ramírez J. (2020). Single Atom
Catalysis: A Decade of
Stunning Progress and the Promise for a Bright Future. Nat. Commun..

[5] Zhang Z., Li H., Wu D., Zhang L., Li J., Xu J., Lin S., Datye A. K., Xiong H. (2022). Coordination
Structure at Work: Atomically
Dispersed Heterogeneous Catalysts. Coord. Chem.
Rev..

[6] Endo K., Saruyama M., Teranishi T. (2023). Location-Selective
Immobilisation
of Single-Atom Catalysts on the Surface or within the Interior of
Ionic Nanocrystals Using Coordination Chemistry. Nat. Commun..

[7] Fang J., Chen Q., Li Z., Mao J., Li Y. (2023). The Synthesis
of Single-Atom Catalysts for Heterogeneous Catalysis. Chem. Commun..

[8] Troegel D., Stohrer J. (2011). Recent Advances and
Actual Challenges in Late Transition
Metal Catalyzed Hydrosilylation of Olefins from an Industrial Point
of View. Coord. Chem. Rev..

[9] Pagliaro M., Ciriminna R., Pandarus V., Béland F. (2013). Platinum-Based
Heterogeneously Catalyzed Hydrosilylation. Eur.
J. Org. Chem..

[10] Meister T. K., Riener K., Gigler P., Stohrer J., Herrmann W. A., Kühn F. E. (2016). Platinum
Catalysis Revisited—Unraveling Principles
of Catalytic Olefin Hydrosilylation. ACS Catal..

[11] Shimada, S. Recent Advances of Group 10 Transition Metal Hydrosilylation Catalysts. In Perspectives of Hydrosilylation Reactions; Marciniec, B. , Maciejewski, H. , Eds.; Springer Nature: Cham, Switzerland, 2023; pp 13–93. 10.1007/3418_2023_99.

[12] Yang H., Zhou Z., Tang C., Chen F. (2024). Recent Advances in
Heterogeneous Hydrosilylation of Unsaturated Carbon-Carbon Bonds. Chin. Chem. Lett..

[13] Copéret C., Comas-Vives A., Conley M. P., Estes D. P., Fedorov A., Mougel V., Nagae H., Núñez-Zarur F., Zhizhko P. A. (2016). Surface Organometallic and Coordination Chemistry toward
Single-Site Heterogeneous Catalysts: Strategies, Methods, Structures,
and Activities. Chem. Rev..

[14] Pelletier J. D. A., Basset J.-M. (2016). Catalysis by Design:
Well-Defined Single-Site Heterogeneous
Catalysts. Acc. Chem. Res..

[15] Samantaray M. K., Pump E., Bendjeriou-Sedjerari A., D’Elia V., Pelletier J. D. A., Guidotti M., Psaro R., Basset J.-M. (2018). Surface
Organometallic Chemistry in Heterogeneous Catalysis. Chem. Soc. Rev..

[16] Witzke R. J., Chapovetsky A., Conley M. P., Kaphan D. M., Delferro M. (2020). Nontraditional
Catalyst Supports in Surface Organometallic Chemistry. ACS Catal..

[17] Samantaray M. K., D’Elia V., Pump E., Falivene L., Harb M., Ould Chikh S., Cavallo L., Basset J.-M. (2020). The Comparison between
Single Atom Catalysis and Surface Organometallic Catalysis. Chem. Rev..

[18] Samantaray M. K., Mishra S. K., Saidi A., Basset J.-M. (2021). Surface
Organometallic
Chemistry: A Sustainable Approach in Modern Catalysis. J. Organomet. Chem..

[19] Copéret, C. ; Korzyński, M. D. 6.04 - Surface Organometallic and Coordination Chemistry Approach to Formation of Single Site Heterogeneous Catalysts. In Comprehensive Inorganic Chemistry III, 3rd ed.; Reedijk, J. , Poeppelmeier, K. R. , Eds.; Elsevier: Oxford, 2023; pp 67–85. 10.1016/B978-0-12-823144-9.00003-0.

[20] Samudrala K., Conley P. (2023). M. Effects of Surface
Acidity on
the Structure of Organometallics Supported on Oxide Surfaces. Chem. Commun..

[21] Mollar-Cuni A., Borja P., Martin S., Guisado-Barrios G., Mata J. A. (2020). A Platinum Molecular Complex Immobilised
on the Surface
of Graphene as Active Catalyst in Alkyne Hydrosilylation. Eur. J. Inorg. Chem..

[22] Sánchez-Page B., Jiménez M. V., Pérez-Torrente J. J., Passarelli V., Blasco J., Subias G., Granda M., Álvarez P. (2020). Hybrid Catalysts
Comprised of Graphene Modified with Rhodium-Based N-Heterocyclic Carbenes
for Alkyne Hydrosilylation. ACS Appl. Nano Mater..

[23] Panyam P. K. R., Atwi B., Ziegler F., Frey W., Nowakowski M., Bauer M., Buchmeiser M. R. (2021). Rh­(I)/(III)-N-Heterocyclic Carbene
Complexes: Effect of Steric Confinement Upon Immobilization on Regio-
and Stereo­selectivity in the Hydrosilylation of Alkynes. Chem. – Eur. J..

[24] Acikalin H., Panyam P. K. R., Shaikh A. W., Wang D., Kousik S. R., Atanasova P., Buchmeiser M. R. (2023). Hydrosilylation of Alkynes Under
Continuous Flow Using Polyurethane-Based Monolithic Supports with
Tailored Mesoporosity. Macromol. Chem. Phys..

[25] Kanbur U., Witzke R. J., Xu J., Ferrandon M. S., Goetjen T. A., Kropf A. J., Perras F. A., Liu C., Tilley T. D., Kaphan D. M., Delferro M. (2023). Supported Electrophilic
Organoruthenium Catalyst for the Hydrosilylation of Olefins. ACS Catal..

[26] Gao J., Dorn R. W., Laurent G. P., Perras F. A., Rossini A. J., Conley M. P. (2022). A Heterogeneous
Palladium Catalyst for the Polymerization
of Olefins Prepared by Halide Abstraction Using Surface R3Si+ Species. Angew. Chem., Int. Ed..

[27] Rodriguez J., Conley M. P. (2022). A Heterogeneous
Iridium Catalyst for the Hydroboration
of Pyridines. Org. Lett..

[28] Kaplan A. W., Bergman R. G. (1998). Nitrous Oxide Mediated
Synthesis of Monomeric Hydroxoruthenium
Complexes. Reactivity of (DMPE)­2Ru­(H)­(OH) and the Synthesis of a Silica-Bound
Ruthenium Complex. Organometallics.

[29] Maier S., Cronin S. P., Vu Dinh M.-A., Li Z., Dyballa M., Nowakowski M., Bauer M., Estes D. P. (2021). Immobilized
Platinum
Hydride Species as Catalysts for Olefin Isomerizations and Enyne Cycloisomerizations. Organometallics.

[30] Hall J. N., Chapovetsky A., Kanbur U., Kim Y. L., McCullough K. E., Syed Z. H., Johnson C. S., Ferrandon M. S., Liu C., Kropf A. J., Delferro M., Kaphan D. M. (2023). Oxidative Grafting
for Catalyst Synthesis in Surface Organometallic Chemistry. ACS Appl. Mater. Interfaces.

[31] Chang A. S., Kascoutas M. A., Valentine Q. P., How K. I., Thomas R. M., Cook A. K. (2024). Alkene Isomerization Using a Heterogeneous Nickel-Hydride
Catalyst. J. Am. Chem. Soc..

[32] Atterberry B. A., Wimmer E. J., Klostermann S., Frey W., Kästner J., Estes D. P., Rossini A. J. (2025). Structural
Characterization of Surface
Immobilized Platinum Hydrides by Sensitivity-Enhanced 195Pt Solid
State NMR Spectroscopy and DFT Calculations. Chem. Sci..

[33] Laurent P., Veyre L., Thieuleux C., Donet S., Copéret C. (2013). From Well-Defined
Pt­(II) Surface Species to the Controlled Growth of Silica Supported
Pt Nanoparticles. Dalton Trans..

[34] Héroguel F., Gebert D., Detwiler M. D., Zemlyanov D. Y., Baudouin D., Copéret C. (2014). Dense and
Narrowly Distributed Silica-Supported
Rhodium and Iridium Nanoparticles: Preparation via Surface Organometallic
Chemistry and Chemisorption Stoichiometry. J.
Catal..

[35] Searles K., Chan K. W., Mendes Burak J. A., Zemlyanov D., Safonova O., Copéret C. (2018). Highly Productive Propane Dehydrogenation
Catalyst Using Silica-Supported Ga–Pt Nanoparticles Generated
from Single-Sites. J. Am. Chem. Soc..

[36] Lian K., Yue Y., Basset J.-M., Liu X., Chen L., Ozsoy-Keskinbora C., Bao X., Zhu H. (2022). Surface Organometallic Chemistry as a Versatile Strategy
for Synthesizing Supported Bimetallic Cluster Catalysts. J. Phys. Chem. C.

[37] McCullough K. E., Peczak I. L., Kennedy R. M., Wang Y.-Y., Lin J., Wu X., Paterson A. L., Perras F. A., Hall J., Kropf A. J., Hackler R. A., Shin Y., Niklas J., Poluektov O. G., Wen J., Huang W., Sadow A. D., Poeppelmeier K. R., Delferro M., Ferrandon M. S. (2023). Synthesis of Platinum Nanoparticles
on Strontium Titanate Nanocuboids via Surface Organometallic Grafting
for the Catalytic Hydrogenolysis of Plastic Waste. J. Mater. Chem. A.

[38] Sommer W. J., Weck M. (2007). Supported *N*-Heterocyclic Carbene Complexes in Catalysis. Coord. Chem. Rev..

[39] Ranganath K. V. S., Onitsuka S., Kumar A. K., Inanaga J. (2013). Recent Progress
of
N-Heterocyclic Carbenes in Heterogeneous Catalysis. Catal. Sci. Technol..

[40] Wang W., Cui L., Sun P., Shi L., Yue C., Li F. (2018). Reusable N-Heterocyclic
Carbene Complex Catalysts and Beyond: A Perspective on Recycling Strategies. Chem. Rev..

[41] Culver D. B., Mais M., Kang M.-C., Zhou L., Perras F. A. (2025). Well-Defined
Pt­(0) Heterogeneous Hydrosilylation Catalysts Supported by a Surface
Bound Phosphenium Ligand. Dalton Trans..

[42] Gudat, D. Recent Developments in the Chemistry of N-Heterocyclic Phosphines. In Phosphorus Heterocycles II; Bansal, R. K. , Ed.; Springer: Berlin, Heidelberg, 2010; pp 63–102. 10.1007/7081_2009_5.

[43] Rosenberg L. (2012). Metal Complexes
of Planar PR2 Ligands: Examining the Carbene Analogy. Coord. Chem. Rev..

[44] He M., Hu C., Wei R., Wang X.-F., Liu L. L. (2024). Recent Advances
in the Chemistry of Isolable Carbene Analogues with Group 13–15
Elements. Chem. Soc. Rev..

[45] Zafar M., Subramaniyan V., Tibika F., Tulchinsky Y. (2024). Cationic Ligands
– from Monodentate to Pincer Systems. Chem. Commun..

[46] Culver D. B., Venkatesh A., Huynh W., Rossini A. J., Conley M. P. (2020). Al­(ORF)_3_ (RF = C­(CF_3_)_3_) Activated Silica: A
Well-Defined Weakly Coordinating Surface Anion. Chem. Sci..

[47] Gao J., Perras F. A., Conley M. P. (2025). A Broad-Spectrum Catalyst for Aliphatic
Polymer Breakdown. J. Am. Chem. Soc..

[48] Rodriguez J., Culver D. B., Conley M. P. (2019). Generation of Phosphonium Sites on
Sulfated Zirconium Oxide: Relationship to Brønsted Acid Strength
of Surface – OH Sites. J. Am. Chem. Soc..

[49] Cleary S. R., Starace A. K., Curran-Velasco C. C., Ruddy D. A., McGuirk C. M. (2024). The Overlooked
Potential of Sulfated Zirconia: Reexamining Solid Superacidity Toward
the Controlled Depolymerization of Polyolefins. Langmuir.

[50] Ramirez F., Patwardhan A. V., Kugler H. J., Smith C. P. (1967). Formation
of Phosphorus-Oxygen
Bonds in the Reactions of Triaminophosphines with o-Quinones, Vicinal
Triketones, and Xoxmalonic Esters. Triaminooxyphosphonium Dipolar
Ions and Triaminodioxaphosphoranes. Phosphorus-31 Nuclear Magnetic
Resonance Spectra. J. Am. Chem. Soc..

[51] Abrams M. B., Scott B. L., Baker R. T. (2000). Sterically Tunable Phosphenium Cations:
Synthesis and Characterization of Bis­(Arylamino)­Phosphenium Ions,
Phosphinophosphenium Adducts, and the First Well-Defined Rhodium Phosphenium
Complexes. Organometallics.

[52] Robbie A. J., Cowley A. R., Jones M. W., Dilworth J. R. (2011). Complexes of Sterically-Hindered
Diaminophosphinothiolate Ligands with Rh­(I), Ni­(II) and Pd­(II). Polyhedron.

[53] Rice N. T., Popov I. A., Russo D. R., Bacsa J., Batista E. R., Yang P., Telser J., La Pierre H. S. (2019). Design,
Isolation, and Spectroscopic Analysis of a Tetravalent Terbium Complex. J. Am. Chem. Soc..

[54] Tafazolian H., Culver D. B., Conley M. P. (2017). A Well-Defined
Ni­(II) α-Diimine
Catalyst Supported on Sulfated Zirconia for Polymerization Catalysis. Organometallics.

[55] Culver D. B., Conley M. P. (2018). Activation of C–F
Bonds by Electrophilic Organosilicon
Sites Supported on Sulfated Zirconia. Angew.
Chem., Int. Ed..

[56] Hardman N. J., Abrams M. B., Pribisko M. A., Gilbert T. M., Martin R. L., Kubas G. J., Baker R. T. (2004). Molecular
and Electronic Structure
of Platinum Bis­(N-Arylamino)­Phosphenium Complexes Including [Pt­(Phosphane)­(Phosphenium)­(N-Heterocyclic
Carbene)]. Angew. Chem., Int. Ed..

[57] Karstedt, B. Platinum Complexes of Unsaturated Siloxanes and Platinum Containing Organopolysiloxanes. Patent US3775452A, Nov 27, 1973. https://patents.google.com/patent/US3775452A/en (accessed 2023-11-30).

[58] Rossini A.
J., Hanrahan M. P., Thuo M. (2016). Rapid Acquisition of Wideline MAS
Solid-State NMR Spectra with Fast MAS, Proton Detection, and Dipolar
HMQC Pulse Sequences. Phys. Chem. Chem. Phys..

[59] Venkatesh A., Perras F. A., Rossini A. J. (2021). Proton-Detected Solid-State NMR Spectroscopy
of Spin-1/2 Nuclei with Large Chemical Shift Anisotropy. J. Magn. Reson..

[60] Venkatesh A., Gioffrè D., Atterberry B. A., Rochlitz L., Carnahan S. L., Wang Z., Menzildjian G., Lesage A., Copéret C., Rossini A. J. (2022). Molecular and Electronic Structure of Isolated Platinum
Sites Enabled by the Expedient Measurement of 195Pt Chemical Shift
Anisotropy. J. Am. Chem. Soc..

[61] Markó I. E., Stérin S., Buisine O., Mignani G., Branlard P., Tinant B., Declercq J.-P. (2002). Selective and Efficient Platinum(0)-Carbene
Complexes As Hydrosilylation Catalysts. Science.

[62] Dierick, S. ; Markó, I. E. NHC Platinum(0) Complexes: Unique Catalysts for the Hydrosilylation of Alkenes and Alkynes. In N-Heterocyclic Carbenes; John Wiley & Sons, Ltd, 2014; pp 111–150. 10.1002/9783527671229.ch05.

[63] Berthon-Gelloz G., Schumers J.-M., De Bo G., Markó I. E. (2008). Highly
β-(E)-Selective Hydrosilylation of Terminal and Internal Alkynes
Catalyzed by a (IPr)­Pt­(Diene) Complex. J. Org.
Chem..

[64] Fotie J., Enechojo Agbo M., Qu F., Tolar T. (2020). Dichloro­(Ethylenediamine)­Platinum­(II),
a Water-Soluble Analog of the Antitumor Cisplatin, as a Heterogeneous
Catalyst for a Stereo­selective Hydrosilylation of Alkynes under
Neat Conditions. Tetrahedron Lett..

[65] Jawale D. V., Geertsen V., Miserque F., Berthault P., Gravel E., Doris E. (2021). Solvent-Free Hydrosilylation
of Alkenes
and Alkynes Using Recyclable Platinum on Carbon Nanotubes. Green Chem..

[66] De
Bo G., Berthon-Gelloz G., Tinant B., Markó I. E. (2006). Hydrosilylation
of Alkynes Mediated by N-Heterocyclic Carbene Platinum(0) Complexes. Organometallics.

[67] Dierick S., Vercruysse E., Berthon-Gelloz G., Markó I. E. (2015). User-Friendly
Platinum Catalysts for the Highly Stereo­selective Hydrosilylation
of Alkynes and Alkenes. Chem. – Eur.
J..

[68] Shvydkiy N. V., Rimskiy K. V., Perekalin D. S. (2023). Cyclobutadiene Platinum Complex as
a New Type of Precatalyst for Hydrosilylation of Alkenes and Alkynes. Appl. Organomet. Chem..

[69] Ondar E. E., Kostyukovich A. Y., Burykina J. V., Galushko A. S., Ananikov V. P. (2023). Examination
of Pt_2_dba_3_ as a “Cocktail”-Type
Catalytic System for Alkene and Alkyne Hydrosilylation Reactions. Catal. Sci. Technol..

[70] Chen L., Ali I. S., Sterbinsky G. E., Gamler J. T. L., Skrabalak S. E., Tait S. L. (2019). Alkene Hydrosilylation
on Oxide-Supported Pt-Ligand
Single-Site Catalysts. ChemCatChem.

[71] Jammee R., Kolganov A., Groves M. C., Pidko E. A., Sydora O. L., Conley M. P. (2025). C–H Bond Activation by Sulfated Zirconium Oxide
Is Mediated by a Sulfur-Centered Lewis Superacid. Angew. Chem., Int. Ed..

[72] Ahn H., Nicholas C. P., Marks T. J. (2002). Surface
Organozirconium Electrophiles
Activated by Chemisorption on “Super Acidic” Sulfated
Zirconia as Hydrogenation and Polymerization Catalysts. A Synthetic,
Structural, and Mechanistic Catalytic Study. Organometallics.

[73] Gu W., Stalzer M. M., Nicholas C. P., Bhattacharyya A., Motta A., Gallagher J. R., Zhang G., Miller J. T., Kobayashi T., Pruski M., Delferro M., Marks T. J. (2015). Benzene
Selectivity in Competitive Arene Hydrogenation: Effects of Single-Site
Catalyst···Acidic Oxide Surface Binding Geometry. J. Am. Chem. Soc..

[74] Zhang J., Mason A. H., Wang Y., Motta A., Kobayashi T., Pruski M., Gao Y., Marks T. J. (2021). Beyond the Active
Site. Cp*ZrMe_3_/Sulfated Alumina-Catalyzed Olefin Polymerization
Tacticity via Catalyst···Surface Ion-Pairing. ChemCatChem..

[75] Perras F. A., Paterson A. L., Syed Z. H., Kropf A. J., Kaphan D. M., Delferro M., Pruski M. (2021). Revealing
the Configuration and Conformation
of Surface Organometallic Catalysts with DNP-Enhanced NMR. J. Phys. Chem. C.

[76] Syed Z. H., Mian M. R., Patel R., Xie H., Pengmei Z., Chen Z., Son F. A., Goetjen T. A., Chapovetsky A., Fahy K. M., Sha F., Wang X., Alayoglu S., Kaphan D. M., Chapman K. W., Neurock M., Gagliardi L., Delferro M., Farha O. K. (2022). Sulfated Zirconium
Metal–Organic
Frameworks as Well-Defined Supports for Enhancing Organometallic Catalysis. J. Am. Chem. Soc..

[77] Mazieres M. R., Roques C., Sanchez M., Majoral J. P., Wolf R. (1987). Chlorophospheniums,
Precurseurs de Nouveaux Cations Du Phosphore Dicoordonne. Tetrahedron.

[78] Caputo C. A., Price J. T., Jennings M. C., McDonald R., Jones N. D. (2008). N-Heterocyclic
Phosphenium Cations: Syntheses and Cycloaddition Reactions. Dalton Trans..

