# Supplementary material for: Regioselective Hydrosilylation Catalysis with Supported Well-Defined Pt(0) Complexes: Effects of Surface Anions and Phosphenium Ligands
Source: Inorg Chem. 2026 Feb 16;65(8):4344–50. doi: 10.1021/acs.inorgchem.5c05042 (PMC12958278; doi:10.1021/acs.inorgchem.5c05042)
Supplement: Supplementary file 1 [file ic5c05042_si_001.pdf]

Supplementary Information for

Regioselective hydrosilylation catalysis with supported well-defined Pt(0) complexes: Effects of surface anions and phosphenium ligands

Damien B. Culver,[a,*] Conor Neill,[a] Frédéric A. Perras,[a,b] Mita Halder,[a] and Angela Chartouni[a]

a.) Division of Chemical and Biological Sciences, Ames National Laboratory, Ames, IA 50010, USA

b.) Department of Chemistry, Iowa State University, Ames, IA 50010, USA

*Contact email: culver@ameslab.gov

Table of Contents

General considerations S3
Synthesis and characterization S4
Solid precatalysts S4
Molecular syntheses and precatalyst generation..... S10
Catalytic reactions..... S13
General hydrosilylation reaction setup S13
Hot filtration studies S18
Catalyst reuse study S23
Homogeneous catalysis S24
FTIR and solid-state NMR spectra..... S25
DRIFT spectra S25
SSNMR spectra of **1a-e** S28
SSNMR spectra of **2a-e** S33
SSNMR spectra of **3d** S38
Solution NMR spectra and gas chromatograph S40
Mass balance S40
Molecular syntheses and precatalyst generation..... S42
Catalytic reactions S47
References S48

General considerations

All manipulations were performed in a nitrogen or argon filled inert atmosphere glovebox or on a Schlenk line using standard techniques. Grafting reactions were performed under high vacuum in double Schlenk flasks using standard techniques.[1] Benzene-d_6, dichloromethane-d_2 and chloroform-d were purchased from Cambridge Isotope Laboratories. Benzene-d_6 was dried over sodium and benzophenone, degassed using three freeze, pump, thaw cycles, then distilled under high vacuum and stored over activated 3 Å molecular sieves in an inert atmosphere glovebox. Chloroform-d and dichloromethane-d_2 were dried over CaH_2, degassed using three freeze, pump, thaw cycles, then distilled under high vacuum and stored over activated 3 Å molecular sieves in an inert atmosphere glovebox. Anhydrous solvents for molecular syntheses were purchased from Sigma Aldrich and stored over 3 or 4 Å molecular sieves and degassed prior to use. Toluene for grafting reactions was stored over sodium and benzophenone, and degassed using three freeze, pump, thaw cycles prior to use. Fluorobenzene was dried over CaH_2, degassed using three freeze, pump, thaw cycles, then distilled under high vacuum and stored over activated 3 Å molecular sieves in an inert atmosphere glovebox. 1-octyne were dried over CaH_2, degassed using three freeze, pump, thaw cycles, then distilled under high vacuum and stored over activated 3 Å molecular sieves in an inert atmosphere glovebox. Silanes were dried over 3 Å and degassed prior to use. Diamines were purchased from Ambeed or Sigma Aldrich and utilized without further purification. Ethylenebis(triphenylphosphine)platinum(0) and Platinum(0)-1,3-divinyl-1,1,3,3-tetramethyldisiloxane complex solution (2 wt% Pt in xylene) (Karstedt's catalyst) were purchased from Sigma Aldrich and used without further purification. MeNHPCl,[2] ^{t}BuNHPCl,[3] PhNHPCl,[4] MesNHPCl,[5] DippNHPCl,[5] MeNHPOTf,[6] and PhNHPOTf[7] were synthesized utilizing literature procedures. The solid NHPCls were sublimed under vacuum prior to use. SZO,[8] [^{i}Pr$_3$Si][SZO],[9] and [MesNHP][OTf][5] were synthesized using reported procedures.

Solution NMR spectroscopy was performed at the Iowa State University Chemical Instrumentation Facility on a Varian MR-400, a Bruker Avance NEO 400 MHz system with LN_2-cooled broadband Prodigy Probe, or a Bruker Avance III Spectrometer. Solution ^{1}H NMR spectra were referenced to solvent residual signals. Solution ^{31}P NMR spectra were indirectly referenced to the referenced ^{1}H NMR spectrum. All solution NMR spectra were recorded at 25 °C.

Owing to the supported phosphenium species' high level of motions, cross-polarization is ineffective at room temperature. As such, solid-state ^{13}C, ^{29}Si, and ^{31}P CPMAS NMR spectra were acquired at 100 K using a Bruker AVANCE III 400 MHz NMR spectrometer equipped with a 3.2 mm low-temperature MAS probe. Samples were packed in a glovebox and quickly inserted into the cold nitrogen environment of the probe, which ensures minimal atmosphere exposure. Samples were spun to a frequency of 12 kHz and CPMAS spectra were acquired with a 3 s recycle delay and a 2.5 μs ^{1}H excitation pulse. The ^{13}C, ^{29}Si, and ^{31}P CP contact times lasted 2, 5, and 5 ms. ^{29}Si CPMAS spectra were acquired in 16,384 scans with the exception of the Ph, and Mes-functionalized

phosphenium samples that were instead acquired in 8192 and 2048 scans, respectively. ^{31}P CPMAS spectra were acquired in 512 to 2048 scans. ^{13}C CPMAS spectra were acquired in 2048 to 16,384 scans.

^{1}H NMR spectra were acquired using fast-MAS (37 kHz) with a 600 MHz Bruker AVANCE III NMR spectrometer equipped with a Varian 1.6 mm fast-MAS probe. Samples were ground into perdeuterated eicosane to prevent degradation in a glovebox.[10] Spectra were acquired in 16 scans with a 2 s recycle delay using a Bloch decay sequence. DQ/SQ correlation spectra were also acquired using the $R12_2^5$ recoupling sequence[11] with a total of 432 μs of dipolar recoupling in 16 scans and 128 t_1 increments. The States method was used for sign discretion.

Diffuse reflectance infrared Fourier transform infrared (DRIFTS) spectra were measured on a Bruker ALPHA II spectrometer contained within an argon filled glovebox and spectra were recorded within the 4000-400 cm^{-1} range. Elemental analyses were performed on an Agilent 5800 ICP-OES spectrometer. To perform the analysis, 10-12 mg of the samples were soaked in 1 mL hydrofluoric acid (46-51% assay) and 1 mL aqua regia (trace metal grade hydrochloric acid (assay 34-37% w/v) and nitric acid (70% v/v)), for overnight, followed by dilution to 14 mL with DI water.

Synthesis and characterization

Solid precatalysts

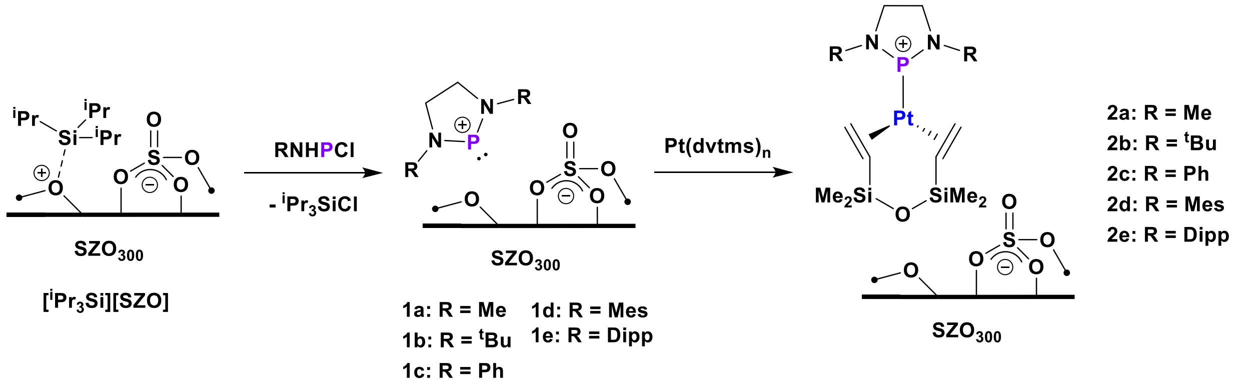

[RNHP][SZO] (**1a-e**): In a double Schlenk flask, [$^{i}Pr_3Si$][SZO] (1.0 g, 0.13 mmol of $^{i}Pr_3Si$) and N-heterocyclic phosphine chloride (0.13 mmol) were combined on one side of the flask along with small stir bar in an argon filled glovebox. The flask was sealed, removed from the glovebox, and connected to a high vacuum line. Under high vacuum, toluene (5 mL) was condensed over the solids at -196 °C. The flask was sealed under static vacuum, and the solution was allowed to warm up to room temperature while stirring (100 rpm). The reaction was stirred at room temperature for one hour. The solution was filtered to the opposite side of the double Schlenk. The toluene was vacuum distilled back over the solid at -196 °C, warmed to room temperature, and stirred for 2 minutes, followed by filtration to wash the solid. The solid was washed three more times using this technique. After the final wash, the solid was dried under high vacuum for 1 hour, resulting in white-

red-orange colored solids **1a-e**. Note that in the synthesis of **1a**, MeNHPCl is a liquid, therefore was placed in the opposite arm of the double Schlenk, dissolved in the toluene, then the solution was transferred through the frit onto the [$^{i}Pr_3Si$][SZO] then the remainder of the procedure was identical. **Table S1**-**S3** contain summaries of the characterization data for **1a-e**. The FTIR and solid-state NMR spectra section contains DRIFTS and SSNMR spectra of **1a-e**. **1a-e** contain small impurities of oxidized P compound that can be observed in the ^{31}P SSNMR spectra near 0 ppm. Oxidation likely occurs during the grafting process as a result of a small concentration of pyrosulfates on the surface of SZO.[12]

The formation of $^{i}Pr_3SiCl$ was confirmed by performing the reaction on a 25-30 mg scale of [$^{i}Pr_3Si$][SZO] in a PTFE – valved NMR tube and monitoring the reaction by ^{1}H NMR spectroscopy in the presence of hexamethylbenzene as an internal standard. After 1 h, 2-3 drops of pyridine was added to the NMR tube to displace physisorbed $^{i}Pr_3SiCl$. The results are summarized in **Table S1**. Representative NMR spectra of the NMR tube reactions are provided in **Figure S25**.

[(NHP)Pt(dvtms)][SZO] (**2a-e**): **1a-1e** (0.5 g, 34-68 μmol P) was added to a Schlenk bomb along with a small stir bar. The flask was sealed, removed from the glovebox, and connected to a high vacuum line. Under high vacuum, toluene (3 mL) was condensed over solid **1a-1e** at -196 °C. In a Schlenk flask, Karstedt's catalyst solution (2 wt% Pt in xylene, 585 mg, 60 μmol) was diluted with toluene (2 mL). The slurry of **1** in toluene was cooled to 0 ◦C and the diluted Karstedt's catalyst solution was added dropwise via cannula under argon flow while stirring (50 rpm). The flask was sealed under argon and the reaction was allowed to warm up to RT and stirred for 2 hours. After the reaction, the solution was removed via decantation with a cannula under argon flow. Then, fresh toluene (5 mL) was vacuum distilled over the solid at -196 °C, warmed to room temperature, and stirred for 2 minutes followed by decantation to wash the solid. The solid was washed two more times using this technique. After the final wash, the solids was dried under high vacuum for 1 hour resulting in yellow to orange solid **2a-e**. **Table S1**-**S3** contain summaries of the characterization data for **2a-e**. The FTIR and solid-state NMR spectra section contains DRIFTS and SSNMR spectra of **2a-e**.

A note on [$(NHP)_2Pt$][SZO] formation: A ^{31}P DQ SSNMR experiment could determine if [$(NHP)_2Pt$][SZO] sites form, however the ^{1}H-^{31}P cross polarization was unsuccessful, therefore this experiment is also unlikely to be successful.[13] We evaluated the potential for [$(NHP)_2Pt$][SZO] with molecular analogues by testing the reaction of excess NHPX ligands with Karstedt's catalyst in the *Molecular syntheses and precatalyst generation* section.

Surface 1,3-Divinyltetramethyldisiloxane (dvtms) quantification: A PTFE-valved NMR tube was charged with **2a-2e** (25 mg, 0.9 – 2.0 μmol Pt), PPh_3 (10 mg, 38 μmol) and hexamethylbenzene (internal standard). The solids were dissolved or suspended in benzene-d_6 (0.5 mL) at room temperature and allowed to react overnight. The liberated

dvtms was quantified by solution ^1H NMR against the internal standard (see **Figure S26** for a representative spectrum). The results are shown in **Table S1**.

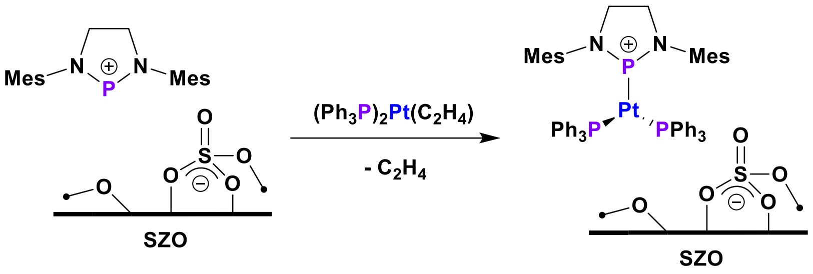

1d 3d

[(MesNHP)Pt(PPh3)2][SZO] (**3d**): **1d** (0.5 g, 49 μmol P) and ethylene bis(triphenylphosphine)platinum(0) (44 mg, 59 μmol) were added to a double Schlenk flask along with a small stir bar. The flask was sealed, removed from the glovebox, and connected to a high vacuum line. Under high vacuum, toluene (5 mL) was condensed over the solids at -196 °C. The flask was sealed under vacuum and the reaction was allowed to warm up to RT and stirred for 30 min during which effervescence was observed. The solution was filtered to the opposite side of the double Schlenk. The toluene was vacuum distilled back over the solid at -196 °C, warmed to room temperature, and stirred for 2 minutes, followed by filtration to wash the solid. The solid was washed three more times using this technique. After the final wash, the solid was dried under high vacuum for 1 hour, resulting in yellow solid **3d**. **Table S1**-**S3** contain summaries of the characterization data for **3d**. The FTIR and solid-state NMR spectra section for spectra of **3d**.

Surface PPh_3 quantification: In a NMR tube, **3d** (25 mg, 1.1 μmol Pt) was combined with hexamethylbenzene (internal standard) and suspended in C_6D_6 (0.5 mL). At room temperature, PMe_3 (0.05 mL, 0.5 mmol) was added and the solid turned pumpkin orange immediately. The reaction was allowed to react overnight at room temperature. The PPh_3 released was quantified by solution ^1H NMR against the internal standard (see **Figure S27** for a representative spectrum). The average of two experiments was 0.10 (±0.01) mmol/g of PPh_3.

Table S1. Summary of the elemental analyses and mass balance data for **1a-f** and **2a-f**.[a]

Material	Color	Wt%[b]		mmol/g[b]		iPr$_3$SiCl (mmol/g)[c]	dvtms (mmol/g)[d]	P:Pt mol ratio	dvtms:Pt mol ratio
		P	Pt	P	Pt				
**1a**	White	0.42 (0.03)	---	0.136 (0.008)	---	0.1221 (0.0003)	---	---	---
**1b**	White	0.39 (0.01)	---	0.126 (0.003)	---	0.122 (0.003)	---	---	---
**1c**	Red-orange	0.31 (0.01)	---	0.100 (0.003)	---	0.113 (0.002)	---	---	---
**1d**	Peep yellow	0.30 (0.01)	---	0.098 (0.002)	---	0.107 (0.009)	---	---	---
**1e**	Light yellow	0.21 (0.01)	---	0.067 (0.002)	---	0.0675 (0.0006)	---	---	---
**2a**	Highlighter orange	0.37 (0.02)	1.59 (0.09)	0.118 (0.005)	0.081 (0.004)	---	0.069 (0.001)	1.5 (0.1)	0.9 (0.1)
**2b**	Light yellow	0.364 (0.001)	1.190 (0.003)	0.117 (0.0002)	0.0610 (0.0002)	---	0.035 (0.002)	1.92 (0.01)	0.57 (0.03)
**2c**	Orange	0.22 (0.01)	1.07 (0.05)	0.072 (0.003)	0.055 (0.002)	---	0.0518 (0.0006)	1.3 (0.1)	0.94 (0.05)
**2d**	Peep yellow	0.22 (0.01)	1.224 (0.001)	0.072 (0.002)	0.0627 (0.0001)	---	0.066 (0.002)	1.15 (0.03)	1.05 (0.03)
**2e**	Light yellow	0.11 (0.01)	0.74(0.06)	0.036 (0.003)	0.038 (0.003)	---	0.0497 (0.0005)	0.9 (0.2)	1.3 (0.1)[e]
**3d**	Bright yellow	0.442 (0.007)	0.88 (0.04)	0.143 (0.002)	0.045 (0.002)	---	0.10 (0.01) (PPh_3)	3.2 (0.2)	2.2 (0.3) (PPh_3)

a. Average of 2 runs. Errors are indicated in parentheses. --- indicate not applicable. b. Determined by ICP-OES, see **General considerations** for details. c. Determined by NMR scale grafting reactions in benzene-d_6 over 1 h followed by addition of pyridine to desorb physisorbed $^{i}Pr_3SiCl$ then quantified by ^{1}H NMR spectroscopy in the presence of hexamethylbenzene as an internal standard. d. Determined by suspending **2a-f** in benzene-d_6 and reacting the solids with PPh_3 or PMe_3 in the presence of hexamethylbenzene as an internal standard then determining the dvtms by ^{1}H NMR spectroscopy. e. The slightly elevated ratio in **2e** is likely due to physisorbed dvtms.

Table S2. Summary of ^{31}P SSNMR data.[a]

Sample	δ_{iso} (ppm)	Ω (ppm)	κ	Notes
**1a**	269	460	-1.0	Sharp. Minor impurity
**1b**	271	520	-0.7	Sharp.
**1c**	252	540	-0.8	Sharp. Minor impurity
**1d**	277	580	-0.6	Sharp.
**1e**	265	498	-0.6	Sharp.
**2a**	292	~460	~-1	Broad and dynamic. Minor impurity close to 0 ppm.
**2b**	288	530	-1.0	Broad
**2c**	292	>400	~-1	Broad and dynamic. Minor impurity close to 0 ppm.
**2d**	283	520	-0.8	Broad
**2e**	277	540	-0.7	Broad
**3d**	295<br>46 (PPh_3)	527<br>ND	-0.5<br>ND	$^{1}J_{Pt\text{-}P}$ = 5.4 kHz (NHP)

a. Recorded at 100 K while spinning at 12 kHz, see **General considerations** for more details. ND = not determined.

Table S3. Summary of the ^{1}H and ^{13}C SSNMR chemical shifts (ppm) and assignments.[a]

**Material**	**^{1}H[b]**	**^{1}H assignment**	**^{13}C[c]**	**^{13}C assignment**	**^{29}Si** (dvtms)[c]
**1a**	2.8<br>3.2	$NC\mathbf{H}_3$<br>$NC\mathbf{H}_2$	33<br>55	$N\mathbf{C}H_3$<br>$N\mathbf{C}H_2$	NA
**1b**	1.4<br>4.0 (B)	$NC(C\mathbf{H}_3)_3$<br>$NC\mathbf{H}_2$	29<br>50<br>58	$NC(\mathbf{C}H_3)_3$<br>$N\mathbf{C}(CH_3)_3$<br>$NC\mathbf{H}_2$	NA
**1c**	4.7 (B)<br>7.2	$NC\mathbf{H}_2$<br>$NC_6\mathbf{H}_5$	50<br>108-136	$N\mathbf{C}H_2$<br>$N\mathbf{C}_6H_5$	NA
**1d**	2.3<br>4.7 (B)<br>6.9	$NC_6H_2(C\mathbf{H}_3)_3$<br>$NC\mathbf{H}_2$<br>$NC_6\mathbf{H}_2(CH_3)_3$	17<br>56<br>124-142	$NC_6H_2(\mathbf{C}H_3)_3$<br>$N\mathbf{C}H_2$<br>$N\mathbf{C}_6H_2(CH_3)_3$	NA
**1e**	1.3<br>3.2<br>4.8 (B)<br>7.3	$NC_6H_3(CH(C\mathbf{H}_3)_2)_2$<br>$NC_6H_3(C\mathbf{H}(CH_3)_2)_2$<br>$NC\mathbf{H}_2$<br>$NC_6\mathbf{H}_3(CH(CH_3)_2)_2$	28<br>59<br>125-130<br>146	$NC_6H_3(\mathbf{C}H(\mathbf{C}H_3)_2)_2$<br>$N\mathbf{C}H_2$<br>$N\mathbf{C}_6H_3(CH(CH_3)_2)_2$<br>$N\mathbf{C}_6H_3(CH(CH_3)_2)_2$	NA
**2a**	-0.1<br>2.8 (B)	$Si(C\mathbf{H}_3)_2$<br>$NC\mathbf{H}_3$ + $NC\mathbf{H}_2$ + $SiC\mathbf{H}C\mathbf{H}_2$	-1<br>32<br>50<br>68	$Si(\mathbf{C}H_3)_2$<br>$N\mathbf{C}H_3$<br>$N\mathbf{C}H_2$<br>$Si\mathbf{C}H\mathbf{C}H_2$	3
**2b**	0.0<br>1.3<br>3.4 (B)	$Si(C\mathbf{H}_3)_2$<br>$NC(C\mathbf{H}_3)_3$<br>$NC\mathbf{H}_2$ + $SiC\mathbf{H}C\mathbf{H}_2$	-1<br>29<br>38-72	$Si(\mathbf{C}H_3)_2$<br>$NC(\mathbf{C}H_3)_3$<br>$N\mathbf{C}(CH_3)_3$ + $N\mathbf{C}H_2$ + $Si\mathbf{C}H\mathbf{C}H_2$	3
**2c**	-0.1<br>3.3 (B)<br>7.1	$Si(C\mathbf{H}_3)_2$<br>$NC\mathbf{H}_2$ + $SiC\mathbf{H}C\mathbf{H}_2$<br>$NC_6\mathbf{H}_5$	-1<br>50<br>68<br>111-143	$Si(\mathbf{C}H_3)_2$<br>$N\mathbf{C}H_2$<br>$Si\mathbf{C}H\mathbf{C}H_2$<br>$N\mathbf{C}_6H_5$	3
**2d**	-0.7<br>0.0<br>2.1<br>4.3 (B)<br>6.7	$Si(C\mathbf{H}_3)_2$<br>$Si(C\mathbf{H}_3)_2$<br>$NC_6H_2(C\mathbf{H}_3)_3$<br>$NC\mathbf{H}_2$ + $SiC\mathbf{H}C\mathbf{H}_2$<br>$NC_6\mathbf{H}_2(CH_3)_3$	-2<br>17<br>52<br>52-74<br>122-143	$Si(\mathbf{C}H_3)_2$<br>$NC_6H_2(\mathbf{C}H_3)_3$<br>$N\mathbf{C}H_2$<br>$Si\mathbf{C}H\mathbf{C}H_2$<br>$N\mathbf{C}_6H_2(CH_3)_3$	3
**2e**	-0.7<br>0.0<br>1<br>2.7<br>4.5 (B)<br>7.1	$Si(C\mathbf{H}_3)_2$<br>$Si(C\mathbf{H}_3)_2$<br>$NC_6H_3(CH(C\mathbf{H}_3)_2)_2$<br>$NC_6H_3(C\mathbf{H}(CH_3)_2)_2$<br>$NC\mathbf{H}_2$ + $SiC\mathbf{H}C\mathbf{H}_2$<br>$NC_6\mathbf{H}_3(CH(CH_3)_2)_2$	-2<br>28<br>50-76<br>118-135<br>145	$Si(\mathbf{C}H_3)_2$<br>$NC_6H_3(\mathbf{C}H(\mathbf{C}H_3)_2)_2$<br>$N\mathbf{C}H_2$ + $Si\mathbf{C}H\mathbf{C}H_2$<br>$N\mathbf{C}_6H_3(CH(CH_3)_2)_2$<br>$N\mathbf{C}_6H_3(CH(CH_3)_2)_2$	2
**3e**	2.3<br>4 (B)<br>7.1	$NC_6H_2(C\mathbf{H}_3)_3$<br>$NC\mathbf{H}_2$<br>$NC_6\mathbf{H}_2(CH_3)_3$ + $P(C_6\mathbf{H}_5)_3$	20<br>53<br>132	$NC_6H_2(\mathbf{C}H_3)_3$<br>$N\mathbf{C}H_2$<br>$N\mathbf{C}_6H_2(CH_3)_3$ + $P(\mathbf{C}_6H_5)_3$	NA

a. See **General considerations** for detailed conditions. Signals for residual $^{i}Pr_3Si$- sites and bulk SiO_2 signals are not included.[9] B = broad. NA = not applicable. b. Recorded at room temperature while spinning at 37.037 kHz. c. Recorded at 100 K while spinning at 12 kHz. Overlapping and broad signals in the ^{13}C NMR spectra are provided as ranges. Only the Pt(dvtms) ^{29}Si NMR signal is provided in this table.

Molecular syntheses and precatalyst generation

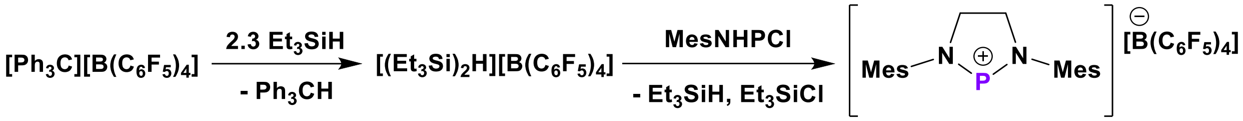

[MesNHP][B(C_6F_5)$_4$]: [(Et_3Si)$_2$H][B(C_6F_5)$_4$] was generated following the published procedure.[14] In a vial in a N_2 filled glovebox, [Ph_3C][B(C_6F_5)$_4$] (0.50 g, 0.54 mmol) was suspended in toluene (8 mL) then Et_3SiH (0.20 mL, 1.26 mmol) was added while stirring at room temperature. The reaction turned light yellow with a brown solid and was stirred for 2 days. MesNHPCl (195 mg, 0.54 mmol) was dissolved in toluene (8 mL) then added dropwise to the mixture at room temperature resulting in cloudy pale-yellow solution that was stirred for 1 h. The toluene was partially removed *en vacuo* to 10 mL then pentane (10 mL) was added while stirring resulted in more solid precipitation. The solid was isolated by filtration and washed with pentane (3 X 10 mL) and dried *en vacuo* for 30 min resulting 520 mg (61 %) of pale-yellow solid [MesNHP][B(C_6F_5)$_4$]. The NMR spectroscopy are comparable to the previous literature report for [MesNHP][B(C_6H_3(CF_3)$_2$]].[5] ^{1}H NMR (chloroform-*d*, 400 MHz): 7.09 (s, 4H, Ar**H**), 4.38 (d, 4H, $^3J_{P\text{-}H}$ = 4.3 Hz, C**H**$_2$), 2.37 (s, 18H, Ar**Me**); ^{31}P{^{1}H) NMR (chloroform-*d*, 162 MHz): 276.4 ppm. Images of the spectra are provided in **Figure S28** and **S25**.

Scheme S1. Possible coordination complexes from reactions between molecular NHPX ligands and Karstedt's catalyst. X^- = OTf^- or B(C_6F_5)$_4^-$.

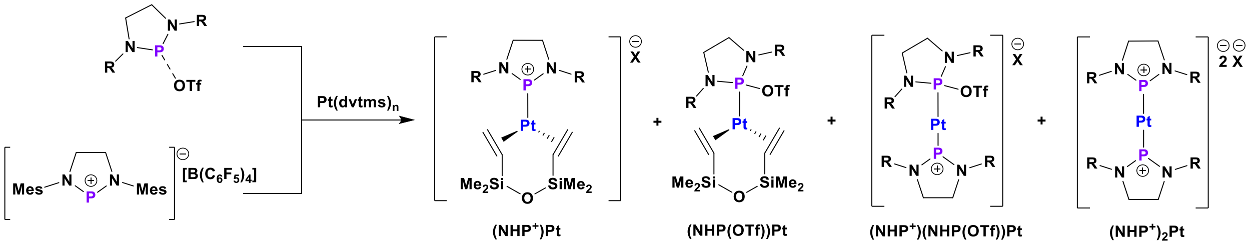

NHPOTf or [NHP][B(C_6F_5)$_4$] + Karstedt's catalyst: In a PTFE-valved NMR tube, NHPOTf or [NHP][B(C_6F_5)$_4$] (12 μmol) were combined with Karstedt's catalyst (0.10 g, 10. umol Pt) in dichloromethane-*d$_2$* (0.5 mL). The reactions were shaken then analyzed by solution ^{19}F and ^{31}P NMR spectroscopy. After analyses, another 12 μmol of ligand was added to each NMR mixture. The major product signals are provided below with structure assignments and images of the spectra are provided in the **Solution NMR spectra and gas chromatograph** section.

Bulk-scale attempts to isolate products from the MesNHPOTf/Karstedt's catalyst reaction led to decomposition into a dark oil upon removal of volatiles under vacuum. The resulting material differed from the *in-situ* species observed in solution and lacked any detectable [NHP-Pt]$^+$ complexes.

MeNHPOTf (1.2 equiv.): Color: amber. $^{19}F\{^1H\}$ NMR (dichloromethane-d_2, 565 MHz): -78.2 (bs); $^{31}P\{^1H)$ NMR (dichloromethane-d_2, 162 MHz): 221.8 (s/d, $^1J_{PtP}$ = 3478 Hz, unidentified P-Pt species, minor), 160.6 (s/d, $^1J_{PtP}$ = 5664 Hz, NH**P**(OTf))Pt, major), 137.1 (s/d, $^1J_{PtP}$ = 5782 Hz, (NH**P**(OTf))Pt, major), and 137.0 (s/d, $^1J_{PtP}$ = 5790 Hz, (NH**P**(OTf))Pt, major) ppm. The major species have similar $^{31}P\{^1H)$ chemical shifts upfield of MeNHPOTf (264 ppm)[6] and $^1J_{PtP}$ suggesting the triflate is interacting with the NHP^+ and they have similar structures, likely isomers of (NHP(OTf))Pt(dvtms) with different binding modes for the triflate (O-P or bridging the P-Pt) or the dvtms ligand. The minor species at 221.8 ppm has a smaller $^1J_{PtP}$ than is typical for a phosphenium-Pt(0) coupling, however the high chemical shift suggests the P is still cationic; more study is necessary to determine the structure.

MeNHPOTf (2.4 equiv.): Color: dark amber. $^{31}P\{^1H)$ NMR (dichloromethane-d_2, 162 MHz) contains a mixture of multiple minor signals and broad signals, including a very broad signal at ~310 ppm that may be attributed to a $(NHP^+)_nPt$ (n = 2 or 3) or $[(NHP^+)Pt]_n$ species, but requires more study to confirm.

PhNHPOTf (1.2 equiv.): Color: dark amber. $^{19}F\{^1H\}$ NMR (dichloromethane-d_2, 565 MHz): -77.8 (bs); $^{31}P\{^1H)$ NMR (dichloromethane-d_2, 162 MHz): 205.0 (s/d, $^1J_{PtP}$ = 3626 Hz, unidentified P-Pt species, minor product), and 106.1 (s/d, $^1J_{PtP}$ = 5562 Hz, (NH**P**(OTf))Pt, major product) ppm.

PhNHPOTf (2.4 equiv.): Color: dark red-brown. $^{31}P\{^1H)$ NMR (dichloromethane-d_2, 162 MHz) contains a mixture of multiple minor signals and broad signals, including very broad signals at ~240 and 110 ppm that may be attributed to a (NHP^+)(NHP(OTf))Pt species, but requires more study to confirm.

MesNHPOTf (1.2 equiv.): Color: pale yellow. $^{19}F\{^1H\}$ NMR (dichloromethane-d_2, 565 MHz): -78.4 (bs); $^{31}P\{^1H)$ NMR (dichloromethane-d_2, 162 MHz): 282.4 (s/d, $^1J_{PtP}$ = 5888 Hz, (NH**P**$^+$)Pt, major product), and 112.3 (s/d, $^1J_{PtP}$ = 4756 Hz, (NH**P**(OTf))Pt, minor product) ppm. The proposed structures of the two major species are shown in the scheme above. The chemical shift and large $^1J_{PtP}$ for the signal at 282.4 ppm (major species) suggests the complex is $[(NHP^+)Pt(dvtms)][OTf]$. The minor species at 112.3 ppm is assigned to (NHP(OTf))Pt(dvtms), where the OTf^- is bound to the P, resulting in the low chemical shift but large $^1J_{PtP}$. This result is similar to the observation we made using DippNHPOTf in a previous paper.[15]

MesNHPOTf (2.4 equiv.): Color: yellow. $^{19}F\{^1H\}$ NMR (dichloromethane-d_2, 565 MHz): -78.5 (s); $^{31}P\{^1H)$ NMR (dichloromethane-d_2, 162 MHz): 284.7 (d/dd, $^1J_{PtP}$ = 7076 Hz, $^2J_{PP}$ = 200. Hz, (NH**P**$^+$)(NHP(OTf))Pt, minor), 282.4 (s/d, $^1J_{PtP}$ = 5885 Hz, (NH**P**$^+$)Pt, major), and 119.0 (d/dd, $^1J_{PtP}$ = 5400 Hz, $^2J_{PP}$ = 200. Hz, (NHP^+)(NH**P**(OTf))Pt, minor) ppm. The chemical shift and large $^1J_{PtP}$ for the signal at 282.4 ppm (major species) suggests the complex is $[(NHP^+)Pt(dvtms)][OTf]$, identical to the major species in the presence of 1.2 equiv. The minor species, with signals at 284.7 and 119 ppm and assigned to (NHP^+)(NHP(OTf))Pt, also exhibits large $^1J_{PtP}$ indicating both P are phosphenium ligands

bound to Pt and the large $^2J_{PP}$ suggests the ligands are *trans* to one another, with the P with the signal at 119 ppm maintaining triflate coordination to the P and the other triflate outer sphere or interacting with Pt. The reaction also forms significant non-Pt containing products, as evidenced by the singlets at 28 and 9 ppm in the $^{31}P\{^1H)$ NMR spectrum, potentially from unknown side reactions between the ligand and the excess dvtms.

[MesNHP][$B(C_6F_5)_4$] (1.2 equiv.): Color: pale yellow. $^{31}P\{^1H)$ NMR (dichloromethane-*d*$_2$, 162 MHz): 282.1 (s/d, $^1J_{PtP}$ = 5960 Hz, (NH**P**$^+$)Pt) ppm. The chemical shift and large $^1J_{PtP}$ suggests the complex is [(NHP$^+$)Pt(dvtms)][$B(C_6F_5)_4$].

[MesNHP][$B(C_6F_5)_4$] (2.4 equiv.): Color: pale yellow. No new P-Pt species after 2 h at rt.

Catalytic reactions

General hydrosilylation reaction setup

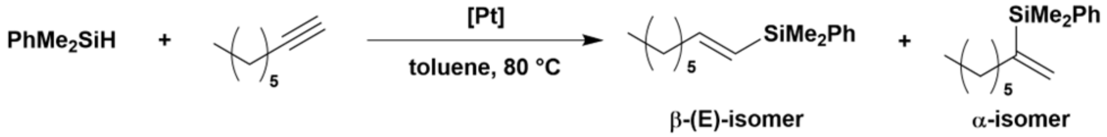

In an argon filled glovebox, precatalyst (5.0 – 25 mg) was weighted out in a 20 mL glass vial and a small stir bar was added to the vial. The vial was transferred to a nitrogen filled glovebox, then a solution (10-16.2 mL) containing 1-octyne (0.5 M), $PhMe_2SiH$ (0.5 M), and internal standard of cyclooctane in toluene was added to the vial via syringe. The vial was sealed and placed in a heating block preheated to 80 °C. The reactions were stirred at 500 rpm for the indicated time in **Table 1** of the main text. Reaction progress was determined by taking small aliquots (<50 μL) of the reactions and analyzing the aliquots GC-FID after dilution with cyclohexane (see **General considerations** for further details). The reaction progression data is provided in **Figure S1** and **Figure S2**, and **Table S4** and **Table S5**. A representative gas chromatogram is provided in **Figure S38**.

Entries 2, 6, and 8 of **Table 1** in the main text involving the 0.01 mol% Pt loading of **2b**, **2d**, and **2e** were performed in a similar manner and were checked after 1 hour by taking an aliquot and were determined to be ~90 % complete by ^1H NMR spectroscopy. The reactions were allowed to progress for a total of 2 hours to reach >99 % silane consumption. Entry 5 of **Table 1** of the main text was performed analogously with **3d** (5.0 mg, 0.32 μmol Pt) and 1.3 mL of the stock solution (0.65 mmol of substrate) in a 1-dram vial for 30 minutes.

Entry 10 of **Table 1** of the main text involving the 0.45 mol% Pt loading of **3d** (25 mg, 1.1 μmol of Pt) was performed in a PTFE-valved NMR tube with 0.5 mL of a stock solution of 0.5 M 1-octyne, $PhMe_2SiH$ and cyclooctane (0.15 M) as an internal standard. The reaction was heated at 80 °C for 21 h. The reaction was monitored by cooling the reaction to room temperature then analyzing by ^1H NMR spectroscopy. The spectra for one of the duplicate runs are provided in **Figure S37** and the data is summarized in **Figure S2** and **Table S5**. The signal/noise in the olefin region was inadequate to obtain accurate integrals for the α-product, therefore the regioselectivity was determined at the end of the 21 hours by NMR spectroscopy and cross-checked with GC-FID.

Chemoselectivity notes: The high Pt loading reactions generally produce <90 % yield of products. This may be due to physisorption of products/silane that are not counted bringing the yield down and increasing consumption, or formation of byproducts common in Pt catalyzed hydrosilylation. Hydrosilylation catalysts are known to couple silanes to form R_3Si-SiR_3 and hydrogen, which then can hydrogenate the octyne to octenes. Close inspection of the NMR spectra from the 0.45 mol% Pt reaction with **3d** shows the presence of Me_2PhSi-$SiPhMe_2$ which contains a small signal at 0.36 ppm matching previous literature. Some silane may also react with residual hydroxyls and pyrosulfates

on the surface of the precatalysts, further reducing the chemoselectivities at higher precatalyst loadings.

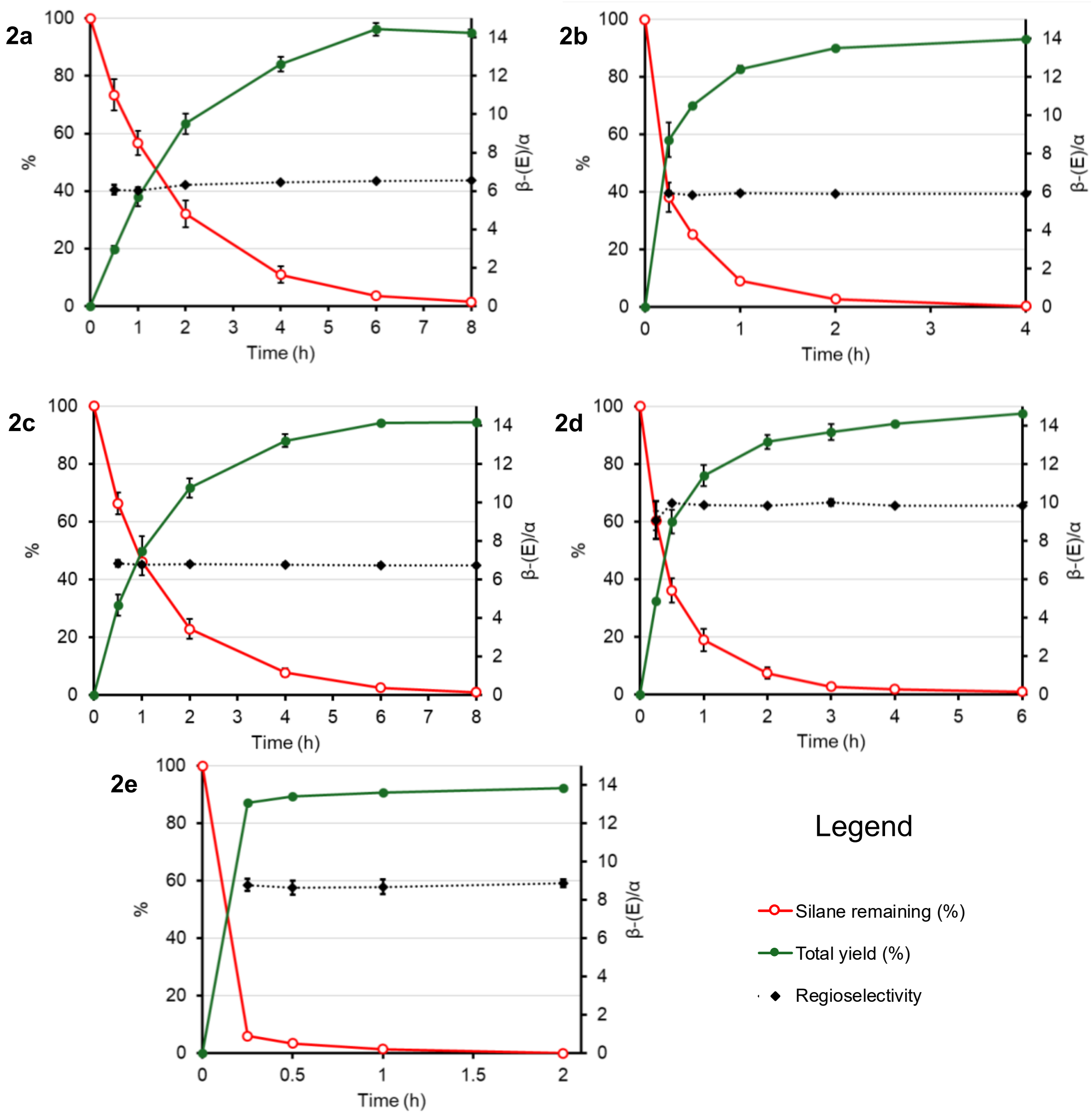

Figure S1. Reaction progression plots for **2a-e** (0.005 mol% Pt) catalyzed hydrosilylation of 1-octyne with Me_2PhSiH. The catalyst associated with each plot is on the top left corner of each panel. Values are provided in **Table S4**.

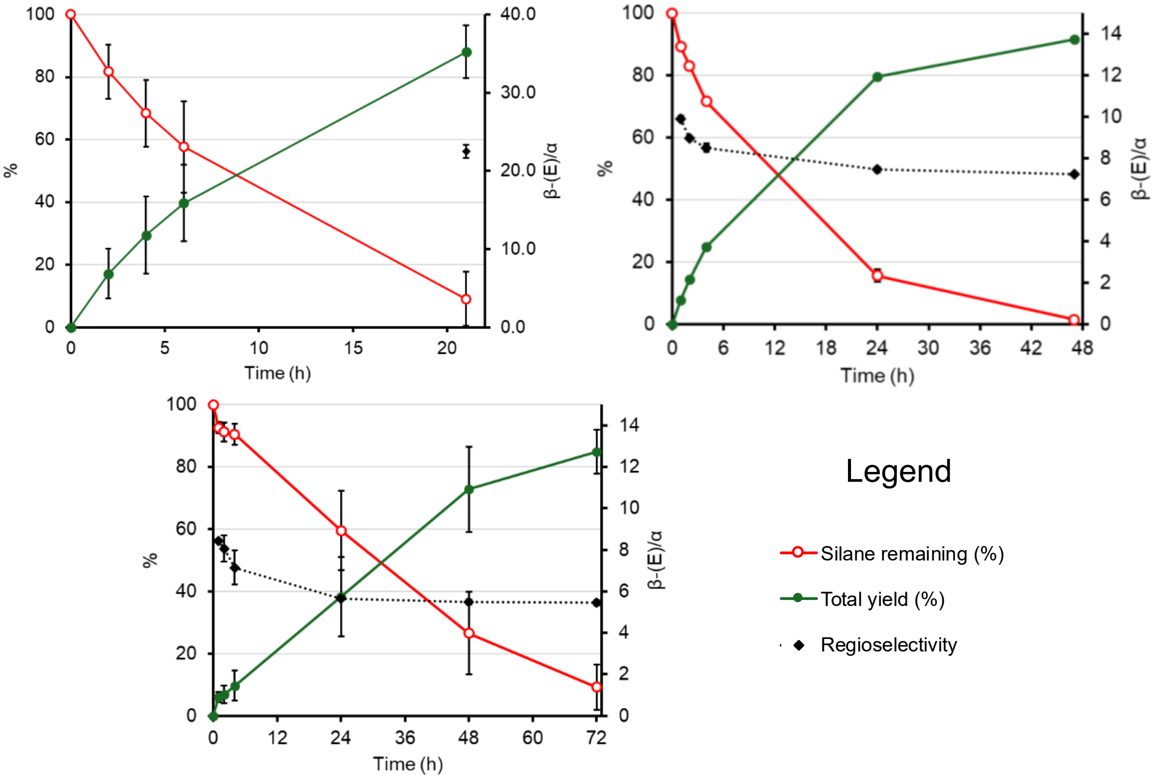

Figure S2. Reaction progression plots for **3d** catalyzed hydrosilylation of 1-octyne with Me_2PhSiH at 0.45 mol% (top left), 0.045 mol% (top right), and 0.0045 mol% (bottom left) Pt loadings. The legend is in the panel on the bottom right. The catalyst associated with each plot is on the top left corner of each panel. For 0.45 mol% Pt the reactions were monitored via 1H NMR spectroscopy and the final results were determined by GC-FID. Values are provided in **Table S5**.

Table S4. Summary of the reaction progression data for **2a-e** catalyzed hydrosilylation of 1-octyne with Me_2PhSiH at 0.005 mol% Pt.[a]

Time (h)	Silane remaining (%)	Error	Total yield (%)	Error	Regioselectivity (β-(E)/α)	Error	TOF (min^{-1})	Error
**2a**								
0	100	NA	0	NA	NA	NA	NA	NA
0.5	73	5	20	1	6.1	0.3	131	8
1	57	4	38	3	6.01	0.09	127	11
2	32	5	63	3	6.32	0.02	106	6
4	11	3	84	3	6.46	0.06	70	2
6	3	1	96	2	6.52	0.04	54	1
8	1.4	0.5	95	1	6.54	0.03	39.6	0.5
**2b**								
0	100	NA	0	NA	NA	NA	NA	NA
0.25	38	5	58	6	5.9	0.1	775	80.
0.5	25.4	0.2	70.	1	5.85	0.02	466	4
1	9	1	83	1	5.95	0.09	276	4
2	2.8	0.3	90.0	0.1	5.92	0.03	150.0	0.1
4	0.4	0.1	93.2	0.4	5.92	0.03	77.7	0.3
**2c**								
0	100	NA	0	NA	NA	NA	NA	NA
0.5	66	4	31	4	6.8	0.2	207	25
1	46	5	50.	5	6.77	0.09	166	17
2	23	3	72	3	6.80	0.08	119	6
4	8	1	88	2	6.78	0.08	73	2
6	3	1	94.2	0.5	6.74	0.08	52.3	0.3
8	1.0	0.3	94.5	0.3	6.72	0.07	39.4	0.1
**2d**								
0	100	NA	0	NA	NA	NA	NA	NA
0.25	60.	3	32.5	0.7	9	1	433	9
0.5	36	4	60.	4	9.95	0.05	399	28
1	19	4	76	4	9.86	0.01	253	12
2	7	2	88	2	9.83	0.02	146	4
3	3	1	91	3	10.0	0.2	101	3
4	2	1	93.9	0.5	9.83	0.01	78.2	0.4
6	0.9	0.1	97.4	0.9	9.83	0.01	54.1	0.4
**2e**								
0	100	NA	0	NA	NA	NA	NA	NA
0.25	6.1	0.7	87.1	0.4	8.8	0.3	1161	6
0.5	3.5	0.1	89.4	0.2	8.6	0.4	596	2
1	1.5	0.1	90.7	0.1	8.7	0.4	302	0.1
2	0.0	0.0	92.4	0.3	8.9	0.2	154	0.5

a. All reactions performed in duplicate and the numbers provided are averages with the error provided in the column immediately to the right for each value. See the *General hydrosilylation reaction setup* section for details.

Table S5. Summary of reaction progression data for **3d** catalyzed hydrosilylation of 1-octyne with Me_2PhSiH at 0.45 mol%, 0.045 mol%, and 0.0045 mol% Pt loadings.[a]

Time (h)	Silane remaining (%)	Error	Total yield (%)	Error	Regioselectivity (β-(E)/α)	Error	TOF (min^{-1})	Error
**0.45 mol% Pt[b]**								
0	100	NA	0	NA	NA	NA	NA	NA
2	82	9	17	8	ND[c]	NA	0.3	0.1
4	68	11	29	12	ND[c]	NA	0.3	0.1
6	58	15	40	12	ND[c]	NA	0.25	0.08
21	13	11	86	11	24	1	0.15	0.02
21[d]	9	9	88	9	22.5	0.9	0.16	0.01
**0.045 mol% Pt**								
0	100	NA	0	NA	NA	NA	NA	NA
1	89.2	0.2	7.9	0.4	9.9	0.1	2.9	0.1
2	83.1	0.1	14.5	0.1	9.0	0.1	2.69	0.01
4	71.7	0.3	24.9	0.9	8.5	0.2	2.31	0.08
24	16	2	79.7	0.5	7.5	0.1	1.23	0.01
47	1.6	0.4	91.6	0.5	7.2	0.1	0.72	0.01
**0.0045 mol% Pt**								
0	100	NA	0	NA	NA	NA	NA	NA
1	93	2	6	2	8.4	0.1	22	7
2	91	3	7	3	8.1	0.6	12	6
4	90.	3	10	5	7.1	0.8	9	5
24	60	13	38	13	5.7	0.1	6	2
48	27	13	73	14	5.5	0.1	5	1
72	9	7	85	7	5.4	0.1	3.9	0.3

a. All reactions performed in duplicate and the numbers provided are averages with the error provided in the column immediately to the right for each value. Values determined by GC-FID. See *General hydrosilylation reaction setup* section for details. b. Values determined by 1H NMR spectroscopy. c. The regioselectivity could not be accurately determined because the α-isomer product signal was too weak to obtain accurate integrals. d. Previous entry analyzed by GC-FID. This shows that the methods are consistent within error of each other.

Hot filtration studies

0.05 mol% Pt: In an argon or nitrogen filled glovebox, precatalyst (5.0 mg) was weighed out in a 1-dram glass vial. Then a solution (0.8-1.6 mL, 0.05 mol% Pt) containing 1-octyne (0.5 M), $PhMe_2SiH$ (0.5 M), and internal standard of cyclooctane (0.15 M) in toluene was added to the vial via syringe. The vial was sealed and placed in a heating block preheated to 80 °C. The reactions involving **2a** and **2c** were heated for 10 m and the reactions involving **2b**, **2d** and **2e** were heated for 3 min. After the allotted time, the reactions were immediately filtered through 0.45 μm PTFE syringe filters into fresh vials while still hot. An aliquot (<0.05 mL) of each filtrate was separated and cooled to -20 °C. The filtered solutions were then returned to the heating block for the same amount of time. The aliquots and final solutions were analyzed by GC-FID after dilution with cyclohexane (see **General considerations** for further details). Unfiltered reactions were performed analogously but the filtration step was skipped. The reaction data is provided in **Figure S3** and **Table S6**.

ICP-OES of the filtered **2d** reaction was performed to determine if P, Zr, and Pt leach into solution. The solution was prepared by removing the toluene from 0.5 mL reaction solution on a rotovaporator which yielded a colorless oil. The majority of the organics were further removed by heating the oil under vacuum (10^{-4} torr) for 2 hours at 150 °C. The residue was soaked in aqua regia (0.25 mL HNO_3/0.75 mL HCl) overnight then diluted to 10 mL and analyzed via ICP-OES. We detected ~6 and ~0.1 mol% of the total initial Pt and Zr added to the reaction, respectively, but did not detect P. The Pt and Zr signals were barely detectable, so it is not surprising we did not observe P due to it being lighter. It is plausible that some of the leached material was lost via aerosolization or sublimation when the organic material was removed. The ^{31}P SSNMR (**Figure S24**) of **2d** (40 mg precatalyst, 10.4 mL stock solution) after hot filtration, washing with toluene (2 X 1 mL), and drying *en vacuo* contains a major signal at 286 ppm, indicating that the $[NHP\text{-}Pt]^+$ species persist on the surface. Growth of a signal at 8 ppm also reveals partial decomposition of the NHP^+ ligand, likely caused by further oxidation from surface pyrosulfates or by an unidentified reaction with the silane.

0.005 mol% Pt: In a nitrogen filled glovebox, **2d** (5.0 mg) was weighed out in a 20 mL glass vial. Then a solution (12.6 mL, 0.005 mol% Pt) containing 1-octyne (0.5 M), $PhMe_2SiH$ (0.5 M), and internal standard of cyclooctane (0.15 M) in toluene was added to the vial via syringe. The vial was sealed and placed in a heating block preheated to 80 °C. After 15 min, the reactions were immediately filtered through a 0.45 μm PTFE syringe filter, 0.45 μm PTFE syringe filter packed with 2 mL of celite, or vacuum filtered with a fine frit with celite (0.75”) into fresh vials while still hot. An aliquot (<0.05 mL) of each filtrate was separated and cooled to -20 °C. The filtered solutions were then returned to the heating block and monitored over time by taking aliquots. The aliquots and final solutions were analyzed by GC-FID after dilution with cyclohexane (see **General considerations** for further details). To determine the effect of each substrate, reactions were conducted in a similar manner in the absence of silane or 1-octyne, which was then added after filtration. The reaction data is provided in **Figure S4** and **Figure S5**, and **Table S7**.

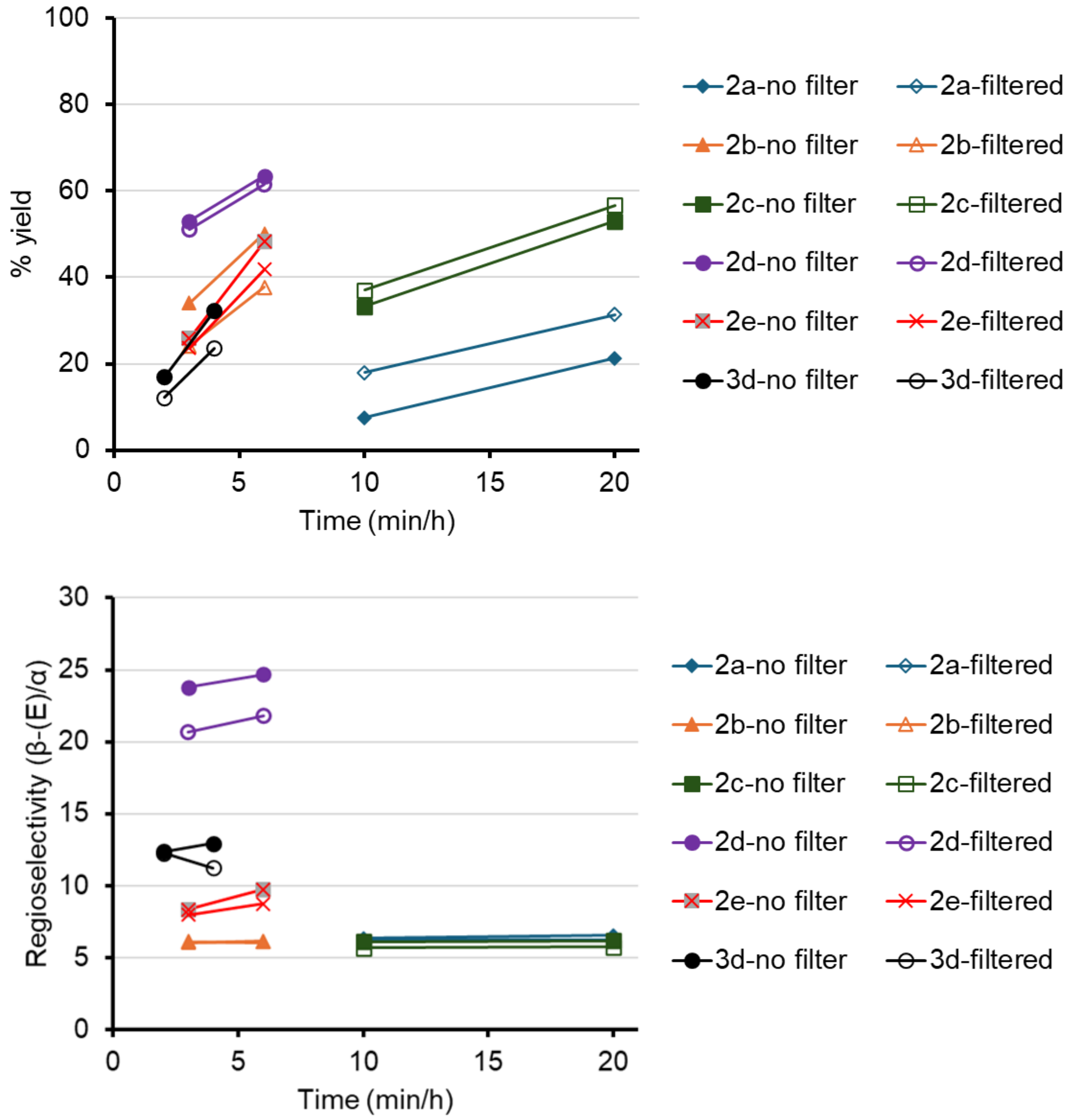

Figure S3. Summary of the hot filtration data for **2a-e** and **3d** at 0.05 mol% Pt compared to unfiltered samples. Filtered samples were filtered at the 1st time point. All times are in min except for the data for **3d** which is h. Top: % yield over time. Bottom: Regioselectivity over time. Values are provided in **Table S6**.

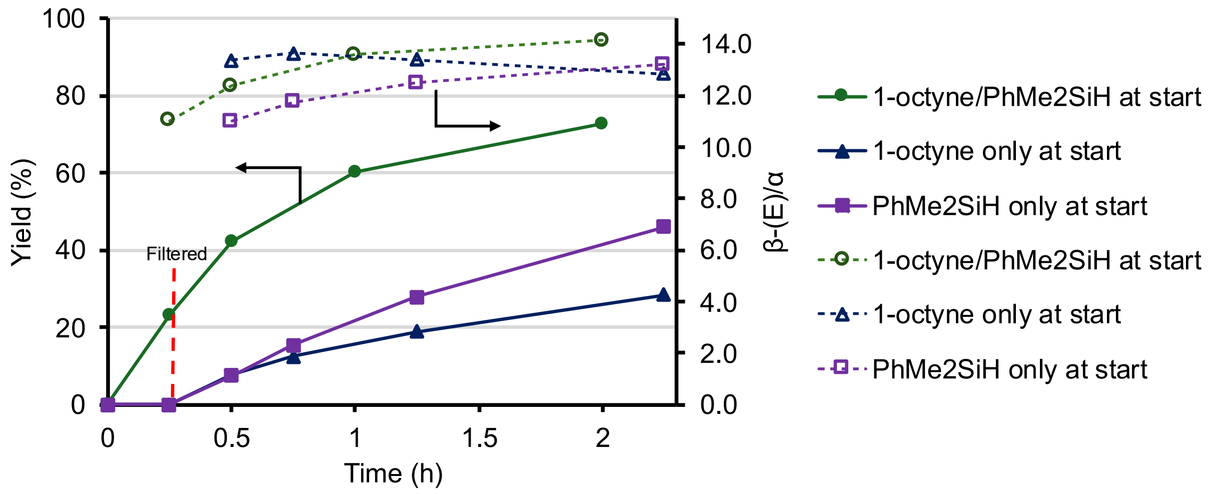

Figure S4. Reaction progression curves for **2d** (0.005 mol% Pt) catalyzed hydrosilylation of 1-octyne with $PhMe_2SiH$. Curves compare hot filtration after 15 minutes under three conditions: (green, circles) both substrates present, (blue, triangles) only 1-octyne before filtration with $PhMe_2SiH$ added after, and (purple, squares) only Me_2PhSiH before filtration with 1-octyne added after. Solid markers and lines represent product yield and open markers and dashed lines represent the regioselectivities. Values provided in **Table S7**.

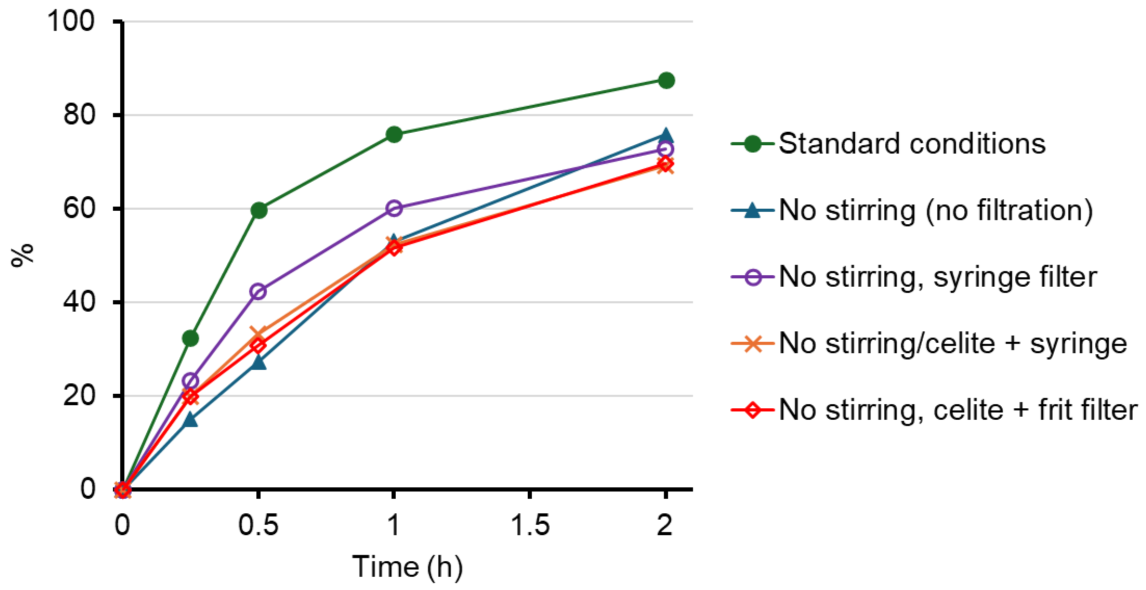

Figure S5. Reaction time plot for **2d** (0.005 mol% Pt) catalyzed hydrosilylation over time comparing different filtration attempts. The filtrations were performed at 15 minutes but no measurable difference in activity over time was observed. Values are provided in **Table S7**. Note that the regioselectivity does not significantly change over time either, suggesting the leached catalyst is a P containing species, not from simple dissociation of Pt from P.

Table S6. Summary of data for the different hot filtrations of **2a-e** and **3d** (0.05 mol% Pt) catalyzed hydrosilylation.[a]

Precatalyst	Time (min)	Filtered?	Silane consumed (%)	Total yield (%)	Yield increase (%)[b]	Regio-selectivity (β-(E)/α)
**2a**	10	No	9	8	182	6.3
	20		23	21		6.5
	10	Yes	19	18	75	6.3
	20		32	31		6.2
**2b**	3	No	42	34	47	6.0
	6		57	50		6.1
	3	Yes	30	24	56	6.1
	6		43	38		6.1
**2c**	10	No	37	33	59	6.1
	20		58	53		6.1
	10	Yes	39	37	53	5.7
	20		59	57		5.7
**2d**	3	No	57	53	20	24
	6		68	64		25
	3	Yes	54	51	20	21
	6		65	62		22
**2e**	3	No	29	26	86	8.3
	6		53	48		9.7
	3	Yes	27	24	78	8.0
	6		46	42		8.7
**3d**	120	No	18	17	90	12
	240		34	32		13
	120	Yes	13	12	93	12
	240		25	24		11

a. Reactions that were filtered, were done so after 3 or 10 min, or 2 h through 0.45 μm syringe filters into a clean vial then heated for another 3- or 10-min, or 2 h. Reactions were monitored by removing aliquots at the given times which were analyzed by GC-FID. Full details are provided above at the beginning of the *Hot filtration studies* section. b. Yield increase (%) was calculated by the following equation: ((% total yield @ end time- % total yield @ 1st time point)/(% total yield @ 1st time point))*100.

Table S7. Summary of data for the different hot filtrations for **2d** (0.005 mol% Pt) catalyzed hydrosilylation.[a]

**Time (h)**	**Silane consumed (%)**	**Total yield (%)**	**Regio-selectivity (β-(E)/α)**
No filter			
0.25 (filtered)	21	15	12
0.5	31	27	12
1	58	53	15
2	81	76	16
0.45 μm PTFE syringe filter			
0.25 (filtered)	25	23	11
0.5	44	42	12
1	61	60	14
2	75	73	14
0.45 μm PTFE syringe filter + celite (2 mL)			
0.25 (filtered)	22	20	11
0.5	35	33	13
1	55	52	15
2	73	69	16
Fine glass frit + celite (3/4')			
0.25 (filtered)	22	20	11
0.5	34	31	12
1	56	52	13
2	73	70	15
1-octyne only initially, silane added after filtration, 0.45 μm PTFE syringe filter			
0.25 (filtered)	N.A.	N.A.	N.A.
0.5	12	8	13
0.75	15	12	14
1.25	21	19	13
2.25	30	28	13
Silane only initially, 1-octyne added after filtration, 0.45 μm PTFE syringe filter			
0.25 (filtered)	3	N.A.	N.A.
0.5	12	8	11
0.75	20	15	12
1.25	33	28	13
2.25	52	46	13

a. Reactions were filtered after 15 min and monitored by removing aliquots at the given times which were analyzed by GC-FID. The differences in conditions are provided in the merged cell above each set of data. Full details are provided above at the beginning of the *Hot filtration studies* section. N.A. = not applicable.

Catalyst reuse study

In a glovebox, **2d** (5.0 mg, 0.32 μmol) was weighed into an NMR tube. Then a solution (0.8 mL) containing 1-octyne (0.5 M), $PhMe_2SiH$ (0.5 M), and internal standard of cyclooctane (0.15 M) in toluene was added to the tube via syringe. The NMR tube was sealed and placed in a heating block on a hot plate (preheated to 80 °C). The reaction was heated for 30 min then the solution was decanted from the NMR tube while hot, filtered (45 μm), and analyzed by GC-FID. The remaining solid catalyst was washed with toluene (1 mL) which was removed by decantation. The reaction was repeated with the residual catalyst 4 times. A summary of the results is provided in **Figure S6** and **Table S8**.

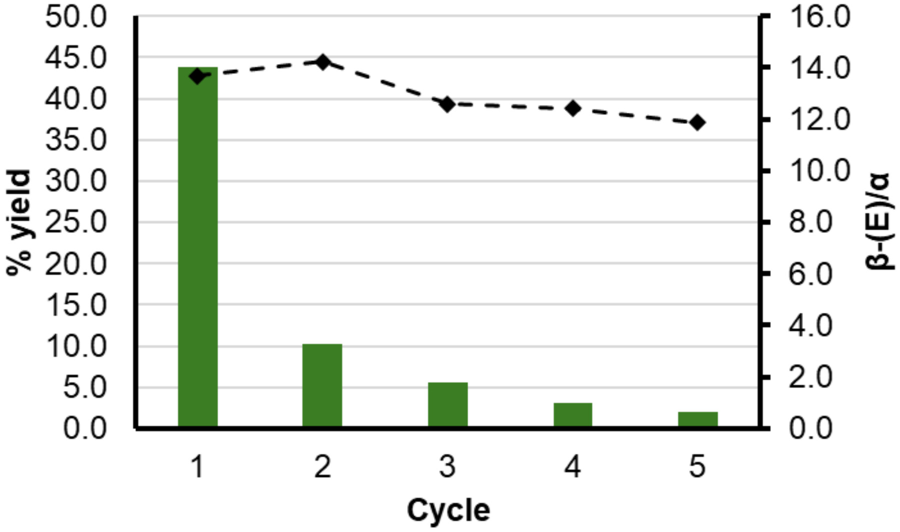

Figure S6. Plot of **2d** (0.08 mol% Pt) catalyzing 1-octyne hydrosilylation followed by filtration and reuse of the solid catalyst 4 times. Green bars represent the total product yield (%) and the black diamonds represent the regioselectivity for each cycle.

Table S8. Summary of the reuse data for **2d**.[a]

Cycle	Total yield (%)	Regioselectivity (β-(E)/α)
1	43.9	13.7
2	10.3	14.2
3	5.6	12.6
4	3.1	12.4
5	2.0	11.9

a. See above section for details.

Homogeneous catalysis

In a nitrogen filled glovebox, the catalysts were generated *in situ* by combining 18 μL of 2.8 mM Karstedt's catalyst diluted in toluene (0.05 μmol, 0.01 mol% Pt) with 18 μL of 4.2 mM of ligand in fluorobenzene for MesNHPOTf and [MesNHP][B(C_6F_5)$_4$] (not soluble in toluene) or toluene for PhNHPOTf and MeNHPOTf (0.076 μmol P) in a 1 dram vial then gently shaken together. Then a solution (1.0 mL) containing 1-octyne (0.5 M, 0.5 mmol), $PhMe_2SiH$ (0.5 M, 0.5 mmol), and internal standard of cyclooctane in toluene was added to the vial via syringe. The vial was sealed and placed in a heating block preheated to 80 °C for 1 h then cooled to room temperature and analyzed by GC-FID. The results are summarized in **Table 2** of the main text.

FTIR and solid-state NMR spectra

DRIFT spectra

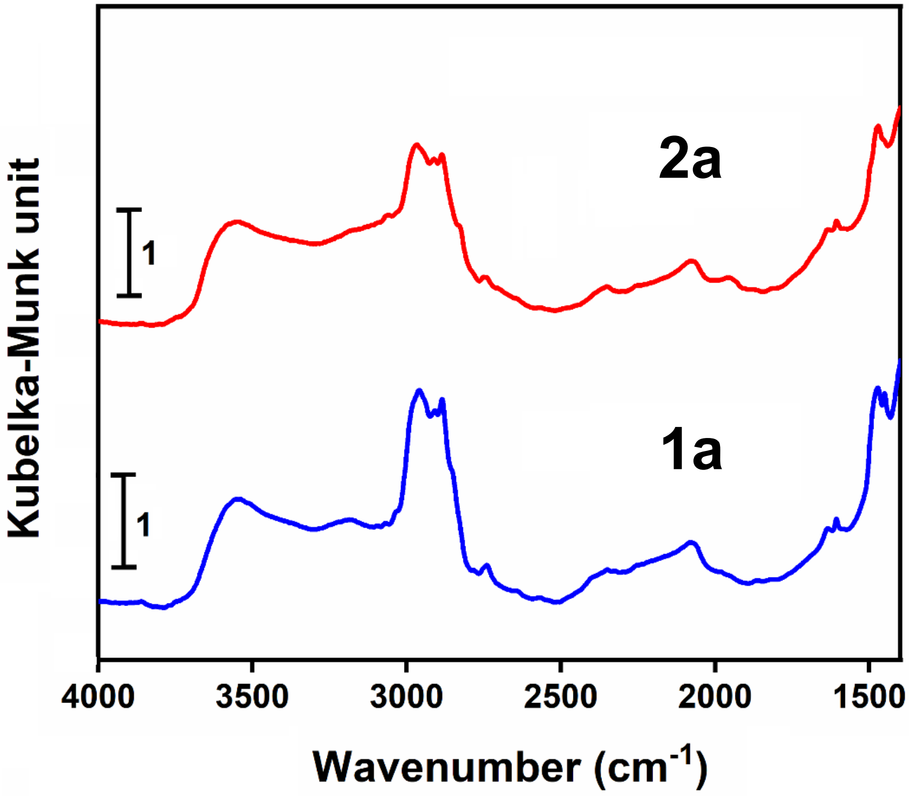

Figure S7. DRIFT spectrum of **1a** and **2a**. The bar denotes the relative unit scale.

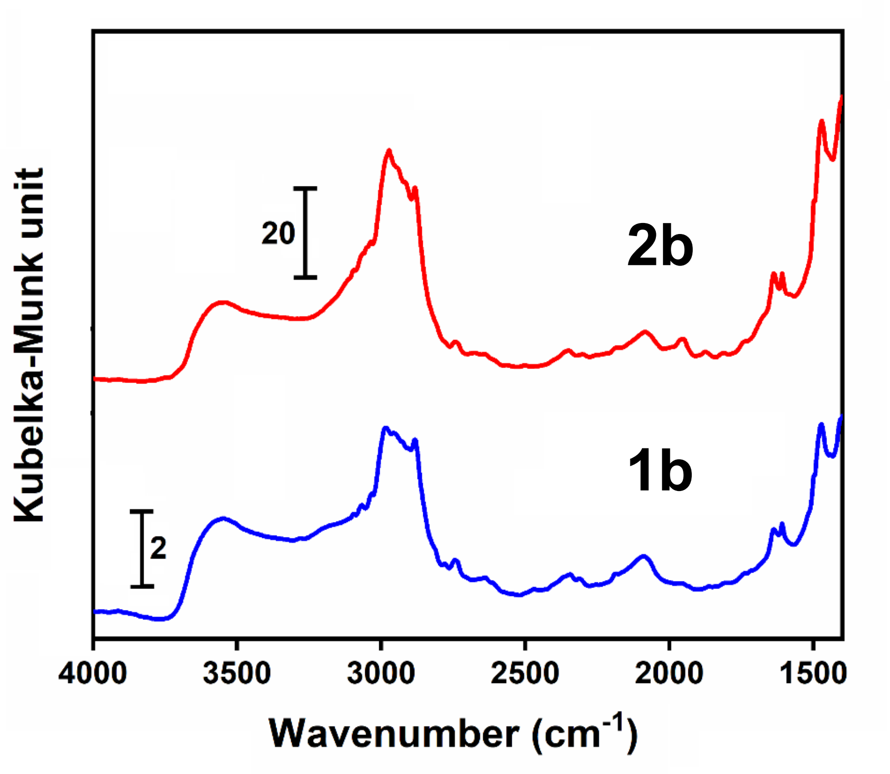

Figure S8. DRIFT spectrum of **1b** and **2b**. The bar denotes the relative unit scale.

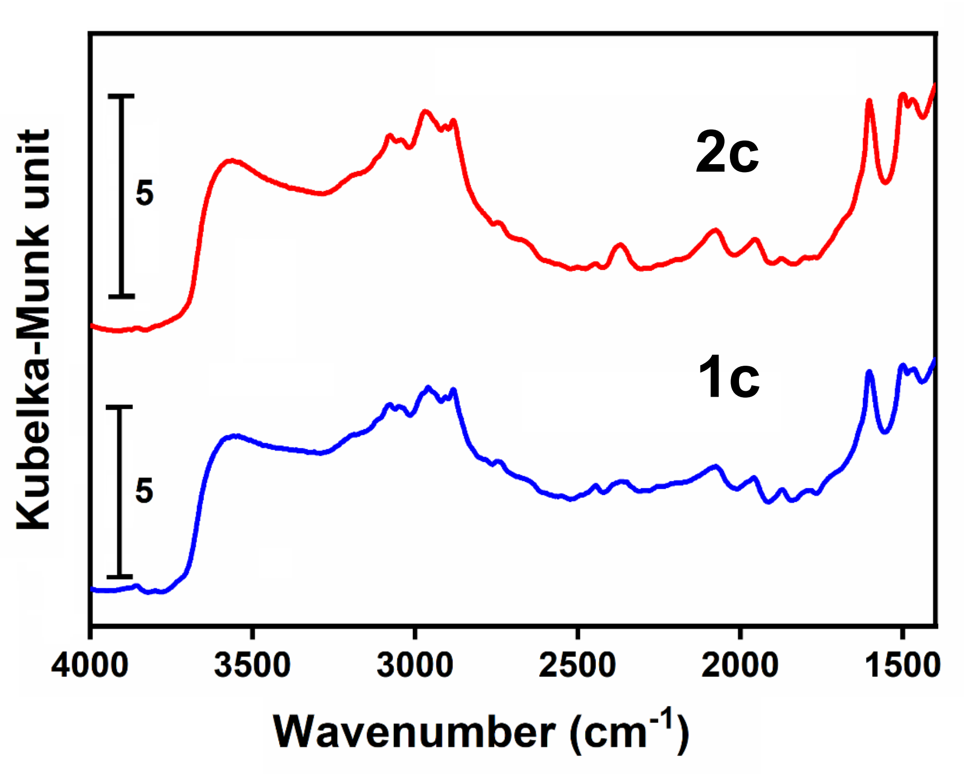

Figure S9. DRIFT spectrum of **1c** and **2c.** The bar denotes the relative unit scale.

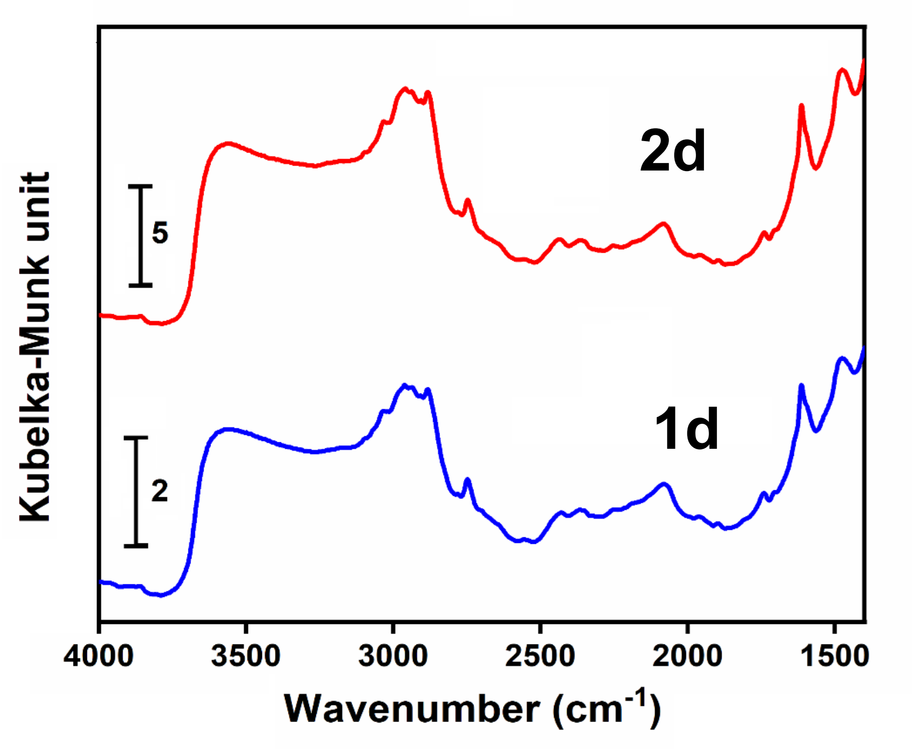

Figure S10. DRIFT spectrum of **1d** and **2d.** The bar denotes the relative unit scale.

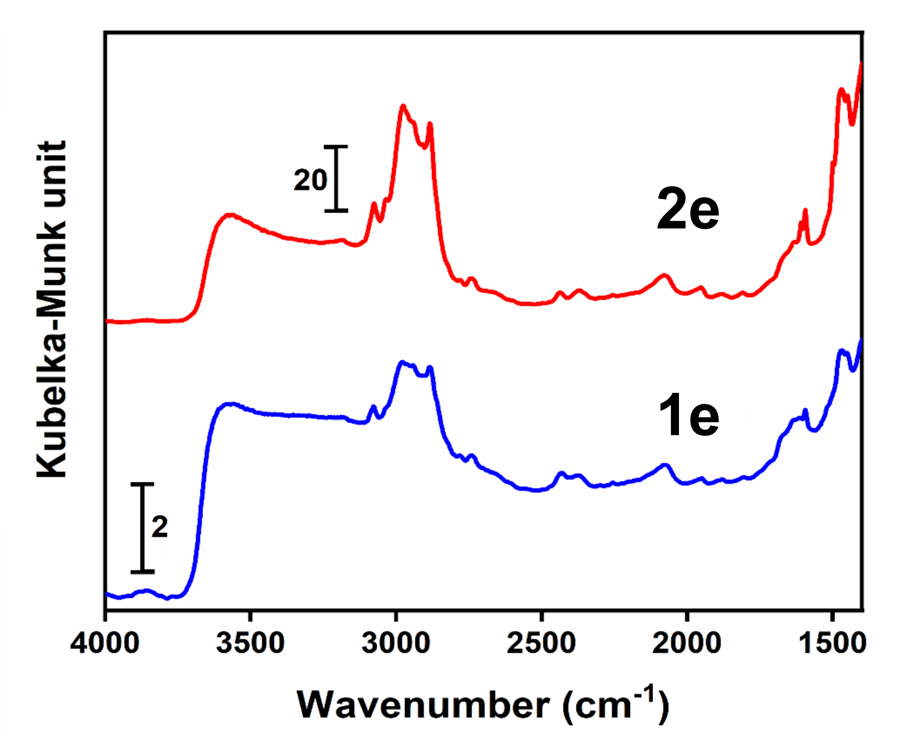

Figure S11. DRIFT spectrum of **1e** and **2e.** The bar denotes the relative unit scale.

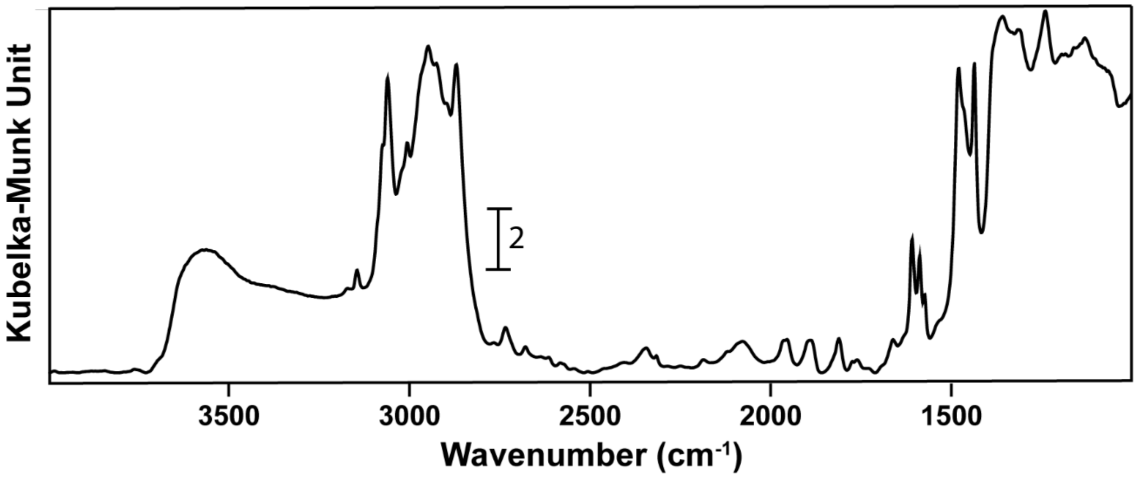

Figure S12. DRIFT spectrum of **3d**. The bar denotes the relative unit scale.

*SSNMR spectra of **1a-e***

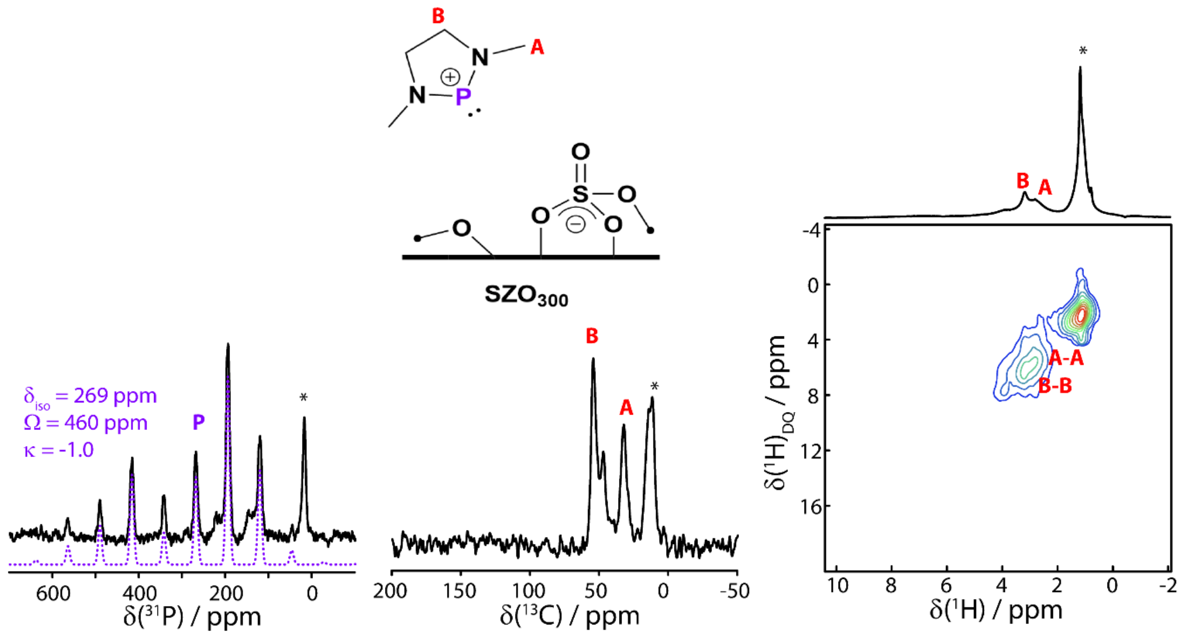

Figure S13. SSNMR spectra of **1a** with assignments. Left: ^{31}P CPMAS NMR spinning at 12 kHz at 100 K. Isotropic shift is identified with the **P**. The top black spectrum is the experimental spectrum and the bottom purple dashed spectrum is the best fit simulation used to determine the CSA. *Unknown impurity likely due to a small amount of oxidation from surface pyrosulfates during the ligand grafting process. Middle: ^{13}C CPMAS NMR spinning at 12 kHz at 100 K. * indicates residual $^{i}Pr_3Si$- signals. Right: ^{1}H DQ/SQ correlation NMR spinning at 37.037 kHz at room temperature. * indicates residual $^{i}Pr_3Si$- signals.

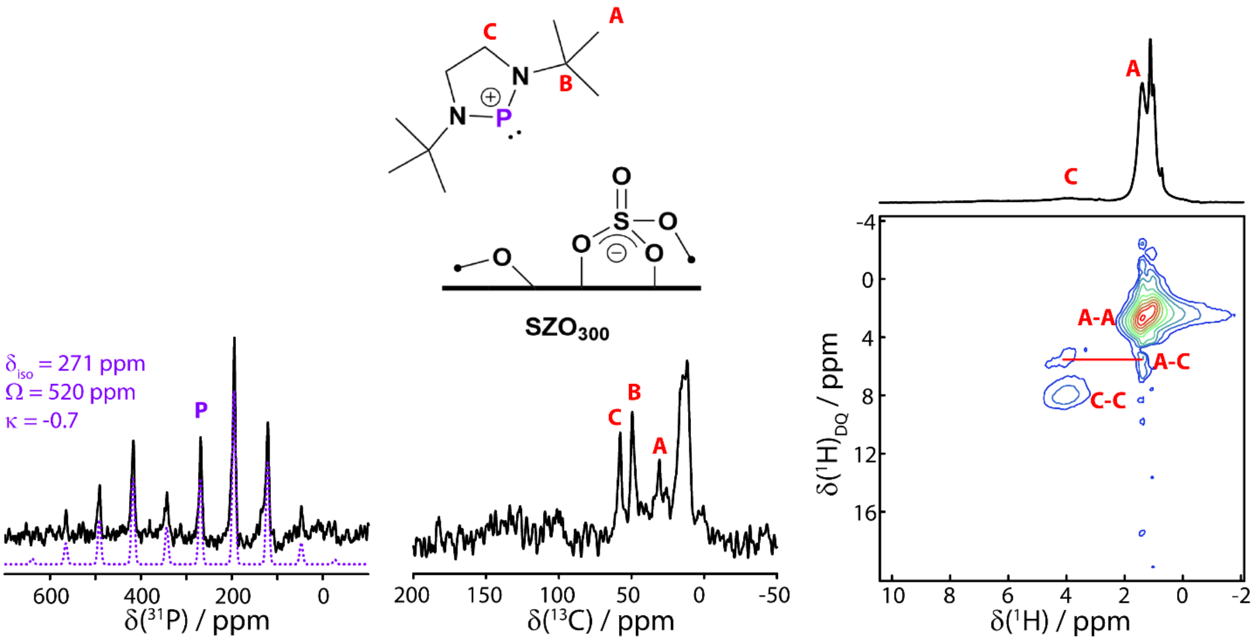

Figure S14. SSNMR spectra of **1b** with assignments. Left: ^{31}P CPMAS NMR spinning at 12 kHz at 100 K. Isotropic shift is identified with the **P**. The top black spectrum is the experimental spectrum and the bottom purple dashed spectrum is the best fit simulation used to determine the CSA. Middle: ^{13}C CPMAS NMR spinning at 12 kHz at 100 K. * indicates residual $^{i}Pr_3Si$- signals. Right: ^{1}H DQ/SQ correlation NMR spinning at 37.037 kHz at room temperature. * indicates residual $^{i}Pr_3Si$- signals.

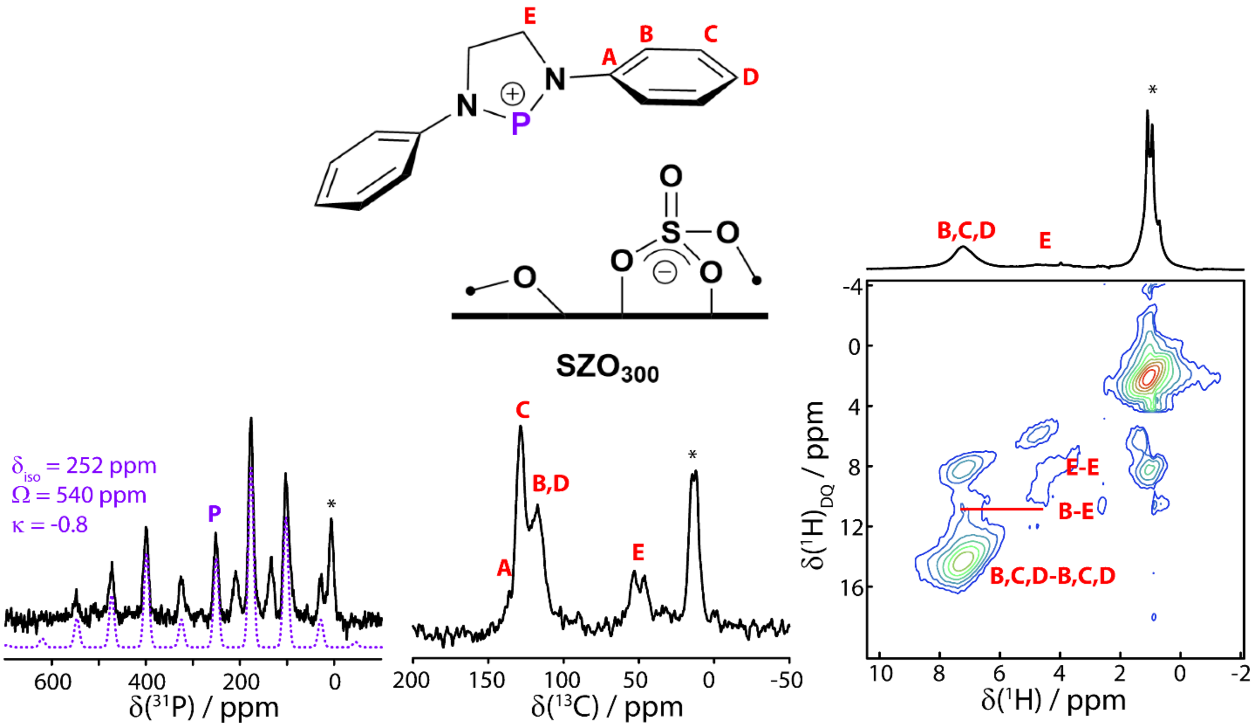

Figure S15. SSNMR spectra of **1c** with assignments. Left: ^{31}P CPMAS NMR spinning at 12 kHz at 100 K. Isotropic shift is identified with the **P**. The top black spectrum is the experimental spectrum, and the bottom purple dashed spectrum is the best fit simulation used to determine the CSA. *Unknown impurity likely due to a small amount of oxidation from surface pyrosulfates during the ligand grafting process. Middle: ^{13}C CPMAS NMR spinning at 12 kHz at 100 K. * indicates residual ^{i}Pr$_3$Si- signals. Right: ^{1}H DQ/SQ correlation NMR spinning at 37.037 kHz at room temperature. * indicates residual ^{i}Pr$_3$Si- signals.

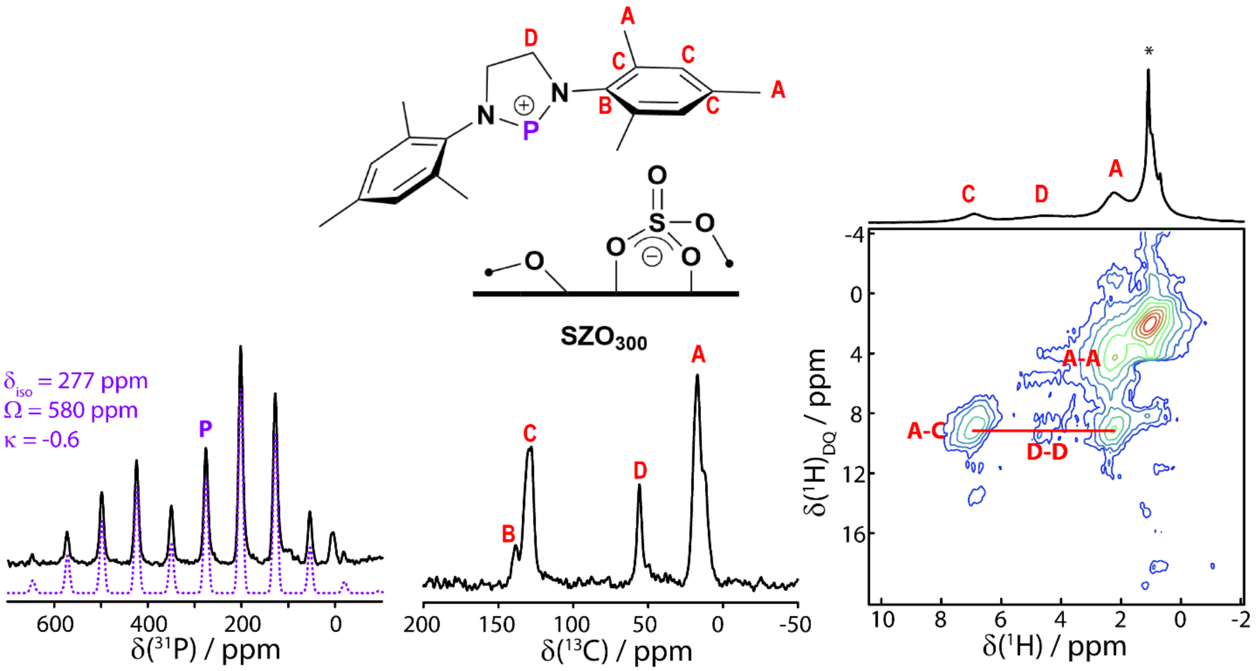

Figure S16. SSNMR spectra of **1d** with assignments. Left: ^{31}P CPMAS NMR spinning at 12 kHz at 100 K. Isotropic shift is identified with the **P**. The top black spectrum is the experimental spectrum and the bottom purple dashed spectrum is the best fit simulation used to determine the CSA. Minor impurity near 0 ppm likely due to a small amount of oxidation from surface pyrosulfates during the ligand grafting process. Middle: ^{13}C CPMAS NMR spinning at 12 kHz at 100 K. * indicates residual $^{i}Pr_3Si$- signals. Right: ^{1}H DQ/SQ correlation NMR spinning at 37.037 kHz at room temperature. * indicates residual $^{i}Pr_3Si$- signals.

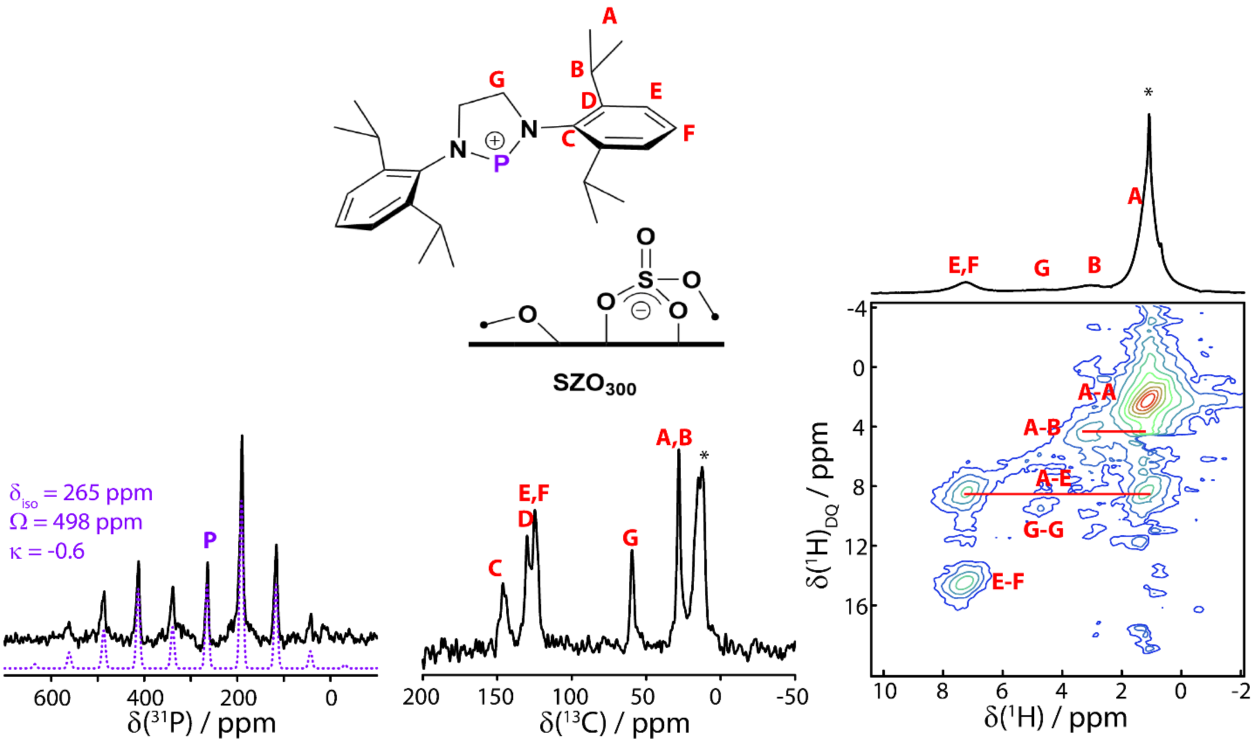

Figure S17. SSNMR spectra of **1e** with assignments. Left: ^{31}P CPMAS NMR spinning at 12 kHz at 100 K. Isotropic shift is identified with the **P**. The top black spectrum is the experimental spectrum and the bottom purple dashed spectrum is the best fit simulation used to determine the CSA. Middle: ^{13}C CPMAS NMR spinning at 12 kHz at 100 K. * indicates residual ^{i}Pr$_3$Si- signals. Right: ^{1}H DQ/SQ correlation NMR spinning at 37.037 kHz at room temperature. * indicates residual ^{i}Pr$_3$Si- signals.

*SSNMR spectra of **2a-e***

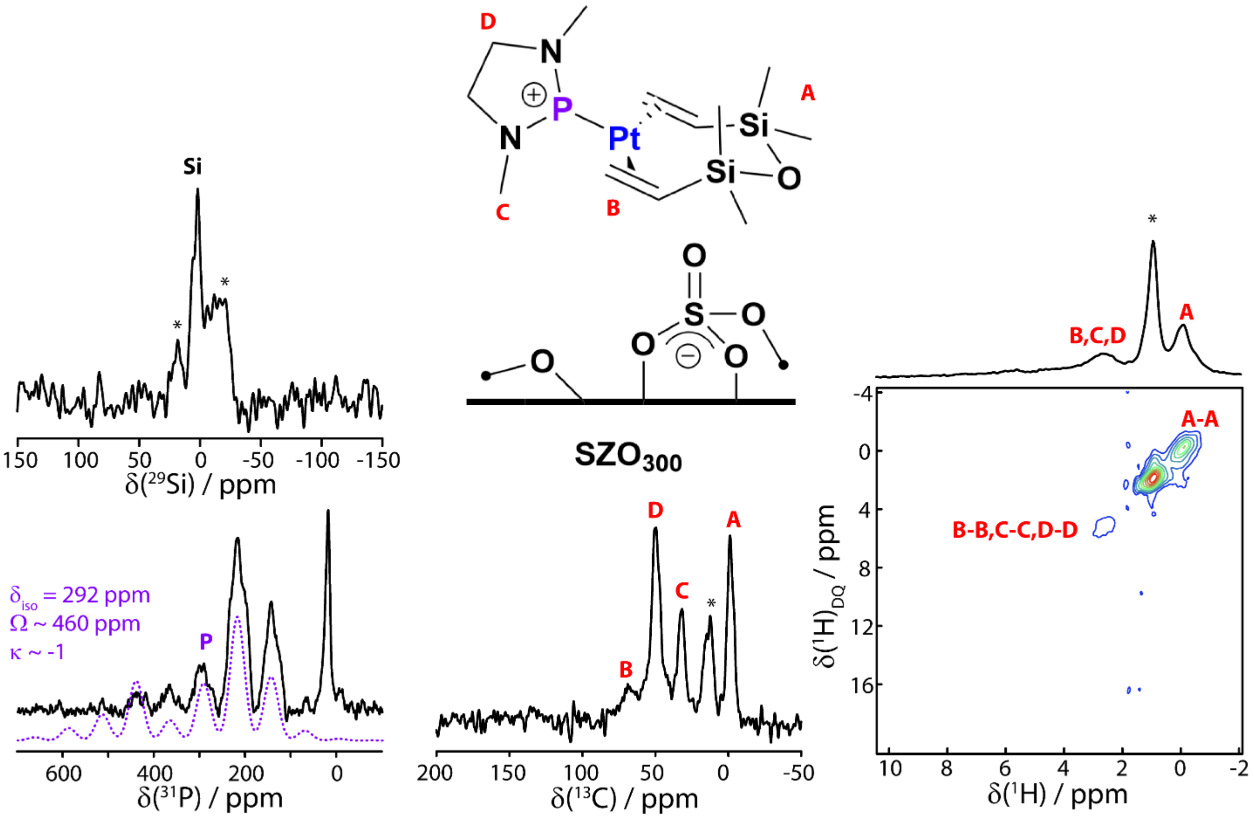

Figure S18. SSNMR spectra of **2a** with assignments. Top left: ^{29}Si CPMAS NMR spinning at 12 kHz at 100 K. * indicates residual ^{i}Pr$_3$Si-O residual signals (~15 ppm) and unknown Si impurities (below 2 ppm). Left: ^{31}P CPMAS NMR spinning at 12 kHz at 100 K. Isotropic shift is identified with the **P**. The top black spectrum is the experimental spectrum and the bottom purple dashed spectrum is the best fit simulation used to determine the CSA. Note the asymmetric broadening of the spinning sidebands from dynamic processes. *Unknown impurity near 0 ppm likely due to a small amount of oxidation from surface pyrosulfates during the ligand grafting process. Middle: ^{13}C CPMAS NMR spinning at 12 kHz at 100 K. * indicates residual ^{i}Pr$_3$Si- signals. Right: ^{1}H DQ/SQ correlation NMR spinning at 37.037 kHz at room temperature. * indicates residual ^{i}Pr$_3$Si- signals.

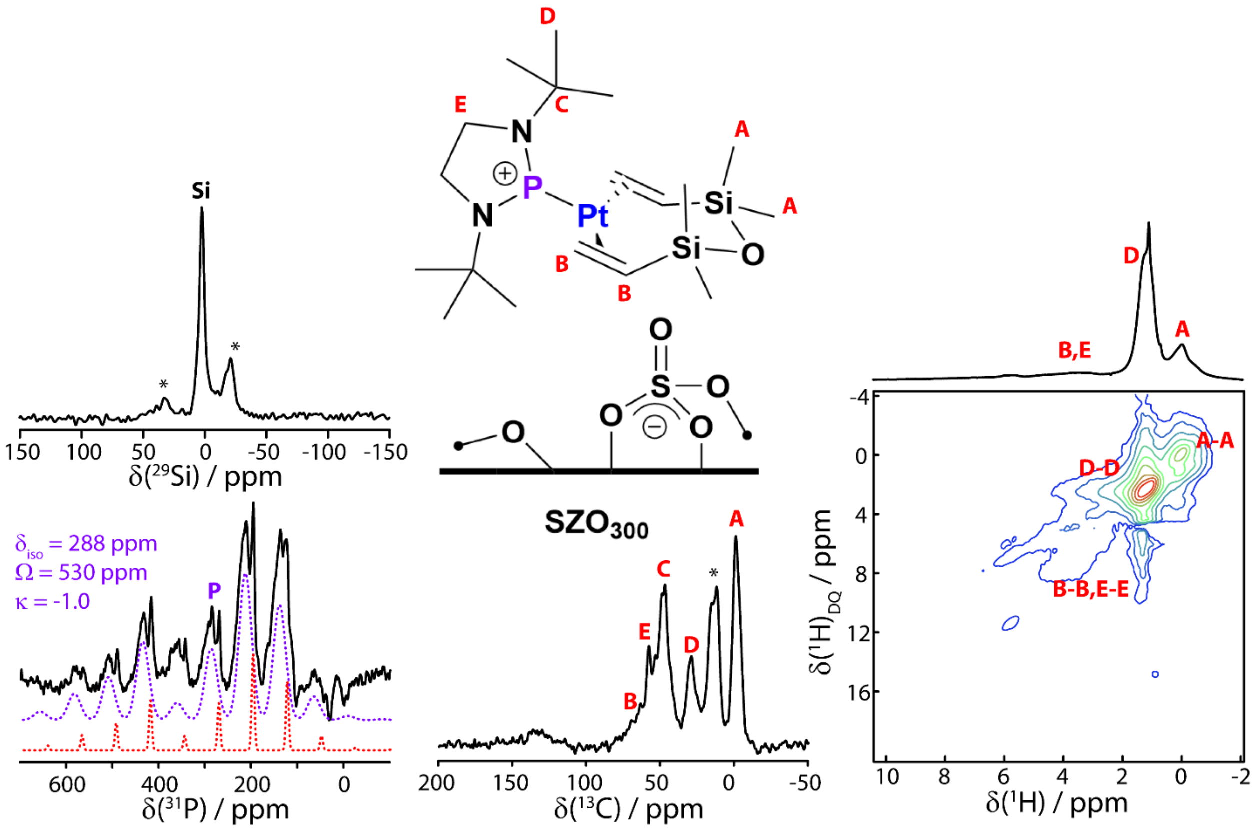

Figure S19. SSNMR spectra of **2b** with assignments. Top left: ^{29}Si CPMAS NMR spinning at 12 kHz at 100 K. * indicates residual ^{i}Pr$_3$Si-O residual signals (~15 ppm) and unknown Si impurities (below 2 ppm). Left: ^{31}P CPMAS NMR spinning at 12 kHz at 100 K. Isotropic shift is identified with the **P**. The top black spectrum is the experimental spectrum, the middle purple dashed spectrum is the best fit simulation of the P-Pt signal used to determine the CSA, and the bottom red dashed simulated spectrum highlights the unreacted [^{t}BuNHP]$^{+}$ sites. Middle: ^{13}C CPMAS NMR spinning at 12 kHz at 100 K. * indicates residual ^{i}Pr$_3$Si- signals. Right: ^{1}H DQ/SQ correlation NMR spinning at 37.037 kHz at room temperature. * indicates residual ^{i}Pr$_3$Si- signals.

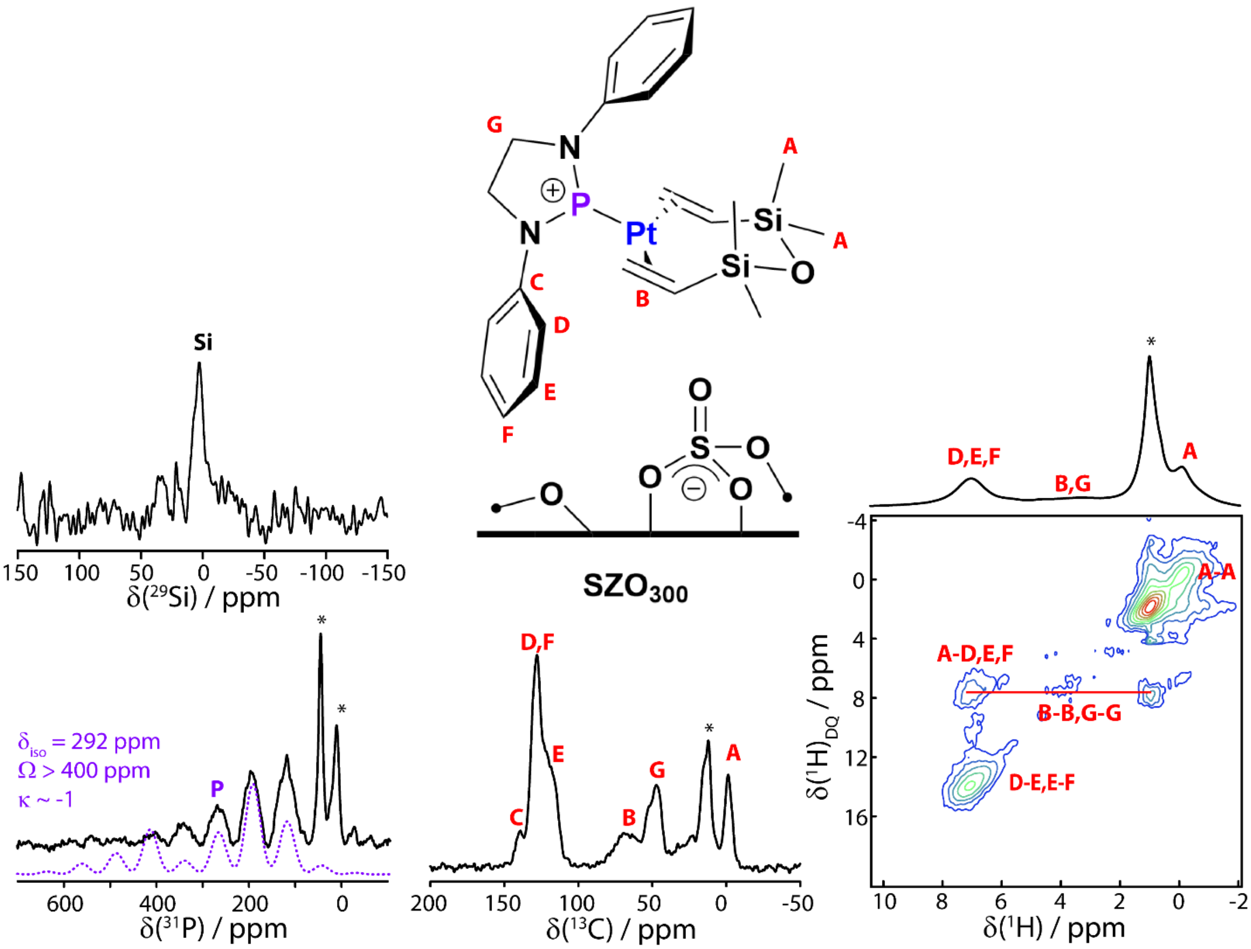

Figure S20. SSNMR spectra of **2c** with assignments. Top left: ^{29}Si CPMAS NMR spinning at 12 kHz at 100 K. Left: ^{31}P CPMAS NMR spinning at 12 kHz at 100 K. Isotropic shift is identified with the **P**. The top black spectrum is the experimental spectrum and the bottom purple dashed spectrum is the best fit simulation used to determine the CSA. Note the asymmetric broadening of the spinning sidebands from dynamic processes. *Unknown impurities likely due to a small amount of oxidation from surface pyrosulfates during the ligand grafting process. Middle: ^{13}C CPMAS NMR spinning at 12 kHz at 100 K. * indicates residual $^{i}Pr_3Si$- signals. Right: ^{1}H DQ/SQ correlation NMR spinning at 37.037 kHz at room temperature. * indicates residual $^{i}Pr_3Si$- signals.

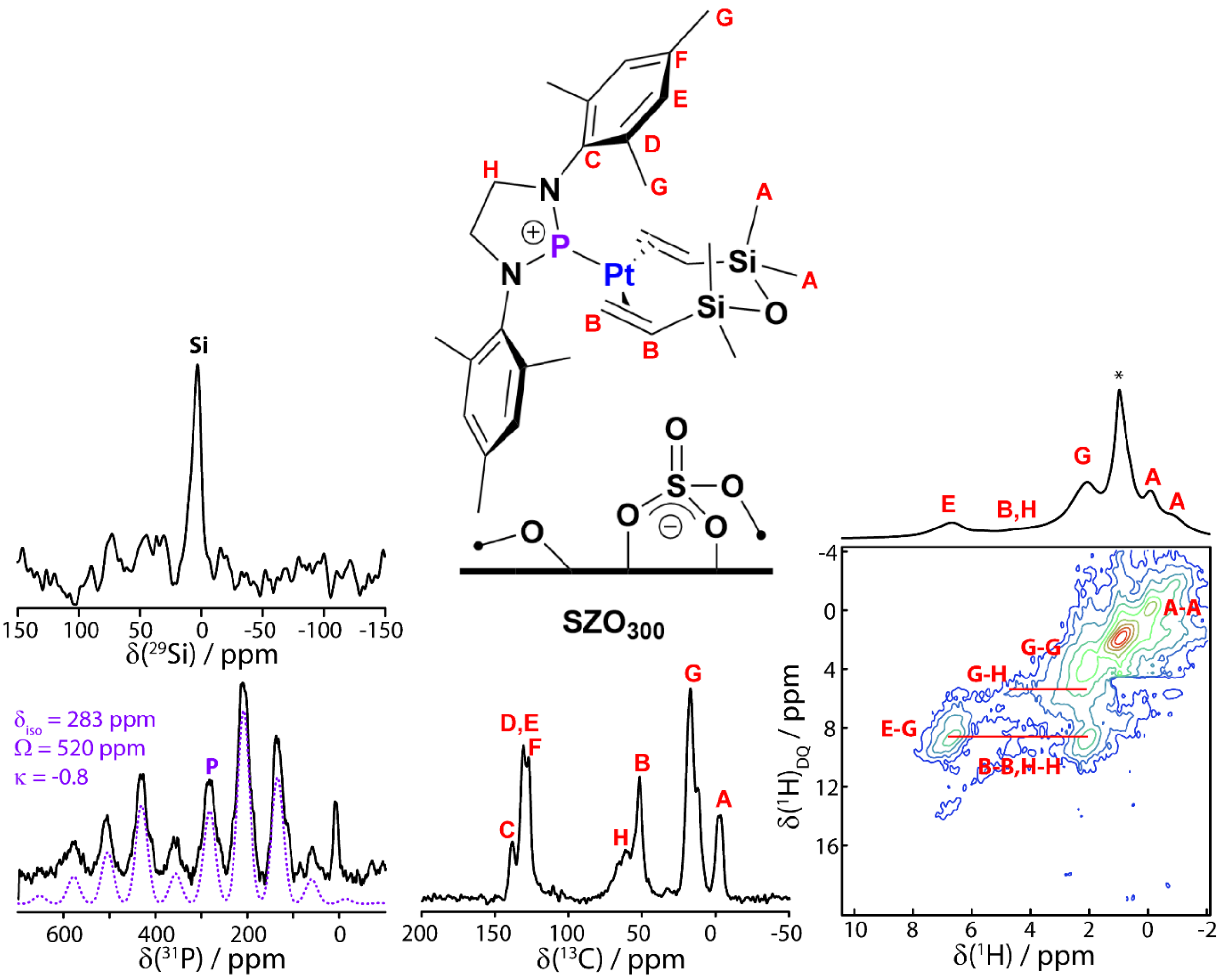

Figure S21. SSNMR spectra of **2d** with assignments. Top left: ^{29}Si CPMAS NMR spinning at 12 kHz at 100 K. Left: ^{31}P CPMAS NMR spinning at 12 kHz at 100 K. Isotropic shift is identified with the **P**. The top black spectrum is the experimental spectrum and the bottom purple dashed spectrum is the best fit simulation used to determine the CSA. *Unknown impurity near 0 ppm likely due to a small amount of oxidation from surface pyrosulfates during the ligand grafting process. Middle: ^{13}C CPMAS NMR spinning at 12 kHz at 100 K. * indicates residual $^{i}Pr_3Si$- residual signals. Right: ^{1}H DQ/SQ correlation NMR spinning at 37.037 kHz at room temperature. * indicates residual $^{i}Pr_3Si$- signals.

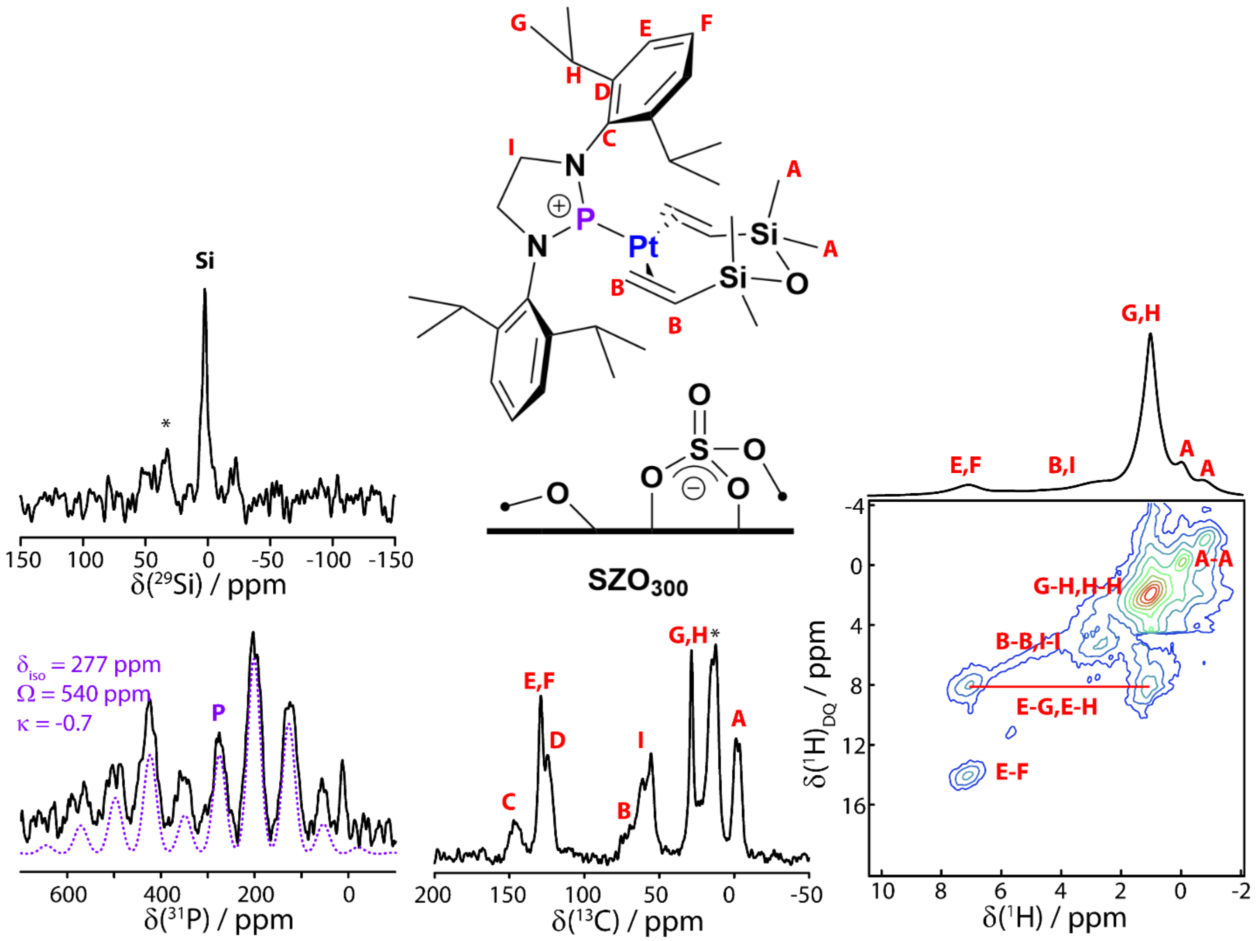

Figure S22. SSNMR spectra of **2e** with assignments. Top left: ^{29}Si CPMAS NMR spinning at 12 kHz at 100 K. * indicates residual $^{i}Pr_3Si$- residual signals. Left: ^{31}P CPMAS NMR spinning at 12 kHz at 100 K. Isotropic shift is identified with the **P**. The top black spectrum is the experimental spectrum and the bottom purple dashed spectrum is the best fit simulation used to determine the CSA. *Unknown impurity near 0 ppm likely due to a small amount of oxidation from surface pyrosulfates during the ligand grafting process. Middle: ^{13}C CPMAS NMR spinning at 12 kHz at 100 K. * indicates residual $^{i}Pr_3Si$- signals. Right: ^{1}H DQ/SQ correlation NMR spinning at 37.037 kHz at room temperature. Residual $^{i}Pr_3Si$- residual signals overlap with **G** and **H**.

*SSNMR spectra of **3d***

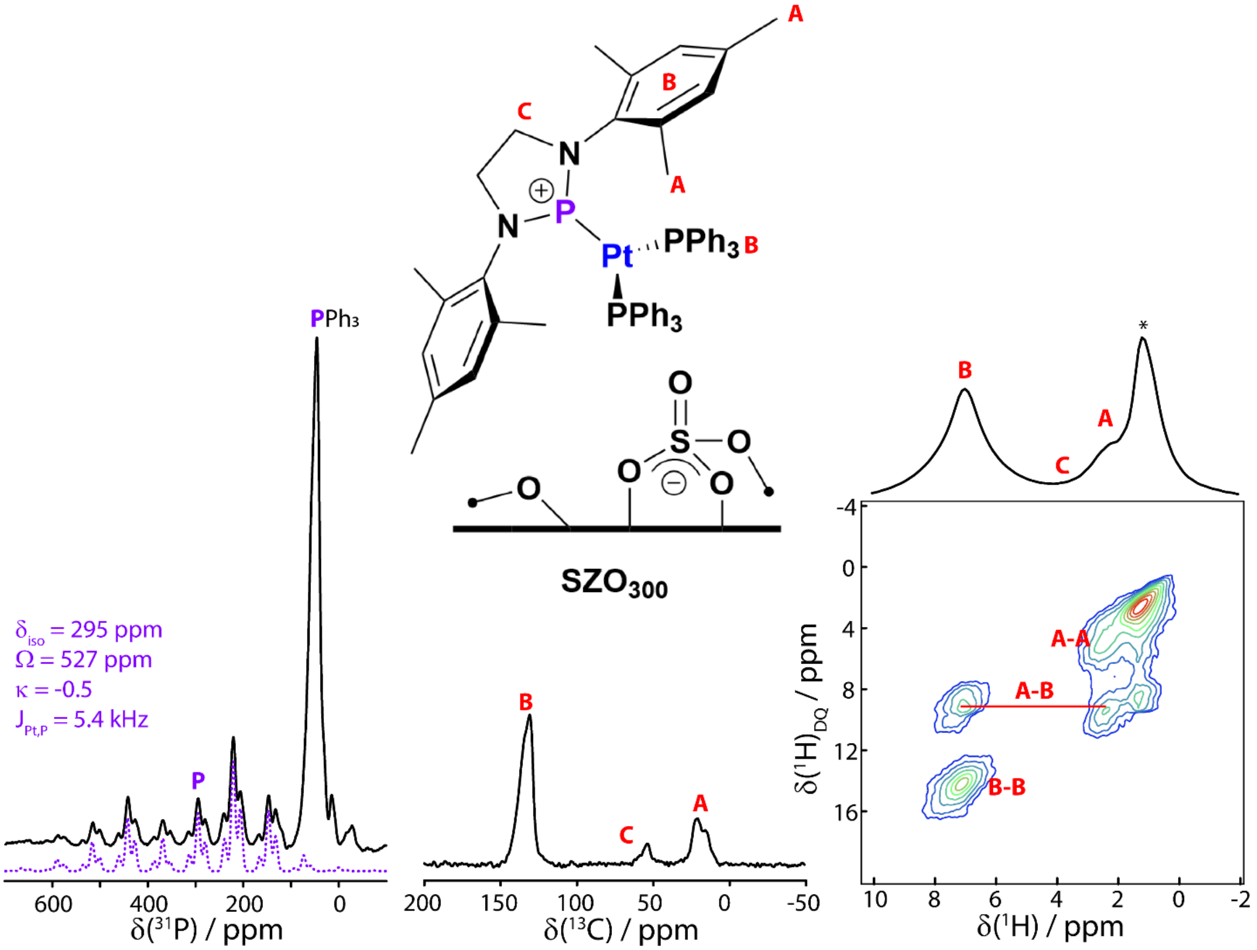

Figure S23. SSNMR spectra of **3d** with assignments. Left: ^{31}P CPMAS NMR spinning at 12 kHz at 100 K. Isotropic shift is identified with the **P**. The top black spectrum is the experimental spectrum and the bottom purple dashed spectrum is the best fit simulation used to determine the CSA. Middle: ^{13}C CPMAS NMR spinning at 12 kHz at 100 K. Residual $^{i}Pr_3Si$- signals overlap with **A**. Right: ^{1}H DQ/SQ correlation NMR spinning at 37.037 kHz at room temperature. * indicates residual $^{i}Pr_3Si$- residual signals.

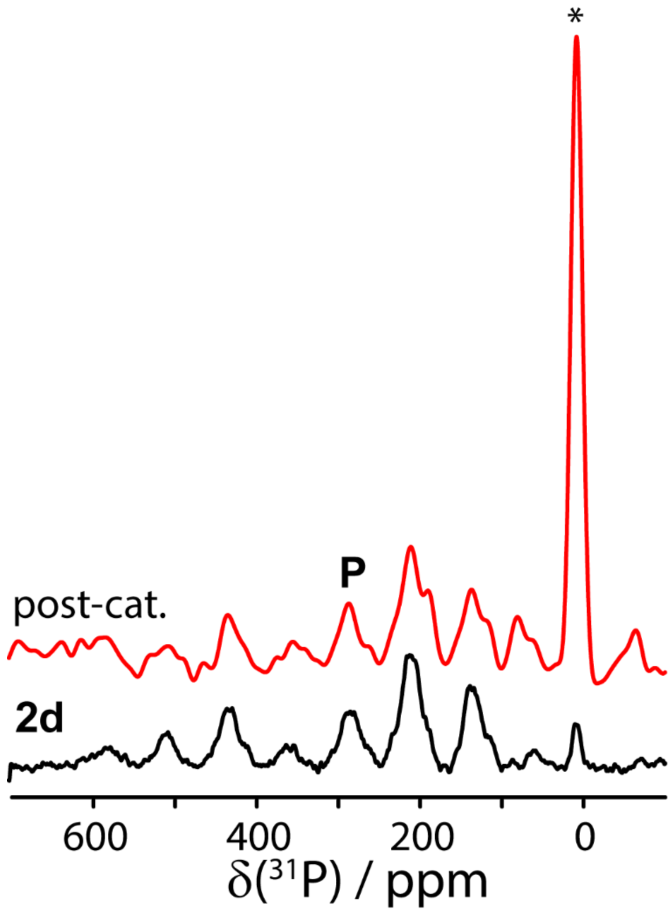

Figure S24. ^{31}P CPMAS NMR spectra of **2d** (bottom) and **2d** (top) after catalysis and hot-filtration spinning at 12 kHz at 100 K. Isotropic shift is identified with the **P**. *Unknown impurity at 8 ppm likely due to oxidation by pyrosulfates or unknown reaction with Me_2PhSiH.

Solution NMR spectra and gas chromatograph

Mass balance

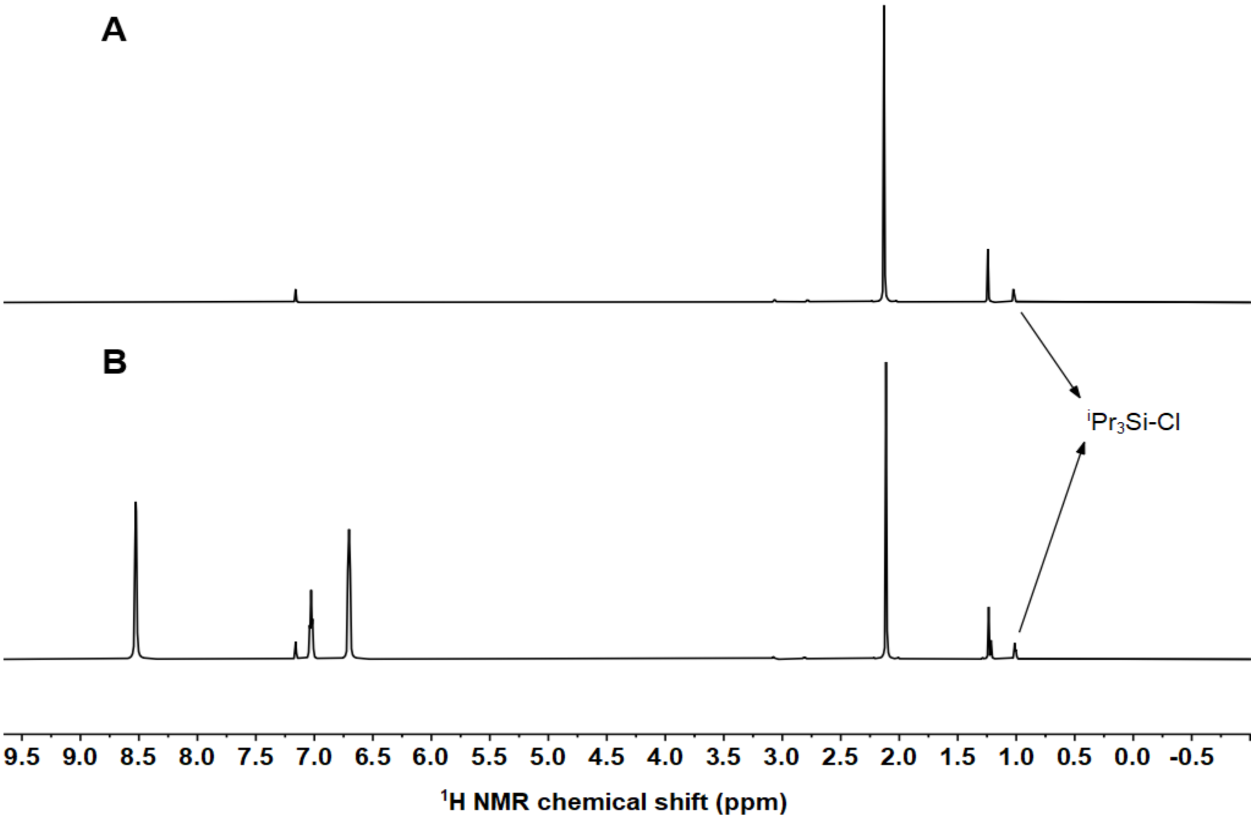

Figure S25. Representative ^{1}H NMR spectra of the reaction of [$^{i}Pr_3Si$][SZO] and ^{t}BuNHPCl before (A) and after adding pyridine (B) in benzene-d_6.

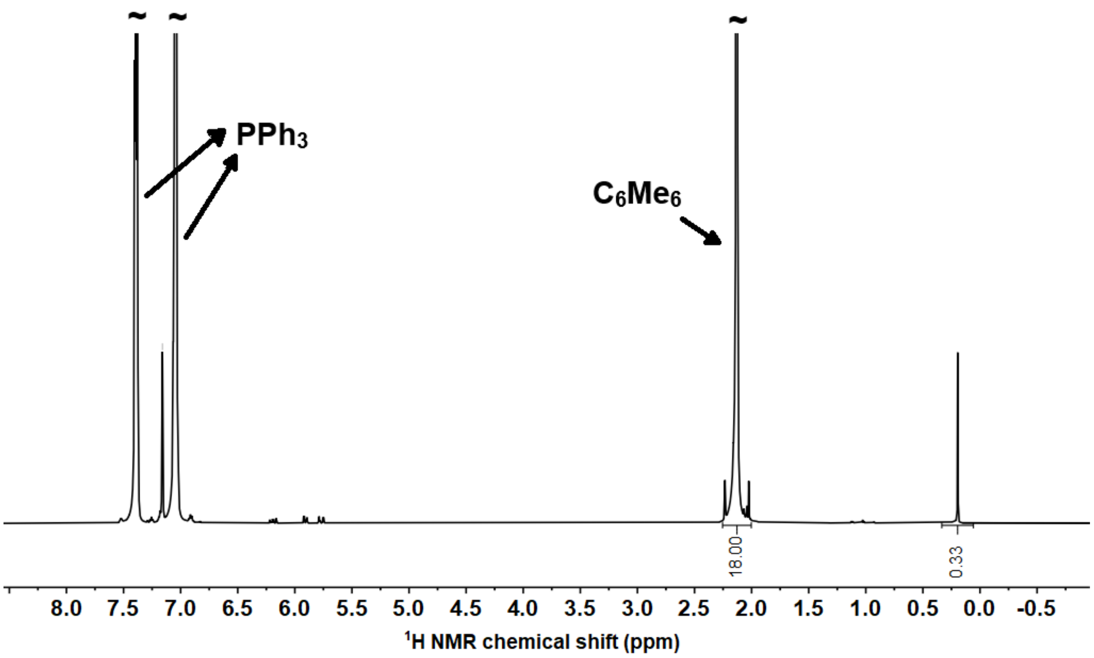

Figure S26. Representative ^{1}H NMR spectrum of the reaction of **2d** with excess PPh_3 in benzene-d_6 with hexamethylbenzene as an internal standard. ~ denote PPh_3 (7.39 and 7.04 ppm), hexamethylbenzene (~2.13 ppm) signal cutoffs.

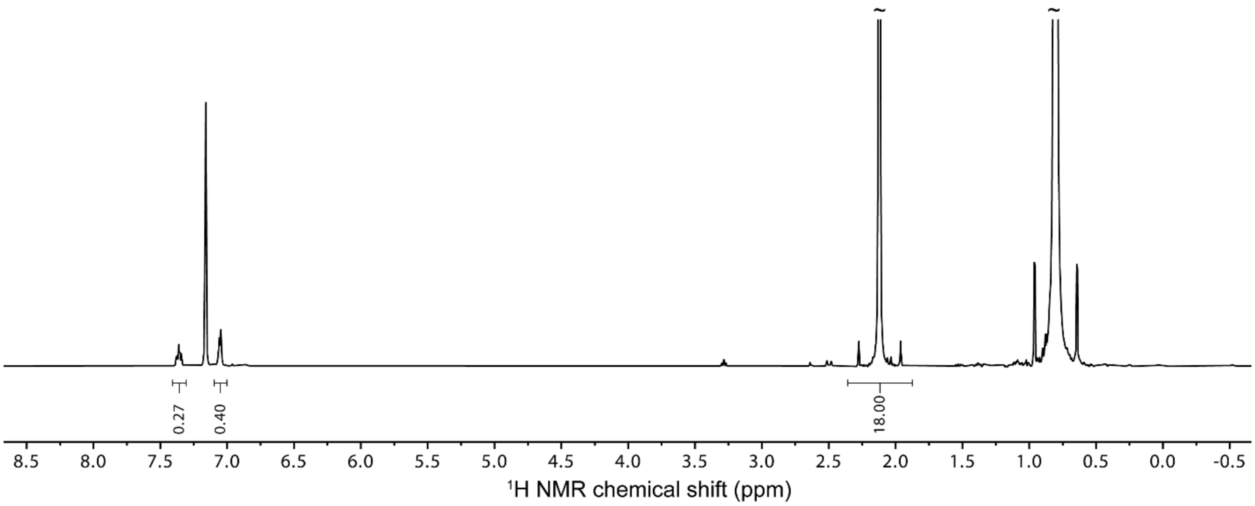

Figure S27. Representative ^{1}H NMR spectrum of the reaction of **3d** with PMe_3 in benzene-d_6. ~ denote PMe_3 and hexamethylbenzene (~2.13 ppm) signal cutoffs.

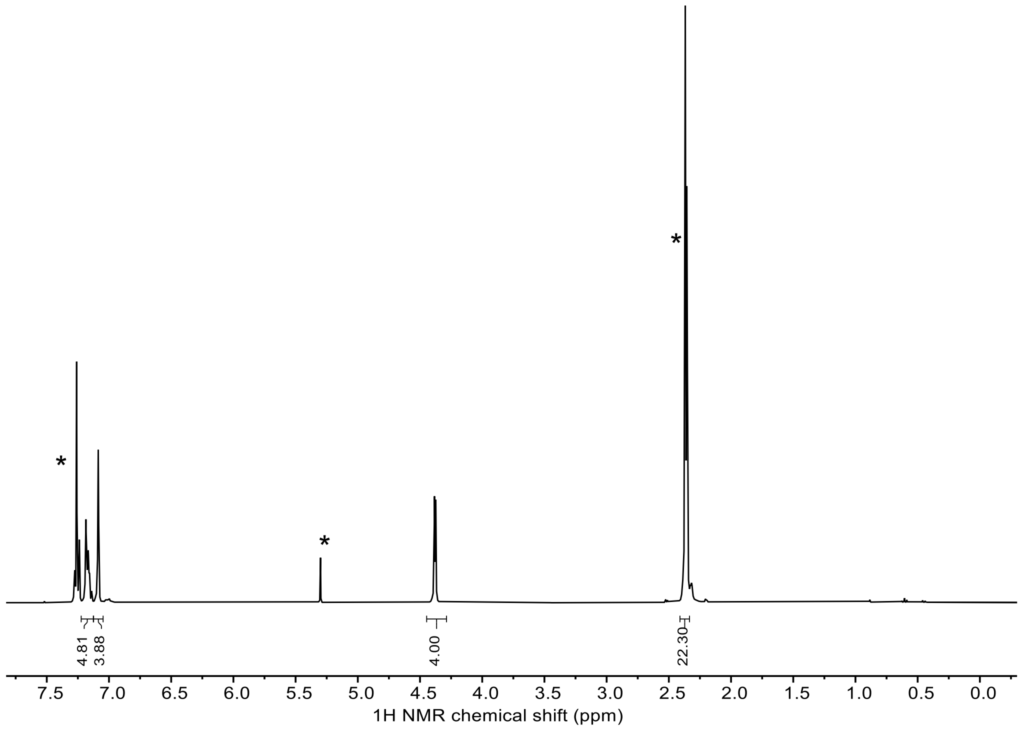

Figure S28. ^{1}H NMR spectrum of [MesNHP][$B(C_6F_5)_4$] in chloroform-*d*. Note the * signify toluene and dichloromethane (chloroform-*d* solvent) impurities. The toluene impurity Me signal overlaps with the Me signals for [MesNHP][$B(C_6F_5)_4$] at 2.36 ppm.

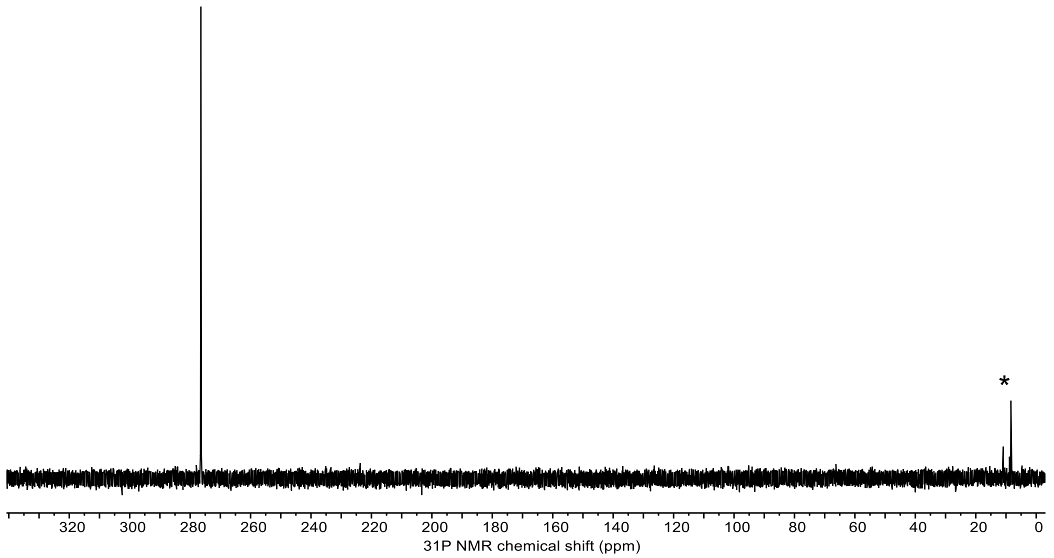

Figure S29. $^{31}P\{^{1}H\}$ NMR spectrum of [MesNHP][$B(C_6F_5)_4$] in chloroform-*d*. *denotes unknown minor impurities.

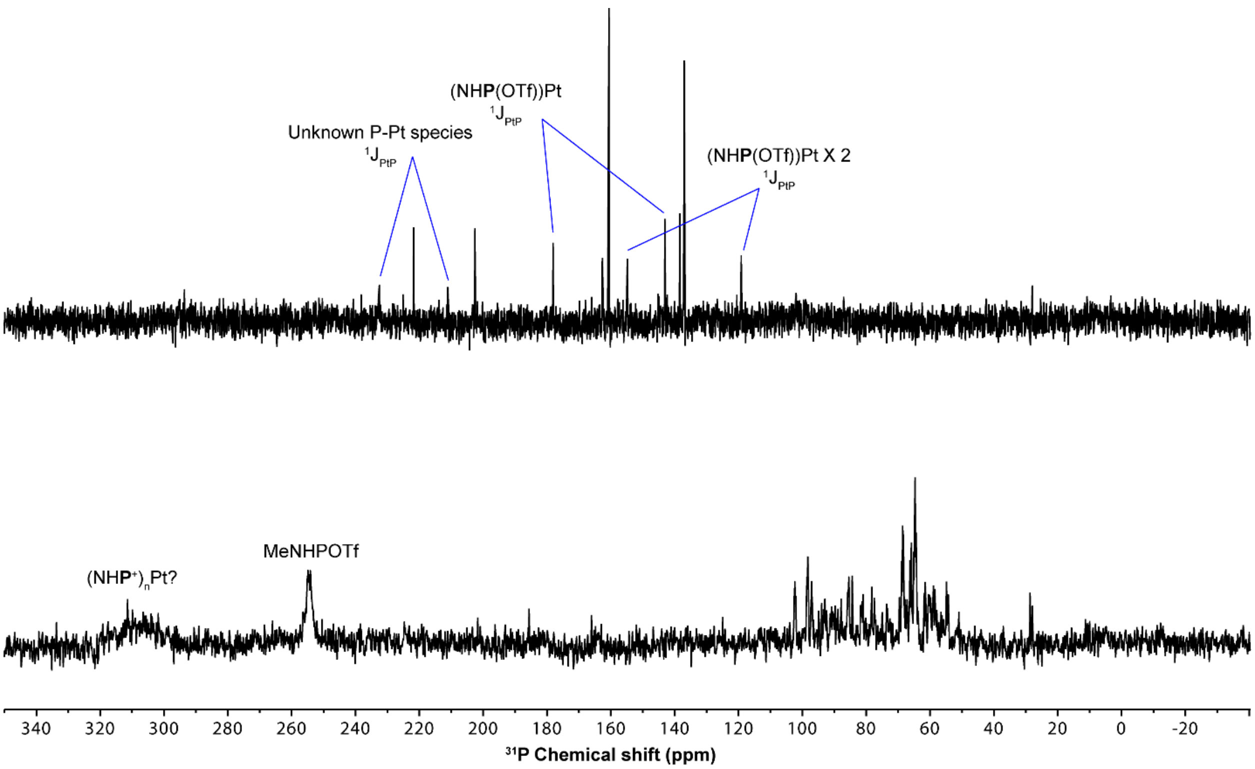

Figure S30. $^{31}P\{^{1}H\}$ NMR spectra of the reaction of Karstedt's catalyst with 1.2 (top) and 2.5 (bottom) equivalents of MeNHPOTf in dichloromethane-d_2.

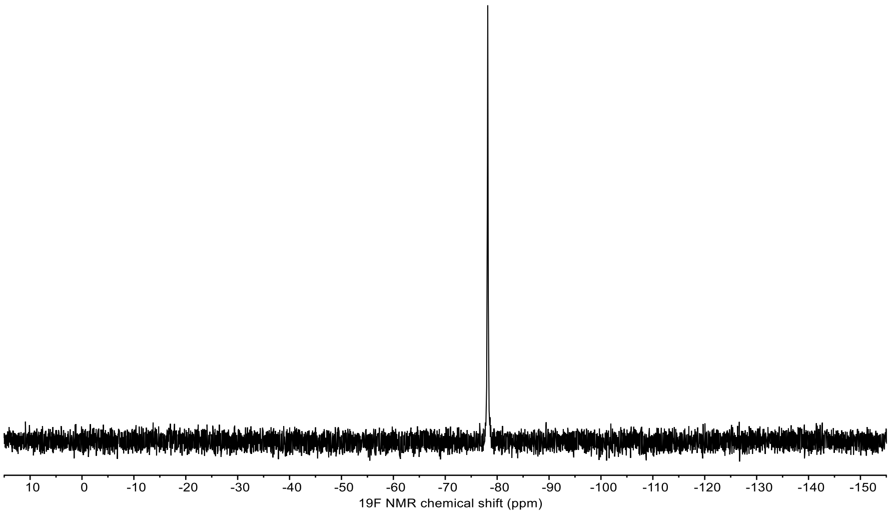

Figure S31. $^{19}F\{^{1}H\}$ NMR spectra of the reaction of Karstedt's catalyst with 1.2 equivalents of MeNHPOTf in dichloromethane-d_2.

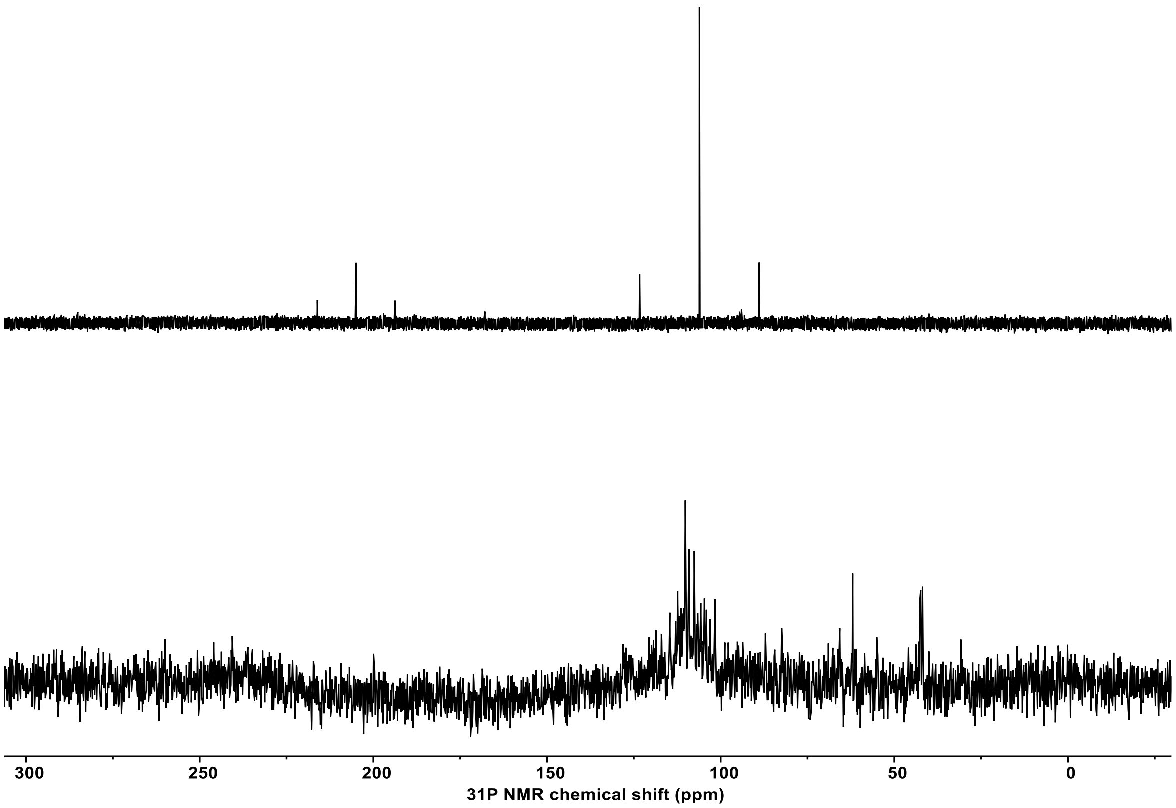

Figure S32. $^{31}P\{^{1}H\}$ NMR spectra of the reaction of Karstedt's catalyst with 1.2 (top) and 2.5 (bottom) equivalents of PhNHPOTf in dichloromethane-d_2.

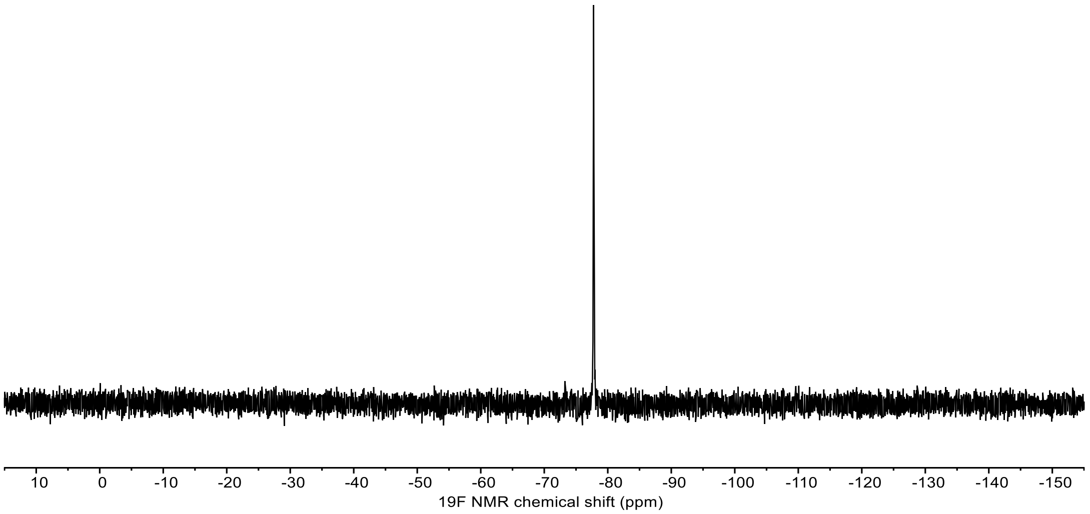

Figure S33. $^{19}F\{^{1}H\}$ NMR spectra of the reaction of Karstedt's catalyst with 1.2 equivalents of PhNHPOTf in dichloromethane-d_2.

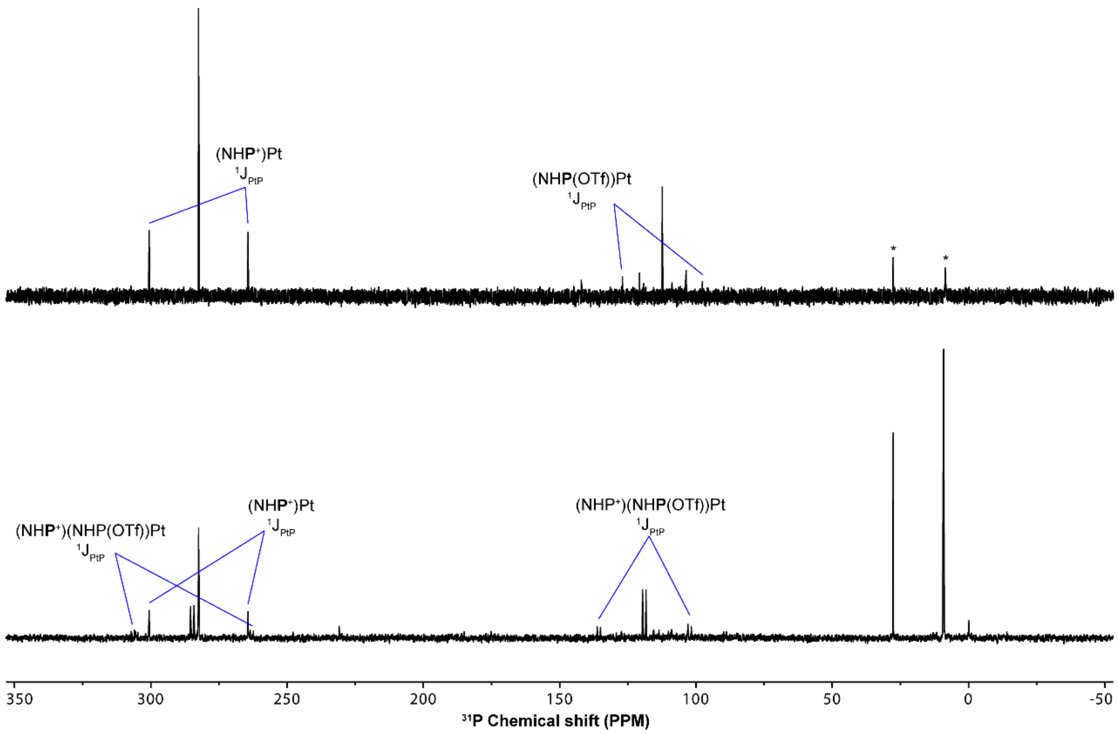

Figure S34. $^{31}P\{^1H\}$ NMR spectra of the reaction of Karstedt's catalyst with 1.2 (top) and 2.5 (bottom) equivalents of MesNHPOTf in dichloromethane-d_2. *denotes likely reaction products from MesNHPOTf and dvtms.

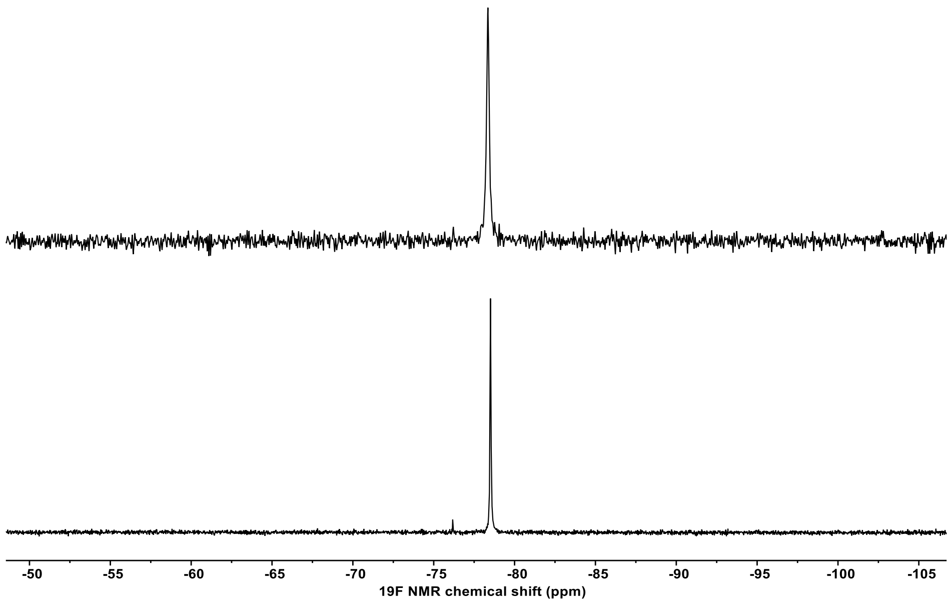

Figure S35. $^{19}F\{^1H\}$ NMR spectra of the reaction of Karstedt's catalyst with 1.2 (top) and 2.5 (bottom) equivalents of MesNHPOTf in dichloromethane-d_2.

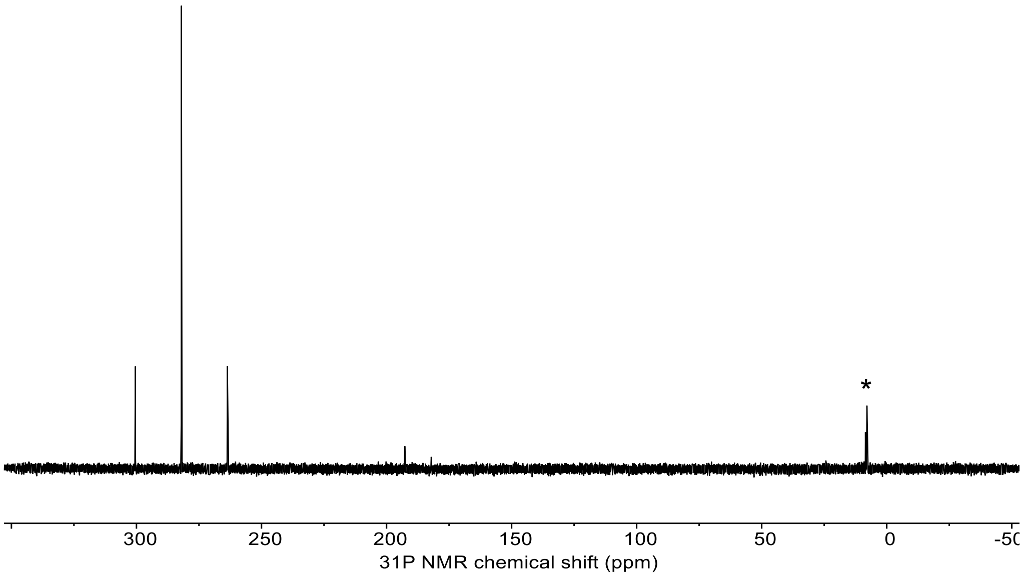

Figure S36. $^{31}P\{^1H\}$ NMR spectrum of the reaction of Karstedt's catalyst with 1.2 equivalents of [MesNHP][$B(C_6F_5)_4$] in dichloromethane-d_2. *denotes unknown minor impurities.

Catalytic reactions

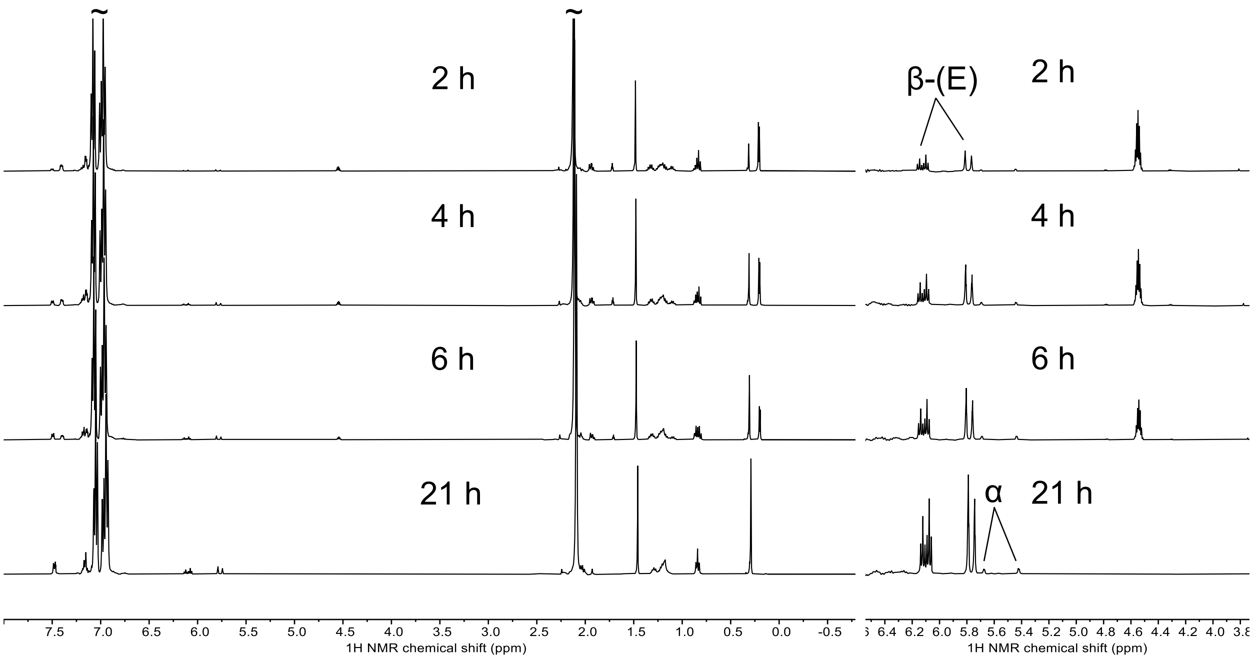

Figure S37. Representative ^{1}H NMR spectra of the reaction of 1-octyne with Me_2PhSiH catalyzed by 0.45 mol% **3d** over time in toluene with a benzene-d_6 capillary insert. The spectra are referenced to the toluene Me at 2.11 ppm. The right panel is a zoom in on the olefin and hydride region of the reaction. ~ = solvent signal cutoffs.

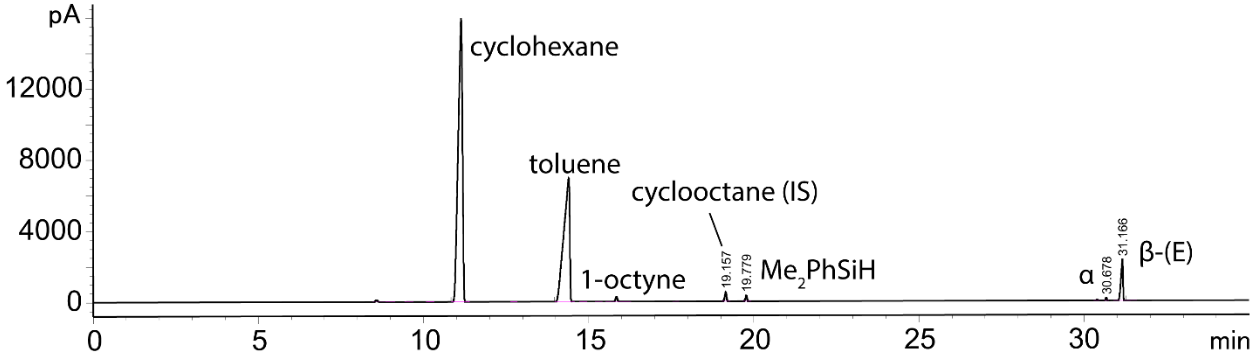

Figure S38. Representative gas chromatograph of the reaction of 1-octyne with Me_2PhSiH catalyzed by 0.45 mol% **3d** over time in toluene.

References

(1) Copéret, C.; Comas-Vives, A.; Conley, M. P.; Estes, D. P.; Fedorov, A.; Mougel, V.; Nagae, H.; Núñez-Zarur, F.; Zhizhko, P. A. Surface Organometallic and Coordination Chemistry toward Single-Site Heterogeneous Catalysts: Strategies, Methods, Structures, and Activities. *Chem. Rev.* **2016**, *116* (2), 323–421. https://doi.org/10.1021/acs.chemrev.5b00373.

(2) Ramirez, Fausto.; Patwardhan, A. V.; Kugler, H. J.; Smith, C. Page. Formation of Phosphorus-Oxygen Bonds in the Reactions of Triaminophosphines with o-Quinones, Vicinal Triketones, and Xoxmalonic Esters. Triaminooxyphosphonium Dipolar Ions and Triaminodioxaphosphoranes. Phosphorus-31 Nuclear Magnetic Resonance Spectra. *J. Am. Chem. Soc.* **1967**, *89* (24), 6276–6282. https://doi.org/10.1021/ja01000a051.

(3) Rice, N. T.; Popov, I. A.; Russo, D. R.; Bacsa, J.; Batista, E. R.; Yang, P.; Telser, J.; La Pierre, H. S. Design, Isolation, and Spectroscopic Analysis of a Tetravalent Terbium Complex. *J. Am. Chem. Soc.* **2019**, *141* (33), 13222–13233. https://doi.org/10.1021/jacs.9b06622.

(4) Robbie, A. J.; Cowley, A. R.; Jones, M. W.; Dilworth, J. R. Complexes of Sterically-Hindered Diaminophosphinothiolate Ligands with Rh(I), Ni(II) and Pd(II). *Polyhedron* **2011**, *30* (11), 1849–1856. https://doi.org/10.1016/j.poly.2011.04.041.

(5) Abrams, M. B.; Scott, B. L.; Baker, R. T. Sterically Tunable Phosphenium Cations: Synthesis and Characterization of Bis(Arylamino)Phosphenium Ions, Phosphinophosphenium Adducts, and the First Well-Defined Rhodium Phosphenium Complexes. *Organometallics* **2000**, *19* (24), 4944–4956. https://doi.org/10.1021/om0005351.

(6) Mazieres, M. R.; Roques, C.; Sanchez, M.; Majoral, J. P.; Wolf, R. Chlorophospheniums, Precurseurs de Nouveaux Cations Du Phosphore Dicoordonne. *Tetrahedron* **1987**, *43* (9), 2109–2118. https://doi.org/10.1016/S0040-4020(01)86792-2.

(7) Caputo, C. A.; Price, J. T.; Jennings, M. C.; McDonald, R.; Jones, N. D. N-Heterocyclic Phosphenium Cations: Syntheses and Cycloaddition Reactions. *Dalton Trans.* **2008**, No. 26, 3461–3469. https://doi.org/10.1039/B801684D.

(8) Tafazolian, H.; Culver, D. B.; Conley, M. P. A Well-Defined Ni(II) α-Diimine Catalyst Supported on Sulfated Zirconia for Polymerization Catalysis. *Organometallics* **2017**, *36* (13), 2385–2388. https://doi.org/10.1021/acs.organomet.7b00402.

(9) Culver, D. B.; Conley, M. P. Activation of C−F Bonds by Electrophilic Organosilicon Sites Supported on Sulfated Zirconia. *Angew. Chem. Int. Ed.* **2018**, *57* (45), 14902–14905. https://doi.org/10.1002/anie.201809199.

(10) Foley, E. A.; Thuma, J. F.; Mayer, J.; Halder, M.; Huang, W.; Perras, F. A.; Culver, D. B.; Kobayashi, T. Protecting Air/Moisture-Sensitive Samples Using Perdeuterated Paraffin Wax for Solid-State NMR Experiments under Magic-Angle Spinning. *J. Magn. Reson.* **2025**, *379*, 107935. https://doi.org/10.1016/j.jmr.2025.107935.

(11) Nishiyama, Y.; Agarwal, V.; Zhang, R. Efficient Symmetry-Based γ-Encoded DQ Recoupling Sequences for Suppression of *t*1-Noise in Solid-State NMR Spectroscopy at Fast MAS. *Solid State Nucl. Magn. Reson.* **2021**, *114*, 101734. https://doi.org/10.1016/j.ssnmr.2021.101734.

(12) Jammee, R.; Kolganov, A.; Groves, M. C.; Pidko, E. A.; Sydora, O. L.; Conley, M. P. C−H Bond Activation by Sulfated Zirconium Oxide Is Mediated by a Sulfur-Centered Lewis Superacid. *Angew. Chem. Int. Ed.* **2025**, *64* (11), e202421699. https://doi.org/10.1002/anie.202421699.
(13) Jabbour, R.; Renom-Carrasco, M.; Chan, K. W.; Völker, L.; Berruyer, P.; Wang, Z.; Widdifield, C. M.; Lelli, M.; Gajan, D.; Copéret, C.; Thieuleux, C.; Lesage, A. Multiple Surface Site Three-Dimensional Structure Determination of a Supported Molecular Catalyst. *J. Am. Chem. Soc.* **2022**, *144* (23), 10270–10281. https://doi.org/10.1021/jacs.2c01013.
(14) Nava, M.; Reed, C. A. Triethylsilyl Perfluoro-Tetraphenylborate, [Et3Si+][F20-BPh4–], a Widely Used Nonexistent Compound. *Organometallics* **2011**, *30* (17), 4798–4800. https://doi.org/10.1021/om200636u.
(15) Culver, D. B.; Mais, M.; Kang, M.-C.; Zhou, L.; Perras, F. A. Well-Defined Pt(0) Heterogeneous Hydrosilylation Catalysts Supported by a Surface Bound Phosphenium Ligand. *Dalton Trans.* **2025**, *54* (21), 8392–8399. https://doi.org/10.1039/D5DT00680E.
